# The Transcriptional Program of *Staphylococcus aureus* Phage K Is Affected by a Host *rpoC* Mutation That Confers Phage K Resistance

**DOI:** 10.3390/v16111773

**Published:** 2024-11-13

**Authors:** Rohit Kongari, Melissa D. Ray, Susan M. Lehman, Roger D. Plaut, Deborah M. Hinton, Scott Stibitz

**Affiliations:** 1 Center for Biologics Evaluation and Research, United States Food and Drug Administration, Silver Spring, MD 20993, USA; 2 Gene Expression and Regulation Section, Laboratory of Biochemistry and Genetics, National Institute of Diabetes and Digestive and Kidney Diseases, National Institutes of Health, Bethesda, MD 20892, USA

**Keywords:** bacteriophages, phage resistance, methicillin resistant *Staphylococcus aureus*, phage transcriptomics, RNA-sequencing

## Abstract

To better understand host–phage interactions and the genetic bases of phage resistance in a model system relevant to potential phage therapy, we isolated several spontaneous mutants of the USA300 *S. aureus* clinical isolate NRS384 that were resistant to phage K. Six of these had a single missense mutation in the host *rpoC* gene, which encodes the RNA polymerase β’ subunit. To examine the hypothesis that mutations in the host RNA polymerase affect the transcription of phage genes, we performed RNA-seq analysis on total RNA samples collected from NRS384 wild-type (WT) and *rpoC*_G17D_ mutant cultures infected with phage K, at different timepoints after infection. Infection of the WT host led to a steady increase of phage transcription relative to the host. Our analysis allowed us to define 53 transcriptional units and to categorize genes based on their temporal expression patterns. Predicted promoter sequences defined by conserved −35, −10, and, in some cases, extended −10 elements, were found upstream of early and middle genes. However, in many cases, sequences upstream of late genes did not contain clear, complete, canonical promoter sequences, suggesting that factors in addition to host RNA polymerase are required for their expression. Infection of the *rpoC*_G17D_ mutant host led to a transcriptional pattern that was similar to that of the WT at early timepoints. However, beginning at 20 min after infection, transcription of late genes (such as phage structural genes and host lysis genes) was severely reduced. Our data indicate that the *rpoC*_G17D_ mutation prevents the expression of phage late genes, resulting in a failed infection cycle for phage K. In addition to illuminating the global transcriptional landscape of phage K throughout the infection cycle, this study will inform our investigations into the basis of phage K’s control of its transcriptional program as well as mechanisms of phage resistance.

## 1. Introduction

*Staphylococcus aureus* is both a commensal bacterium and a serious human pathogen that causes a wide range of clinical illnesses [1], and about 30% of the human population are estimated to be asymptomatic nasal carriers of *S. aureus* [2,3]. The past 30 years have seen sharp increases in the incidence of staphylococcal diseases in healthcare and community settings, with the majority being caused by methicillin-resistant *S. aureus* (MRSA). Growth in the incidence of multidrug-resistant (MDR) bacteria, not just *S. aureus*, is a major public health concern [4,5,6,7]. In parallel, the discovery and development of novel antimicrobials has slowed dramatically over the past few decades [8,9]. This has led to a renewed interest in phage therapy, a practice that preceded the discovery of antibiotics [10,11]. The use of phages has been pursued as a potential treatment for bacterial infections in humans and animals, as well as for biocontrol in agri-food settings [12,13]. While interest in phage therapy waned in the US after the widespread implementation of antibiotics, it remained a common modality in the Soviet Union and some eastern European medical communities. Animal studies have suggested that phages may be able to help clear bacterial infections and improve survival rates [14]. In humans, unambiguous evidence of efficacy in controlled clinical studies is lacking, although there are anecdotal reports that appear to suggest successful phage treatment of some life-threatening infections [15,16].

In an effort to better understand aspects of phage biology that might be relevant to phage therapy, we are studying the interactions between *S. aureus* and myophages that infect it. Myophages of the *Twortvirinae* subfamily, owing to their obligately lytic lifestyle and broad host range, have been frequently investigated for use in phage therapy against staphylococcal infections [17,18,19]. Bacteriophage K, the most studied member of the genus *Kayvirus*, has been the subject of numerous studies analyzing the activity of phage therapeutics on a range of MRSA clinical isolates [20,21,22,23]. The phage K unit genome is 139,831 bp long with 212 predicted protein-coding genes (including three intron-encoded endonuclease genes) and four tRNA genes (Met, Trp, Phe, and Asp) [24,25]. Phage K possesses a terminally redundant, non-circularly permuted genome, like other members of the *Herelleviridae* family, and is packaged as a 148,317 bp-long genome that includes 8486 bp long terminal direct repeats. All members of the *Kayvirus* genus are similar in genome size, coding density, and gene organization [26].

Recent studies have employed RNA-seq as a tool to study host–phage interactions, especially the dynamics of phage gene expression and the resulting changes in the global transcriptome of the host [27]. Infection with virulent phages has been investigated using RNA-seq in Gram-negative hosts such as *Pseudomonas*, *Yersinia*, *Vibrio*, and *Escherichia,* as well as in Gram-positive hosts such as *Mycobacterium* and *Staphylococcus* [28,29,30,31,32,33,34]. Kornienko et al. provided the first insights into the transcriptional landscape of *Kayvirus* phages during infection, using the phage vB_SauM-515A1 and *S. aureus* strain SA515 as a host [35]. Another study, by Finstrlova et al., focused on the differential transcription of phage K infecting the prophage-less *S. aureus* strain SH1000 and a lysogenic strain that harbors four prophages, *S. aureus* Newman [36]. All three of these host strains were methicillin-sensitive strains belonging to multi-locus sequence type 8 (ST8). In this study, we used strand-specific RNA-seq to examine the progress of gene expression in phage K during infection of NRS384, a clinical *S. aureus* isolate belonging to the USA300 MRSA sublineage of ST8. We also compared the transcriptional profiles of phage K at different stages of infection in an NRS384_WT_ host to those in a phage-resistant mutant carrying a mutation in the host RNA polymerase (RNAP) β’ subunit gene (*rpoC*). Our work provides the first reported example of mutations in the *Staphylococcus* host RNA transcription machinery that affect phage K gene expression and phage growth. Our data indicate that expression of late genes that code for structural components and lysis in phage K is severely impeded in the mutant, offering key insights into potential mechanisms through which *Staphylococcus* hosts could gain resistance to phage K and related phages. Given the widespread use of *Kayvirus* phages in investigational therapeutic phage cocktails for treatment of staphylococcal infections [19,22,23] and the clinical relevance of the NRS384 host and other USA300 MRSA strains [4,37,38], this analysis provides important new insight into molecular interactions between *S. aureus* and its phages.

## 2. Materials and Methods

### 2.1. Bacterial Strains, Phages and Growth Conditions

*S. aureus* NRS384 (USA300-0114 CA-MRSA) was obtained from the NARSA collection. ATCC 19,685 (the host used for preparing phage K lysates and calculating titers) and phage K were obtained from the American Type Culture Collection. The host bacteria were grown in tryptic soy broth (TSB) or on tryptic soy agar (TSA) plates at 30 °C and supplemented with 5 mM CaCl_2_ whenever phage was added. A phage K stock lysate was prepared by inoculating an ATCC 19,685 broth culture (at an OD_600_~0.3–0.5) and incubating at 30 °C after addition of phage at a multiplicity of infection (MOI) of ~0.1. After clearing, the lysate was harvested by centrifuging at 8000× *g* for 15 min and passing the supernatant through a 0.22 µm filter. Lysate titers were measured using either the spot titer or full plate titer method as previously described [39]. Phage-resistant NRS384 host mutants were isolated by spreading 100 µL of genetically independent overnight cultures onto TSA plates that were pre-spread with different dilutions of phage K stock (~10^7^, 10^6^, 10^5^ PFU per plate); plates were then incubated overnight at 30 °C. For each genetically independent overnight culture, we used the plate that contained the most phage while still yielding single colonies after overnight incubation. These single colonies were picked and re-streaked onto fresh TSA plates. A spot titer assay was performed to confirm the phage resistance phenotype and assess the relative efficiency of plaquing. Eighteen such phage-resistant colonies were chosen for further sequencing analysis and made into frozen permanent stocks.

Growth kinetic assays were run at 30 °C in a BioTek Cytation3 plate reader (Agilent Technologies, Santa Clara, CA, USA) using 96-well plates and a discontinuous kinetics program in which OD_600_ readings were collected every 15 min for 18 h. To examine the inhibitory effect of phage on bacterial growth, phage K was added at an MOI of 0.5 once the OD_600_ across all wells reached 0.2–0.3.

### 2.2. Adsorption Assay and One-Step Growth Curve

An adsorption assay was performed as described previously [39]. Briefly, *S. aureus* hosts (NRS384_WT_, MR202, MR203, MR 206, MR210, MR211, and MR214; two replicates each) were grown to an OD_600_ of ~0.5 in 25 mL TSB at 30 °C with shaking at 200 rpm. Five mL of these log-phase bacteria were harvested by centrifugation at 4500× *g* for 10 min and resuspended in 0.9 mL fresh TSB. One hundred µL of diluted phage stock (final MOI = 0.01) was added to each resuspended bacterial sample and to a bacteria-free control tube. The tubes were then incubated at 30 °C with shaking at 120 rpm, and 200 µL samples were collected in pre-chilled microcentrifuge tubes every 5 min after addition of phage. Samples were briefly vortexed and then immediately centrifuged for 10 min at 14,000 rpm at 4 °C. To quantify the number of unadsorbed phages at each timepoint, serial dilutions of the supernatant from each sample were spotted onto soft agar overlays of ATCC 19685. The percentage of unadsorbed phages was calculated relative to the plaque counts obtained from the bacteria-free control sample.

For one-step growth curve studies, ~10 mL broth NRS384_WT_ and *rpoC*_G17D_ cultures were grown in shaker flasks (200 rpm) at 30 °C to an OD of ~0.3 and infected with phage K at an MOI of 0.01. After 5 min of adsorption, unadsorbed phages were removed by centrifugation at 4500× *g* for 5 min. Harvested cells were resuspended in 5 mL fresh TSB and returned to the shaker incubator. Two-hundred µL samples were collected at an interval of every 5 min and added to pre-chilled microcentrifuge tubes with or without 4 µL chloroform (2% *v*/*v*). The tubes were quickly vortexed and centrifuged at 14,000 rpm for 90 s. To quantify the number of assembled phages (+chloroform) and released phages (−chloroform) at each timepoint, the collected supernatants were serially diluted and spotted on soft agar overlays of ATCC 19685. The experiment was repeated twice, and the average values were used to determine latency period and lysis time.

### 2.3. DNA/RNA Extraction and Sequencing

Bacterial genomic DNA was isolated from NRS384_WT_ and the phage-resistant mutants using the Epicentre MasterPure Gram Positive DNA Purification kit and sequenced using Illumina MiSeq (500 cycles) at Tufts University Core Facility Genomics. The locations of SNPs in the mutants were identified using the variant calling software VarScan2 [40], and SNPs were identified using TCH1516 (NC_010079) as a reference genome.

For transcriptomic analysis, four replicate 25 mL cultures of NRS384_WT_ and *rpoC*_G17D_ were grown to an OD_600_ of ~0.3, and phage K was added at an MOI of 5. One mL samples were collected at 2, 5, 10, 20, 30, and 40 min after addition of phage, and total RNA was extracted from these samples using the QIAGEN RNEasy Mini Kit. The samples were further treated with Turbo DNAse to degrade phage/bacterial DNA, and the RNA quality was measured using an Agilent 2100 Bioanalyzer (Agilent Technologies, Santa Clara, CA, USA). Ribosomal RNA was depleted using the Illumina Ribozero Plus kit, and Illumina RNA-seq libraries were prepared without any enrichment. A strand-specific RNA-seq Novaseq S2 100 bp paired-end run was performed on the samples using an Illumina Novaseq 6000 (Illumina, Hayward, CA, USA) at the University of Maryland School of Medicine Institute for Genomic Sciences.

### 2.4. RNA-Seq Data Analysis

The generated read data were uploaded to the CBER Galaxy platform, where the following bioinformatic analysis was conducted [41]: the reads from all the samples passed the initial quality check step conducted using FastQC v0.11.8 (https://www.bioinformatics.babraham.ac.uk/projects/fastqc/, accessed on 1 November 2024 ) and needed no trimming. Reads were mapped to the full-length genomes of phage K (NC_005880.2) and NRS384 (CP027476.1) using Bowtie2 [42], and mapped reads were visualized using IGV [43]. The distribution of reads mapping to the host vs. the phage was calculated using the MultiQC tool [44].

Identification of putative promoter sequences was performed in two ways. Visual inspection (necessarily confined to sequences upstream of, and/or intergenic to, phage ORFs) allowed the identification of obvious sequence motifs typically associated with promoter function (−35, −10, extended −10). In addition, the entire phage genome was subjected to analysis with the PhagePromoter [45] and ARAGORN [46] tools. The results were combined, and putative promoter sequences that met the following criteria are listed in Table S1: presence of a 35 sequence that had at least three out of six bp in common with the consensus and at least one of the conserved residues underlined here: TTGACA; presence of a −10 sequence with at least five of six bp in common with the consensus and at least two of the three conserved residues underlined here: TATAAT; and spacing of 16–18 bp between the −35 and the −10. As shown in the table, many putative promoters contained the extended −10 sequence TG spaced one bp upstream of the −10. This sequence has been shown to be able to compensate for the lack of a functional −35 region and, in the presence of the other two consensus elements, can increase promoter strength [47]. Consensus sequences for the promoters governing these transcriptional units were produced using WebLogo [48].

For inclusion in Table S1, potential transcription terminators were identified by the presence of short inverted repeats, annotated as “regulatory region[s]” in the phage K reference genome (NC_005880.2), and then adjusted to conform, to the extent possible, with constraints described by Roberts [49] for rho-independent terminators. Initially, transcriptional units (TUs) were defined as sets of genes lying between two predicted terminators. Although a TU defined in this way might contain multiple promoters, overlapping transcription initiating at any of them would be predicted to end at the downstream terminator. However, in a few cases, we observed that not all genes within TUs defined in this way were expressed in the same temporal patterns or at the same approximate levels of transcription. These observations suggested the presence of additional regulatory sites, and in these cases, additional TUs were delineated. To label a TU as early, middle, or late, we considered whether one or more genes within it fell into one of the four groupings defined by Clust analysis, as well as visual impressions of the overall pattern of transcription over the course of infection. The Clust method was prioritized, where applicable, with early, middle, and late TUs corresponding to groups C1, C2+C3, and C4, respectively.

Read counts mapping to annotated gene features were quantified using Kallisto [50], the principal component analysis and differential expression analyses were performed using the tidyverse, plotly, and edgeR packages [51] on the RStudio platform v4.2.2 (Rstudio: Integrated Development for R. Rstudio, PBC, Boston, MA, USA; http://www.rstudio.com, accessed on 1 November 2024). Changes in gene expression levels between consecutive timepoints were measured in log_2_FC units and fit to the lmfit model to evaluate statistical significance. Co-expression clusters were predicted using the Clust package [52] (method = ‘k-means’, tightness weight = ‘0.5’, rep. info = ‘101 3 4’), and the resulting z-scores vs. time were plotted for each cluster using Excel.

## 3. Results

### 3.1. Isolation and Initial Characterization of rpoC Mutants

In order to define potential mechanisms of resistance to phage K, we initially isolated 18 genetically independent mutants of *S. aureus* strain NRS384 that could form colonies on plates seeded with phage K and on which phage K could not form plaques at 30℃ on trypticase soy agar (TSA). Genomic DNA was isolated from each of these mutants and subjected to whole-genome sequencing. Variant analysis revealed that 7 of the 18 phage-resistant mutants (MR202, 203, 205, 206, 209, 210, and 211) had mutations in the *rpoC* gene, which encodes the host DNA-directed RNAP β’ subunit (Table 1). In six of these, the *rpoC* mutation was the only difference found between the mutant and WT genomes (Table 1). The presence of the identified mutation in each strain was confirmed by Sanger sequencing of PCR fragments from the relevant regions of the *rpoC* gene. All the mutations in *rpoC* were found at three positions (Gly17, Gly70, and Ala267). Even though they were isolated in such a way as to ensure that they arose independently, two mutations were isolated twice (G17D and A267E). This suggests that only a fairly small set of mutations can interfere with phage replication while still maintaining *rpoC* function adequate for normal cell growth in the absence of phages. Interestingly, substitutions in G17 with a non-polar (G17V), a positively charged (G17R), or a negatively charged (G17D) residue led to phage resistance, highlighting the importance of this residue.

**Figure 1:**
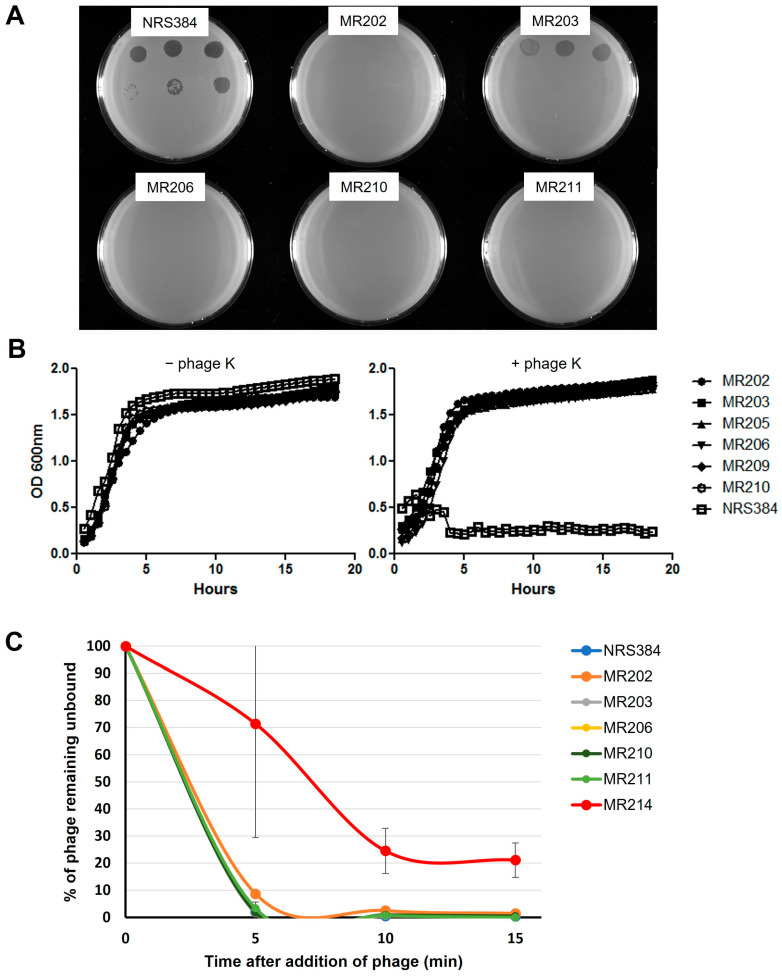
Initial characterization of the NRS384 *rpoC* mutants resistant to phage K. (**A**) Plaquing phenotype of phage K on NRS384_WT_ host and resistant mutants. Serial dilutions of phage K stock were spotted onto bacterial agar overlays of the wild-type and different mutant hosts and incubated overnight at 30 °C. (**B**) Growth curves of NRS384_WT_ host and different *rpoC* mutants in the absence (left) and presence (right) of phage K at 30 °C. (**C**) Adsorption efficiency of phage K to different *rpoC* hosts at 30 °C, shown as the percentage of unbound phage remaining in the supernatant.

In spot titer tests, none of the *rpoC* mutants allowed formation of discrete phage K plaques (Figure 1A), although high phage titers appeared to partially inhibit growth of MR203 (G70E) on plates. All the *rpoC* mutants exhibited growth kinetics similar to NRS384_WT_ in 96-well plate broth cultures and were unaffected by the addition of phage K at a titer that completely suppressed the growth of NRS384_WT_ at 30 °C (Figure 1B). There was also no significant difference in the adsorption efficiency of phage K to any of the *rpoC* mutant hosts when compared to the parental NRS384_WT_ host (Figure 1C). As a binding control, we used a different phage K-resistant mutant, MR214, harboring the mutation *tagX*_S165R_. The *tagX* gene lies in a gene cluster that is responsible for WTA synthesis. Although its possible function as a glycosyltransferase has been debated, and a *tagX* deletion did not affect infection by the *S. aureus* phage 187 [53], we found that the *tagX*_S165R_ strain was less able to adsorb phage K from solution, indicating reduced phage binding. These differing results could reflect a difference between phages 187 and K or a difference between the gene deletion and the S165R point mutation. Regardless, it seems likely that *tagX* has a role in synthesizing the WTA structure that can affect phage K binding.

**Figure 2:**
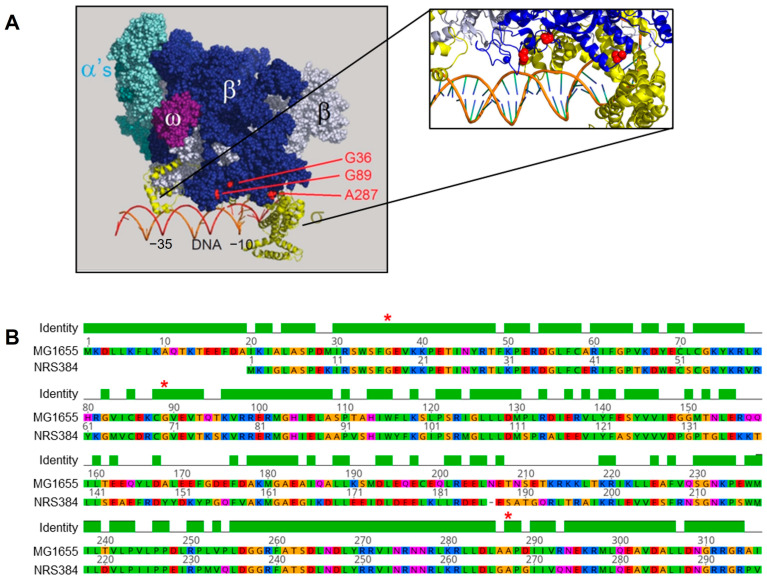
Spatial representation of *rpoC* mutations that confer phage K resistance. (**A**) Three-dimensional structure of RNA polymerase homolog from *E. coli* modeled together with fork junction DNA (orange). The alpha, beta, beta’, omega, and sigma subunits of RNAP are differently colored and labeled. Mutated amino acid residues are shown in red (adapted from PDB structure ID: 1L9Z [55]). (**B**) Alignment of *rpoC* b’ subunit sequences (first 300 amino acids) from *S. aureus* NRS384 and *E. coli* MG1655 showing conserved regions in the N-terminal domain (amino acids are highlighted according to the Clustal color scheme). Positions of residues mutated in the phage-resistant *rpoC* mutants are indicated by red asterisks above the identity bars.

To understand the potential roles of the three mutated positions in *S. aureus rpoC*, we identified the corresponding residues in the *E. coli* RNAP β’ subunit and mapped them onto the available structure of a complex of *E. coli* σ70-RNAP containing promoter DNA [54] (Figure 2A). The amino acid sequence alignment of the *E. coli* and *S. aureus* β’ subunits (Figure 2B) indicated that, within the regions encompassing the sites of the *rpoC* mutations, sequence conservation (and presumably structural conservation) is very high. We hypothesized that these mutations confer resistance to phage K by interfering with transcription of phage genes that are essential for successful completion of the phage infection cycle, possibly by preventing interaction with a phage-encoded transcription factor.

### 3.2. Global Analysis of RNA-Seq Data from Phage K Infection in WT and Phage-Resistant Host

We employed RNA-seq to study the transcription kinetics of phage K genes during infection of NRS384_WT_ and the phage-resistant mutant MR206 (*rpoC*_G17D_). To choose timepoints for our RNA-seq analysis, we first performed a one-step growth curve of phage K infection of NRS384 (Figure S1) and found that the phage developmental cycle, as indicated by spontaneous release of phage particles or by an increase in the amount of phage released by chloroform treatment, was essentially complete by 40 min after infection. Based on this observation, we chose to extract RNA from the infected cells at seven different timepoints (0, 2, 5, 10, 20, 30, and 40 min) after infection. We infected these cultures at a ratio of five phage per bacterial cell to ensure that the majority of host cells were infected at the same time. Sample collection, total RNA extraction, and rRNA depletion for each timepoint and bacterial host were performed on four independent cultures and infections. The samples were sequenced using the Illumina platform, and the resulting RNA-seq reads were aligned to the phage K reference genome (NC_005880.2) and the host NRS384 reference genome (CP027476.1).

**Figure 3:**
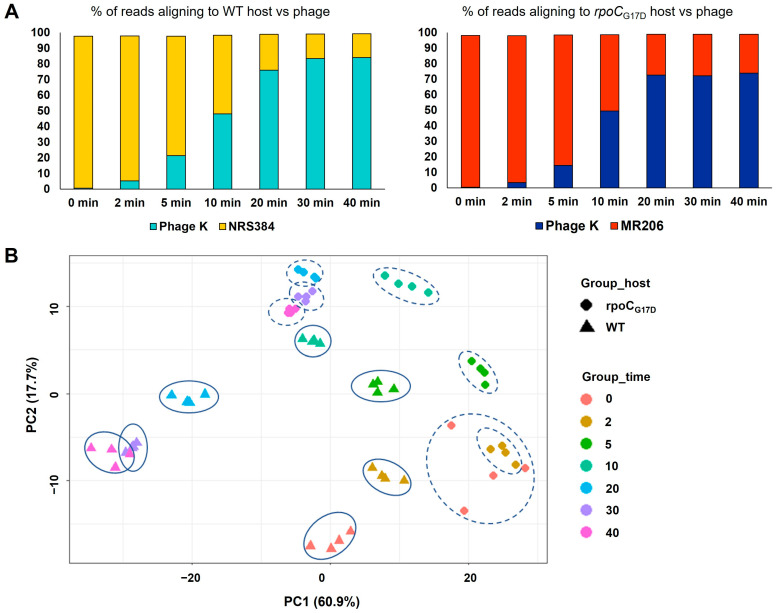
MultiQC and principal component analysis of RNA-seq reads from samples of phage K infection of WT and *rpoC* host. (**A**) Distribution of percentage of reads, calculated as an average of four replicates, mapping to the phage and host at different infection timepoints. The left panel shows phage (turquoise) vs. bacterial (yellow) reads in the NRS384_WT_ host infection. The right panel shows phage (dark blue) vs. bacterial (red) reads in the phage-resistant *rpoC*_G17D_ host infection. (**B**) Principal component analysis of total reads mapping to the phage genome from phage K infections of NRS384_WT_ (triangles) and *rpoC*_G17D_ (diamonds) hosts collected at different timepoints (colors indicated in the legend). Each plotted point refers to a replicate, and the replicates for each timepoint are grouped within an ellipse (solid line for NRS384_WT_ and dotted line for *rpoC*_G17D_).

During infection of NRS384_WT_ (Figure 3A), the percentage of reads mapping to the phage genome increased progressively from the first non-zero timepoint (2% at 2 min) to the last timepoint (84% at 40 min). In a complementary fashion, the percentage of reads mapping to the host genome decreased over the course of infection until the end of the experiment (15% at 30 or 40 min). These data indicate a progressive takeover of the host transcription machinery by the phage, consistent with observations made by others [35,36,56]. Our results are also consistent with findings from a previous study of *S. aureus* NCTC 9318 infection with phage K, which described the timeline of inhibition of host DNA synthesis followed by DNA degradation and incorporation of nucleotides into phage DNA [57], which may itself contribute to the swift decrease in overall host gene expression. For the *rpoC*_G17D_ host infected with phage K, the fraction of reads mapping to the phage initially increased at a similar rate as in the WT infection but then did not increase after the 20 min timepoint, remaining at a maximum of 72% (Figure 3A).

Principal component analysis of total reads mapping to the phage genome from both the NRS384_WT_ infection and *rpoC*_G17D_ infection (Figure 3B) showed that replicates corresponding to each timepoint for each host clustered together, indicating minimal variance within each set. However, there were significant differences in how infection progressed in the two hosts. For example, there was significant variance along both PC axes among all the timepoints for the NRS384_WT_ infection, implying that different sets of genes contributed to the variance in expression profiles at different times. The smallest variance was observed between the 30 and 40 min timepoints, suggesting that a similar set of genes was expressed at these two timepoints, as would be expected toward the end of the infection cycle. In contrast, during the *rpoC*_G17D_ infection, little variance was observed among the 20, 30, and 40 min timepoints, suggesting that the expression profile did not change substantially after 20 min. This is consistent with both the absence of progeny phages detected in this infection and the lack of an increase in global phage-specific transcription after 20 min, as noted above.

### 3.3. Transcriptional Program of Phage K Infection of the Wild-Type Host

**Figure 4:**
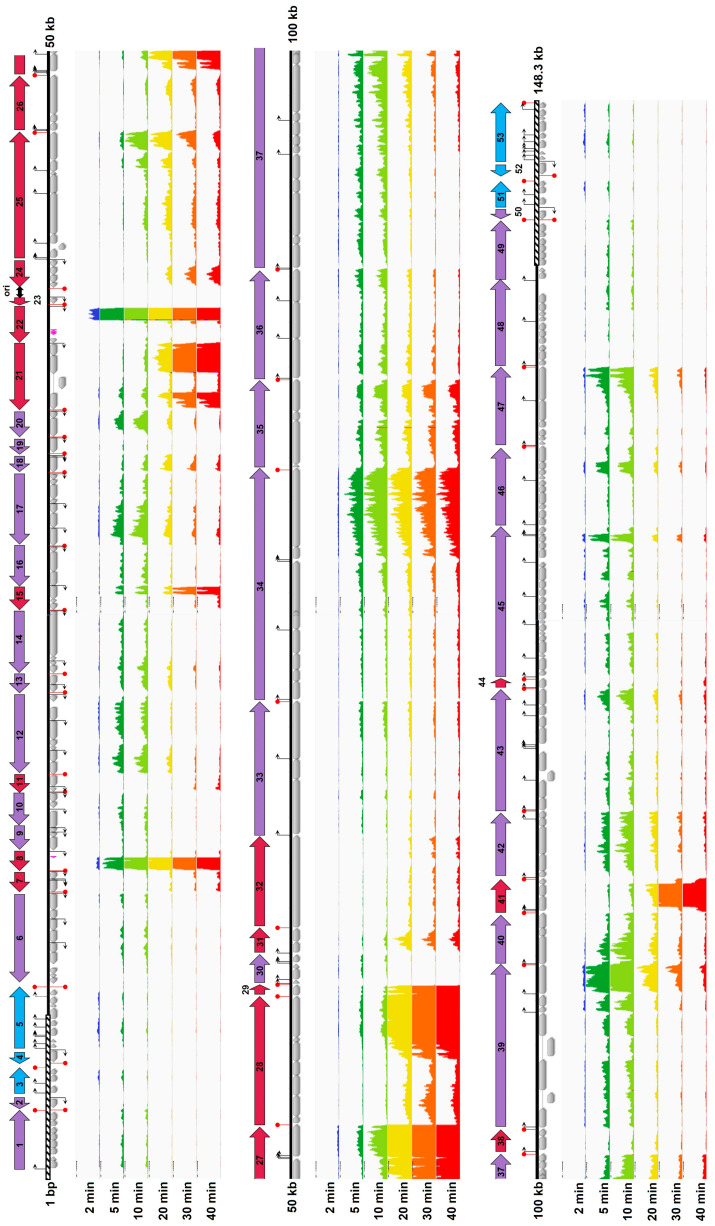
The global transcriptional profile of phage K at different timepoints after infecting NRS384_WT_ host. Visualization of reads mapping to the phage genome at different timepoints from a representative sample of the four replicates. The panels are arranged from top to bottom in order of increasing time after infection (2 min, dark blue; 5 min, dark green; 10 min, light green; 20 min, yellow; 30 min, orange; 40 min, red). The upper limit for the *Y*-axis in each panel is 10^6^ counts. The protein-coding genes and tRNA genes are shown in grey and pink, respectively, and the long terminal repeats are indicated by the bars with diagonal shading. Predicted promoter and terminator locations are denoted by arrows and red circles, respectively. The proposed transcriptional units are indicated by thick arrow bars (light blue, early; purple, middle; maroon, late).

To examine the temporal progression of gene expression through the infection cycle in more detail, we used Integrative Genomics Viewer (IGV) to visualize read mapping to the phage K genome at different timepoints [43] (Figure 4). Reads were mapped to all the annotated feature regions in the phage genome, as well as a few unannotated regions (see below). Two direct repeats (AAAAAGTACGTATTTAGAAAATAAGGAG) that may serve as potential binding sites for a replication initiator protein occur between gp190 and gp191 (39,181–39,208 bp and 39,371–39,398 bp), between transcriptional units #23 and #24; marked as “ori” in Figure 4) [26]. This region showed minimal to no transcriptional activity across all the samples observed, consistent with a role as an origin of replication for phage K. It should be kept in mind that these data measure steady-state levels of transcripts, not transcriptional activity per se. These levels represent the balance between rates of synthesis and degradation, and the methods we employed cannot separate the contributions of each of these processes. Nevertheless, it was evident that at least the initiation of phage gene expression was programmed into different phases that could be broadly described as early, middle, and late.

As a first step in defining these phases, we attempted to group genes into transcriptional units (TUs). Additional details of how this was done and how TUs were designated as having early, middle, or late expression patterns is provided in Materials and Methods. The identification of putative promoter sequences, a key part of this process, was performed in two ways. Visual inspection (necessarily confined to sequences upstream of, and/or intergenic to, phage ORFs) allowed the identification of promoter sequences with good matches to motifs that are typically associated with the function of a primary sigma factor (sA), such as *E. coli* s70. These include the −35 element (^−35^TTGaca^−30^), a spacer of 17 +/− 1 bp, a −10 element (^−12^TAtaaT^−7^), and the extended −10 element (^−15^TG^−14^) [47] (Numbers indicate positions relative to the +1 transcription start site; consensus sequences are given with less conserved bases in lower case). In addition, the entire phage genome was analyzed using the programs PhagePromoter and ARAGORN. Interestingly, each approach identified some promoters that were not detected by the other. For example, the bioinformatic approach identified candidates, typically within ORFs, that were not found by visual inspection, due to practical limitations of the latter approach. However, a number of candidates with very good matches to consensus elements and with proper spacing between them were missed by the program. Our experience underscores the limitations of promoter identification by sequence analysis software. Putative promoters that met our criteria, as described in Materials and Methods, are detailed in Table S1, along with the factor-independent terminator sequences annotated in the phage K reference genome (NC_005880.2).

Six transcriptional units, comprising 24 genes located in the long terminal repeats (gp010-021, and repeated as gp044-033), showed early transcriptional activity (Figure 4). Although most of the proteins encoded by these genes have no annotated function, their homologs in other virulent phages, termed terminal repeat encoded genes (TreA, TreB, etc.), have been implicated in host shutdown and takeover [58,59]. The majority of phage K protein-coding genes (149 of the 212 unique genes) had a middle gene expression pattern and could be assigned to 29 transcriptional units. Many of these genes encode proteins that are predicted to be involved in transcriptional regulation, nucleotide metabolism, and DNA replication. The tail morphogenesis module also showed transcriptional activity in the middle phase. The remaining 18 transcriptional units showed distinctively high levels of expression in the later stages of infection (20–40 min). The 51 genes belonging to this group included those predicted to be involved in structural morphogenesis (both head and tail) as well as lysis.

**Figure 5:**
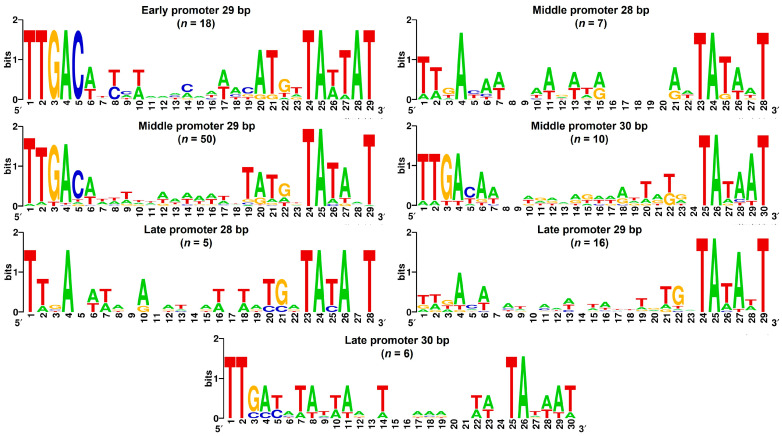
The promoters driving different stages of gene expression in phage K. WebLogo consensus sequences for early, middle, and late promoters in phage K, grouped by spacer length. Numbers indicate the position of the bases in the promoter element as annotated in Table S1, and the height of the bases indicates the degree of conservation at that particular position.

Temporal expression patterns of phage genes and TUs are expected to be the result of signals encoded within their promoters. To see whether candidates for such signals might be evident in the form of discrete sequence signatures, we examined alignments of promoter sequences for the different temporal classes (Table S1 and Figure 5). We found that promoters upstream of and within early and middle transcriptional units had features typical of host σA promoters, showing good agreement with consensus −35 and −10 elements (Figure 5) and a spacer of 16–18 bp between these elements. Extended −10 elements were frequently found in this context as well. However, promoter sequences upstream of late TUs or genes did not conform to this pattern. For some late TUs, predicted promoter sequences that met our criteria for inclusion in Table S1 were simply not found, either by computer analysis or visual scanning. One example of this is the TU28 containing gp172–gp166, which contains primarily structural genes. In other cases, a potential −10 element was present, sometimes together with the extended −10 dinucleotide. Alignment of these putative late gene promoter sequences, based on their −10 elements, failed to reveal −35 elements or other conserved features (Figure 5). These observations are consistent with data from earlier published phage K and phage vB_SauM-515A1 RNA-seq studies [35,36] and underscore our lack of understanding of late promoter structure/function relationships.

Our analysis of intergenic regions showed that transcription of two approximately 500 bp stretches lacking annotated open reading frames, termed long non-coding RNA (lncRNA), had the highest transcript abundance of any region of the genome (27% of the overall reads). The first region, lncRNA1 (528 bp), extends from bp 13,688 to bp 14,215, while the second, lncRNA2 (543 bp), lies between bp 38,024 and bp 38,566. We observed reads mapping to these lncRNA regions as early as 2 min, with their number increasing continuously through the infection cycle and with lncRNA2 being somewhat greater in number than lncRNA1 (Figure 4). BLAST analysis revealed that these lnc regions are highly conserved among other *Kayviruses* (Figure S2). Similar observations were made by Kornienko et al. in their RNA-seq analysis of phage vB_SauM-515A1 infecting *S. aureus* SA515 [35] and Finstrlova et al. in their studies of phage K infecting *S. aureus* strains SH1000 and Newman [36]. Surprisingly, given the very high level of expression of these lncRNAs, tRNA genes that are located downstream were much less highly transcribed, even though no typical transcriptional terminator or attenuator sequences [60] are evident that could explain this observation. Their evolutionary conservation and high level of expression suggest that the lncRNAs are likely to be of physiological relevance. However, the mechanistic bases of such a high level of transcription and its functional significance are unknown.

### 3.4. Differential Gene Expression and Clustering Analysis

To perform differential gene expression (DGE) analysis, we first calculated normalized transcripts per million (TPM) across all samples for each gene annotated in the genome of phage K. Analysis of the samples from the NRS384_WT_ host infection revealed a range of changes in transcript abundance for different genes across the course of infection (Table S2). For example, 31 of the 233 phage K genes showed at least a 10% change in abundance levels across every pair of consecutive timepoints measured in our experiment, until 30 min, at which point further changes were minimal. Only 18 predicted ORFs failed to show even this minimal change of 10% between at least one pair of consecutive timepoints. These 18 ORFs also had comparatively low transcript levels across all timepoints. Further investigation will be required to establish whether these are genes that actually contribute to a successful phage infection. On the other hand, 124 of the 233 genes showed a two-fold or greater change in abundance levels between at least one pair of consecutive timepoints. Genes belonging to the long terminal repeat transcriptional units showed the highest increase in expression in the first 2 min after infection, while most of the replication/transcription module genes underwent a positive log-fold change between the 2 min and 5 min timepoints. The structural and morphogenesis genes switched on between 5 and 10 min but exhibited a large increase in expression between the 10 min and 20 min timepoints. Nineteen structural genes, including the entire stretch of genes from gp165 to gp175, showed at least a 2.6-fold increase in expression during this transition period, implying a switching point from early to late phage gene expression.

**Figure 6:**
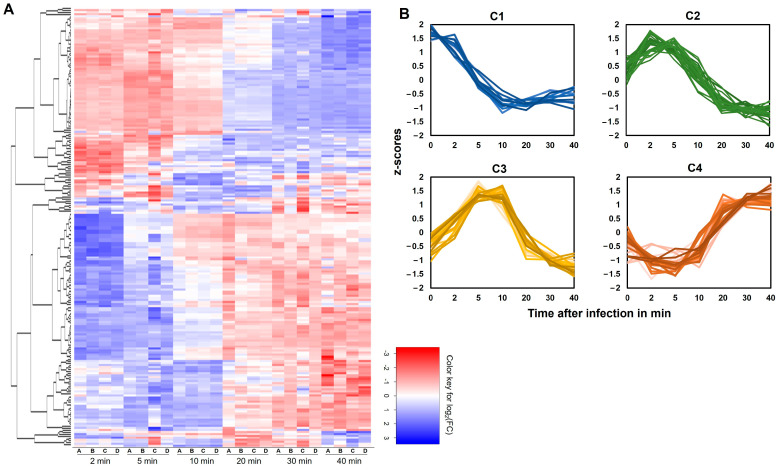
Differential expression analysis of phage K genes during infection of WT host. (**A**) Heatmap depicting log_2_-fold change between consecutive timepoints (2 min vs. 0 min, 5 min vs. 2 min, and so on until 40 min vs. 30 min) for all phage K genes (blue, increase; red, decrease) following infection of NRS384. Hierarchical clustering of genes based on temporal pattern of gene expression is shown on the *Y*-axis. The four replicate samples for each timepoint post-infection are labeled on the *X*-axis. (**B**) Co-expression clusters as identified by the Clust package. The *Y*-axis represents the z-scores calculated by Clust using normalized TPM data for each gene, accounting for all four replicate samples, and the time after infection is represented on the *X*-axis. Each line corresponds to the expression pattern of one gene belonging to the cluster.

A heatmap showing the fold change of expression between consecutive timepoints (Figure 6A) revealed clusters of genes being turned on and off at various stages of infection. We initially classified all genes as early, middle, or late based on visual evaluation of when transcription began, as shown in Figure 4. Then, to evaluate these transcriptional patterns in a more objective and quantitative way, we analyzed the TPM data using the Clust package, which is designed to extract clusters of genes that satisfy the biological expectations of co-expressed genes [52]. The Clust analysis produced four clusters of genes, termed C1–C4, within which genes showed similar and distinctive expression patterns (Table 2 and Figure 6B). The C1 cluster, consisting of 21 genes, was dominated by genes encoded within the long terminal repeat regions and showed maximum expression immediately after infection. The C2 cluster consisted of 30 genes that reached maximum expression around 2–5 min after infection. These included members of a putative metallophosphatase/phosphoesterase family that are involved in turnover of phosphoester-bound substrates and that could impact several key cellular processes [61,62]. The C3 cluster, attaining maximum expression approximately 5–10 min after infection, comprised 25 genes. These included genes involved in nucleotide metabolism and DNA replication. The C4 cluster included 33 genes, mostly related to head and tail morphogenesis and lysis. As would be expected, genes belonging to the C1 and C2 clusters demonstrated the highest abundance at early timepoints in our DGE analysis and decreased over time, whereas genes in C3 and C4 reached their maximum abundance at middle and later stages of infection.

### 3.5. Changes in Transcriptional Program of Phage K Infecting the rpoC_G17D_ Mutant

**Figure 7:**
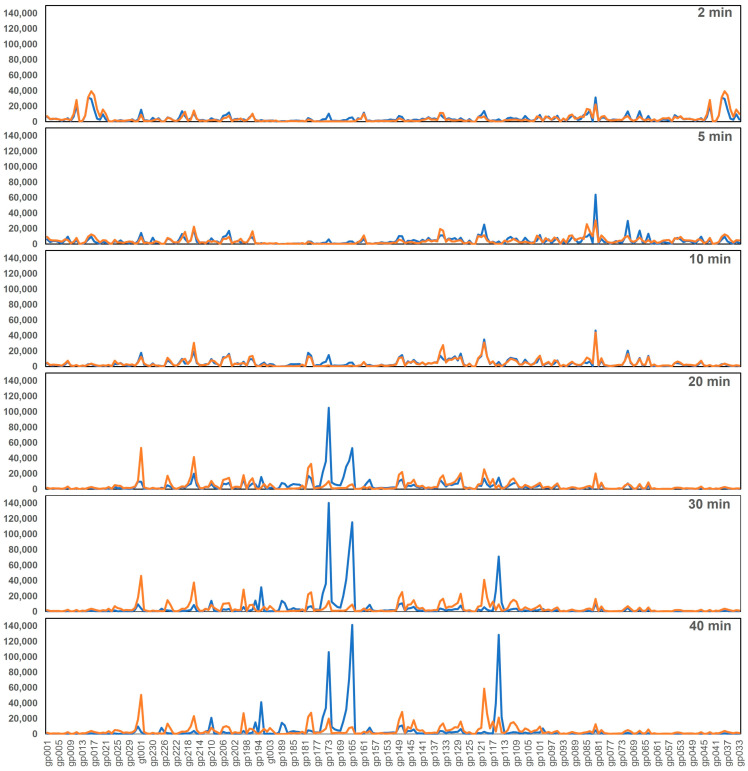
Effect of *rpoC*_G17D_ mutation on phage K transcriptional program. Visualization of differences in global transcription of phage K in *rpoC*_G17D_ (orange) compared to NRS384_WT_ (blue) at 10, 20, 30, and 40 min after infection. Only normalized count data of reads mapping to the annotated features (in the order they are structured in the phage genome) are shown.

**Figure 8:**
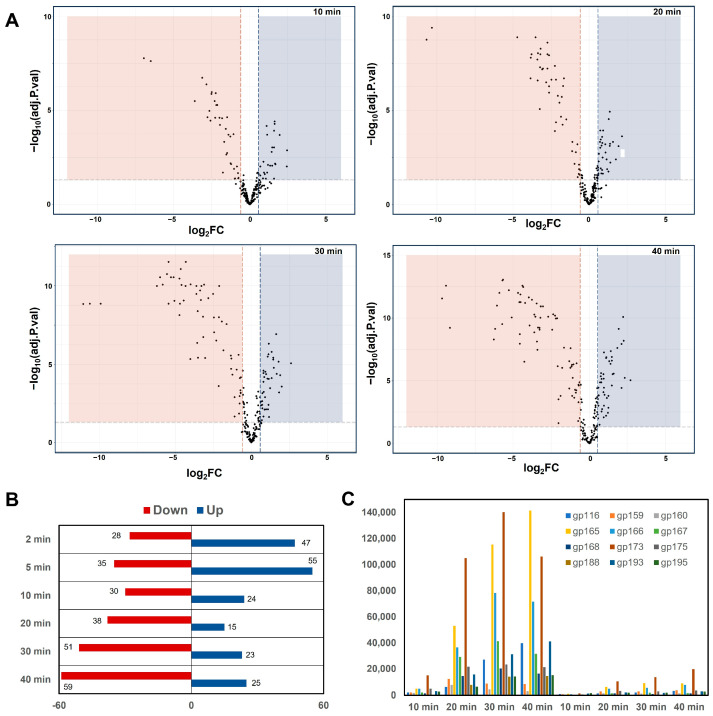
Differential expression analysis of phage K genes in *rpoC*_G17D_ mutant compared to NRS384_WT_ host. (**A**) Volcano plots showing the extent of differential expression of phage K genes in *rpoC*_G17D_ compared to NRS384_WT_. Log_2_-fold change is shown on the *X*-axis, and the log10-transformed adjusted *p*-value is shown on the *Y*-axis. The dots in the orange shaded region represent the genes showing significant (log_2_-fold change > 1) decreases in expression, whereas those in the blue shaded region show increases. (**B**) Number of genes differentially expressed (showing a log_2_-fold change of at least +1 or −1) during phage K infection of the *rpoC*_G17D_ mutant at different timepoints, when compared to WT infection. (**C**) Comparison of normalized counts for 12 selected genes from the C4 cluster at different timepoints during the course of infection of NRS384_WT_ (left) and *rpoC*_G17D_ (right). Bars for the different genes are color-coded as indicated and include gp116 (head decoration protein), gp159 (tail chaperone), gp160 (tail tape measure protein), gp165 (tail tube protein), gp166 (tail sheath protein), gp167 (hypothetical protein), gp168 (tail completion protein), gp173 (major capsid protein), gp175 (prohead protease), gp188 (putative membrane protein), gp193 (holin), and gp195 (endolysin).

To study the effects of the *rpoC*_G17D_ mutation on phage K transcription, we performed the same analyses as above on samples collected during phage K infection of the mutant host MR206. We observed that the G17D mutation significantly affected the transcription program of phage K, mainly resulting in the decreased expression of several late-expressed genes (Figure 7 and Figure 8). Notably, 11 of the late-expressed TUs (11, 21, 24–29, 31, 32, and 41) and 1 middle-expressed TU (34), accounting for 41 genes in total, were drastically affected across all time points. As shown in Table S1, these TUs include the lysis module, the head and tail morphogenesis modules, and the terminase large/small subunits. In addition, three more late TUs (15, 23, and 44) and three middle TUs (33, 35, and 45) were also downregulated after 20 min. These six TUs include additional tail morphogenesis genes. All these observations indicate that late gene transcription, which is essential for progeny virion assembly, viral replication, and lysis, is severely impeded in the *rpoC*_G17D_ host.

To more objectively quantify differences in the transcriptional activity of phage K in the mutant vs. the WT host, we performed a differential gene expression analysis comparing respective replicate samples for each timepoint (Table S3). Our results indicated that 146 phage K genes showed a log_2_FC greater than 1, either increased or decreased, at one or more of the timepoints measured (Figure 8A,B). Most of the genes that were downregulated in the *rpoC*_G17D_ mutant were from the structural morphogenesis module, and the effect was most significant at the 20 min and 30 min timepoints. TU24 seemed to be the most affected transcriptional unit, with drastic decreases in expression across almost all timepoints, and with a log_2_FC of approximately −5 at earlier timepoints and −10 across 20–40 min. Among the head structural genes, gp173 (log_2_FC of −4.5 at 2 min, −3.8 at 5 min, −3.6 at 10 min, −3.78 at 20 min, −5.1 at 30 min, and −4.5 at 40 min) and gp175 (log_2_FC of −3.2 at 2 min, −2.47 at 5 min, −3.1 at 10 min, −3.22 at 20 min, −4.2 at 30 min, and −4.5 at 40 min) were the most affected. The expression of almost all genes belonging to the C4 cluster identified in our analysis was either drastically decreased or not “turned on” in the mutant, with some genes showing a log_2_FC from −5 to −10 at certain timepoints of infection (Figure 8C). Expression of the early and middle genes/TUs was largely unaffected, although the block in late transcription may have indirectly resulted in increased expression of some genes.

Overall, our findings show that the switch to late phage gene expression does not occur in the *rpoC*_G17D_ mutant. Defective transcription of structural and lysis genes in this strain results in failure to complete the infection cycle, thereby conferring resistance to the phage.

## 4. Discussion

*S. aureus* MRSA and other antibiotic-resistant pathogens represent an increasing threat to the treatment of bacterial infectious disease, and phage therapy offers a possible anti-bacterial strategy. However, it is vital to have a clear understanding of the phage infection process and the expression of both the host and phage genes. Research into phage transcription and phage-encoded transcriptional regulators over decades has contributed greatly to what we know about prokaryotic transcription in general and the mechanisms by which it can be regulated. However, most of these types of mechanistic studies have focused on highly studied model organisms, such as *E. coli* and *B. subtilis*. Phages of other, more medically relevant bacteria have been much less well-studied in this regard. Here we have investigated transcriptomic details of the phage K infection of the *S. aureus* MRSA strain NRS384. For NRS384_WT_, our findings are generally consistent with previous reports of K-like phage gene transcription following the infection of ST8 strains of *S. aureus*. For example, using RNA-seq data obtained from three timepoints, Kornienko et al. grouped the 238 ORFs of phage vB_SauM-515A1 into 35 transcriptional units (TUs). The TUs were further classified into early or late temporal expression classes, and consensus sequences for 58 early and 12 later promoters were proposed [34]. Because they examined more timepoints after infection (0, 2, 5, 10, 20, and 30 min), Finstrlova et al. were able to categorize the 237 ORFs in phage K into three temporal expression classes: early, middle, and late [36]. Both Kornienko et al. and Finstrlova et al. also presented evidence of transcription from non-coding regions in the phage, hypothesized by them to play roles in the regulation of phage and host gene expression.

Some noteworthy differences between those studies and ours include the timepoints analyzed and the temperature at which infection was performed. The timepoints reported by Kornienko et al. as 5, 15, and 30 min after infection refer to times beginning after a 7 min incubation with the phage and a centrifugation step to remove unadsorbed phages. We therefore estimate that that these timepoints would correspond more closely to timepoints of 15, 25, and 40 min after infection if measured from the time of addition of the phage, as in our study. This may explain why the Kornienko study grouped phage genes into only two temporal expression classes, having perhaps not observed transcriptional activity that occurred immediately after infection. In addition, the Kornienko and Finstrlova studies were based on the phage infection of *S. aureus* SA515, SH1000, or Newman at 37 °C, whereas our infections were conducted at 30 °C. This was necessary because our study used a USA300 MRSA strain; we have observed that *Kayvirus* phages experience growth restriction on USA300 strains at 37 °C [39]. However, the most notable difference between our study and previous studies is the delineation of the effects on the phage transcriptional program of a mutation in the bacterial host RNAP that confers phage K resistance.

Most phages rely wholly or in part on the host machinery, including host RNAP, to complete their developmental program and produce progeny phage. Various phage mechanisms have evolved to take over the host by redirecting host RNAP to preferentially transcribe the phage’s own genes over those of the host and, at the same time, to express those genes in a developmentally appropriate manner. Transcriptional co-option typically involves the interaction of phage proteins with one or more subunits of the RNAP enzyme. One well-studied example is the regulation of middle genes of phage T4 by AsiA/MotA [63]. During infection, the T4 early protein AsiA binds to the σ70 factor of *E. coli* RNAP. In so doing, it remodels σ region 4 into a structure that no longer interacts with host promoter −35 regions, thus suppressing host gene transcription. However, the remodeled structure can be recognized and bound by MotA, thereby redirecting RNAP to phage promoters that contain an upstream −30 motif that is bound by MotA. This redirection leads to the activation of T4 middle promoters, whose expressed gene products include two T4 proteins (gp33, gp55) that replace σ70 and become a sigma factor for the transcription of late genes with the aid of the T4 enhancer-type activator gp45 [64]. Consequently, through this developmental pattern, T4 expresses early, middle, and late genes [65,66], similar to what we have seen for phage K.

For the family of phages that includes bacteriophage K, one example of a well-understood mechanism involved in host takeover is that mediated by Gp67 of *S. aureus* phage G1. This protein binds specifically to the host σA factor of RNAP [67]. Although it binds to the C-terminal region 4 of σA, which is known to interact directly with the −35 region of σA-dependent promoters, it does not appear to inhibit this activity of σ, but acts instead by preventing the C-terminal domain of the α subunit of RNAP from interacting with upstream promoter elements known as UP elements. This blocks transcription from a subset of promoters, including rRNA promoters, that rely on UP elements for full activity [68].

Our study has revealed the probable existence of another *S. aureus* phage-encoded transcriptional regulatory mechanism that redirects the host RNAP to transcribe, late in infection and at high levels, phage structural genes, lysis genes, and other genes whose expression is necessary for the completion of the developmental cycle and release of progeny virions. Although we have yet to identify the relevant phage-encoded product(s), the nature of their interaction with late promoters and/or RNAP, or the late promoters themselves, we have discovered that certain mutations in the *S. aureus rpoC* gene confer resistance to bacteriophage K by preventing late gene expression. The fact that the seven *rpoC* mutations we identified affected only three residues of the b’ subunit suggests that, although our mutational analysis was not saturating, there is probably a fairly small subset of mutations that affect phage gene transcription without dramatically interfering with essential host transcription. An obvious hypothesis for how these mutations cause phage resistance is that they reduce or eliminate the binding of a phage-encoded factor necessary for phage gene transcription. This hypothesis is consistent with the locations of corresponding residues in the structure of *E. coli* RNAP (PDB 6CA0, Figure 2A). In that structure, G36 (corresponding to NRS384 G17) and G89 (corresponding to NRS384 G70) are immediately adjacent to and within, respectively, the zinc-binding domain (ZBD) of the β’ subunit. The ZBD is an essential domain that has been associated with multiple functions. These include binding of σ factors, providing a site for transcriptional regulators of transcription initiation, transcription termination, RNAP recycling, and stretching to interact with the promoter spacer region, which in turn facilitates recognition of promoters that have weak interactions between σ region 4 and the −35 region [69]. A287, corresponding to NRS384 A267, is located within the β’ coiled-coil domain. This domain has been shown to provide a major binding site for the RNAP core/s interaction [70,71]. Thus, all three *rpoC* mutations that we isolated could plausibly affect specific interactions with phage-encoded sigma factors, transcriptional regulators, and/or phage promoters. Further genetic and biochemical analysis will be needed to address if and how these regions of the NRS384 β’ dictate interaction with phage regulators of transcription and to identify those factors. Similarly, additional studies will be needed to identify late gene promoters that respond to such modifications. The presence of such promoters can be inferred from transcriptional analyses such as those reported here. However, for many late genes, clear sequence signatures associated with promoter function are lacking or are partially present and therefore potentially misleading. Functional studies to define transcriptional start sites for late genes, together with more detailed genetic analyses of candidate promoter regions, will be required for a more complete understanding of late gene transcriptional control.

Our results align with previous studies of the transcriptional dynamics of phage K infection and also provide a direct comparison between a sensitive and a resistant host. This work has identified *S. aureus rpoC* G17, and potentially other residues as well, as key amino acids involved in molecular interactions with phage-encoded factors that drive the takeover and redirection of host transcription machinery. It is our hope that this knowledge can be used to identify mechanisms by which *S. aureus* can become resistant to phages that are frequently used for phage therapy, such as those of the *Kayvirus* genus. Doing so may also facilitate the selection or engineering of phages that subvert those mechanisms in order to develop phages and phage cocktails with improved characteristics for phage therapy.

## Figures and Tables

**Table 1:** Mutations in spontaneous NRS384 mutants resistant to phage K.

Mutant	Affected Gene	Mutation	AA Substitution
MR206	*rpoC* (RNA polymerase subunit β’)	G589861A	*rpoC*_G17D_
MR209	*rpoC* (RNA polymerase subunit β’)	G589861A	*rpoC*_G17D_
MR211	*rpoC* (RNA polymerase subunit β’)	G589860C	*rpoC*_G17R_
MR210	*rpoC* (RNA polymerase subunit β’)	G589861T	*rpoC*_G17V_
	capsular polysaccharide biosynthesis protein	T185367A	USA300HOU_0173_383E_
	*ilvB* (acetolactate synthase large subunit)	G2168114A	*ilvB*_A297T_
	ACT domain-containing protein	G2169031T	USA300HOU_2050_V13F_
MR203	*rpoC* (RNA polymerase subunit β’)	G590020A	*rpoC*_G70E_
MR202	*rpoC* (RNA polymerase subunit β’)	C590611A	*rpoC*_A267E_
MR205	*rpoC* (RNA polymerase subunit β’)	C590611A	*rpoC*_A267E_
MR214	tagX* (glycosyl transferase)	T714214A	*tagX*_S165R_

**Table 2:** Co-expressed gene clusters found through Clust analysis.

Cluster	Genes Co-Expressed in the Cluster
C1 (21)	gp010, gp011, gp014, gp015, gp016, gp017, gp018, gp019, gp020, gp021, gp033, gp034, gp035, gp036, gp037, gp038, gp039, gp040, gp042, gp043, gp044
C2 (30)	gp001, gp002, gp003, gp004, gp030, gp050, gp051, gp052, gp053, gp055, gp058, gp059, gp066, gp069, gp076, gp077, gp084, gp086, gp087, gp089, gp090, gp092, gp095, gp139, gp140, gp219, gp220, gp221, gp229, gp230
C3 (25)	gp007, gp008, gp046, gp047, gp064, gp065, gp068, gp071, gp082, gp101, gp112, gp120, gp121, gp125, gp136, gp141, gp197, gp204, gp205, gp206, gp208, gp224, gp225, gp231, gt001
C4 (33)	gp031, gp032, gp100, gp114, gp115, gp116, gp126, gp153, gp154, gp158, gp159, gp160, gp165, gp166, gp167, gp168, gp169, gp170, gp171, gp172, gp174, gp176, gp177, gp178, gp185, gp187, gp188, gp189, gp190, gp193, gp195, gp226, gp227

## Data Availability

The original RNA-Seq data were deposited in GEO under accession GSE253516. The deposited data are publicly available as of the date of publication.

## Supplementary Materials

The following supporting information can be downloaded at: https://www.mdpi.com/article/10.3390/v16111773/s1, Table S1: Information about the identified promoters and terminators in phage K genome and designated TUs. Table S2: DGE data from comparison between consecutive timepoints in phage K infection of NRS384_WT_ host (only genes showing at least a 10% change in expression levels) Table S3: DGE data from comparison between phage K infection of NRS384_WT_ and *rpoC*_G17D_ hosts at each sampled timepoint. Figure S1: One-step growth curve of phage K infecting NRS384_WT_ and *rpoC*_G17D_ hosts. Figure S2: Sequence analysis of the lncRNA regions in *Kayviruses*.
