# Supplementary material for: The Transcriptional Program of Staphylococcus aureus Phage K Is Affected by a Host rpoC Mutation That Confers Phage K Resistance
Source: Viruses. 2024 Nov 13;16(11):1773. doi: 10.3390/v16111773 (PMC11598898; doi:10.3390/v16111773)
Supplement: Supplementary file 1 [file viruses-16-01773-s001.zip › Supplementary Figures.pdf]

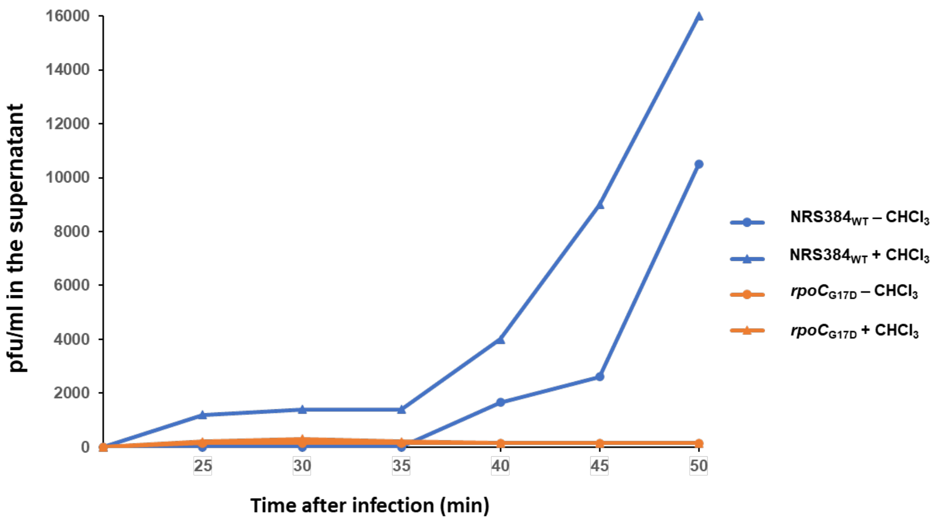

Figure S1: One-step growth curve of phage K infecting $NRS384_{WT}$ and *rpoC*$_{G17D}$. Cultures of $NRS384_{WT}$ and *rpoC*$_{G17D}$ grown to an OD_{600} of ~ 0.3 at 30° C were infected with phage K at an MOI of 0.01. Unadsorbed phage were removed by centrifugation, and the bacteria were resuspended in fresh media. Samples were collected at the noted timepoints to calculate the number of phages assembled within the infected cells (with $CHCl_3$) and released into the supernatant after lysis. (without $CHCl_3$)

a

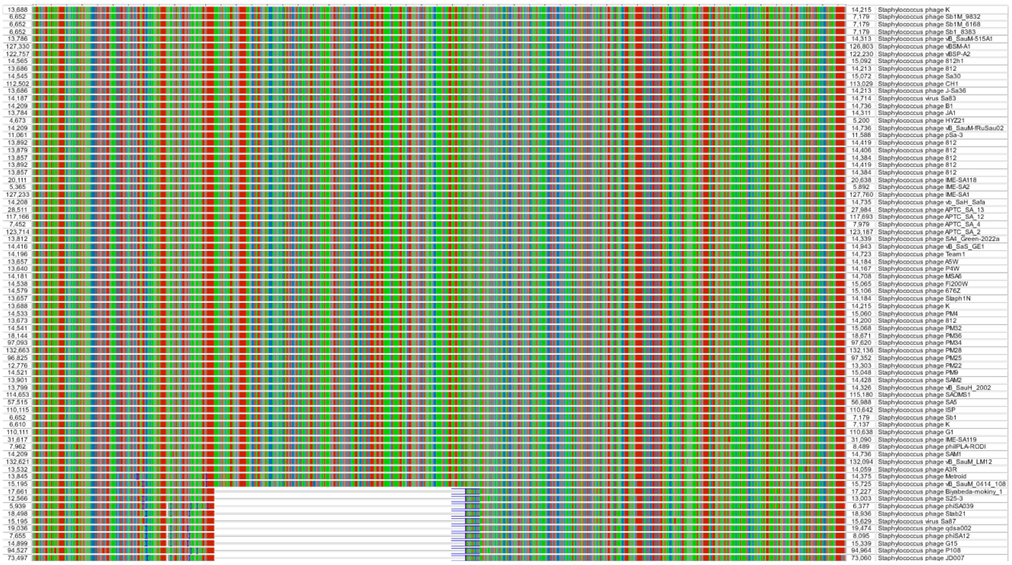

b

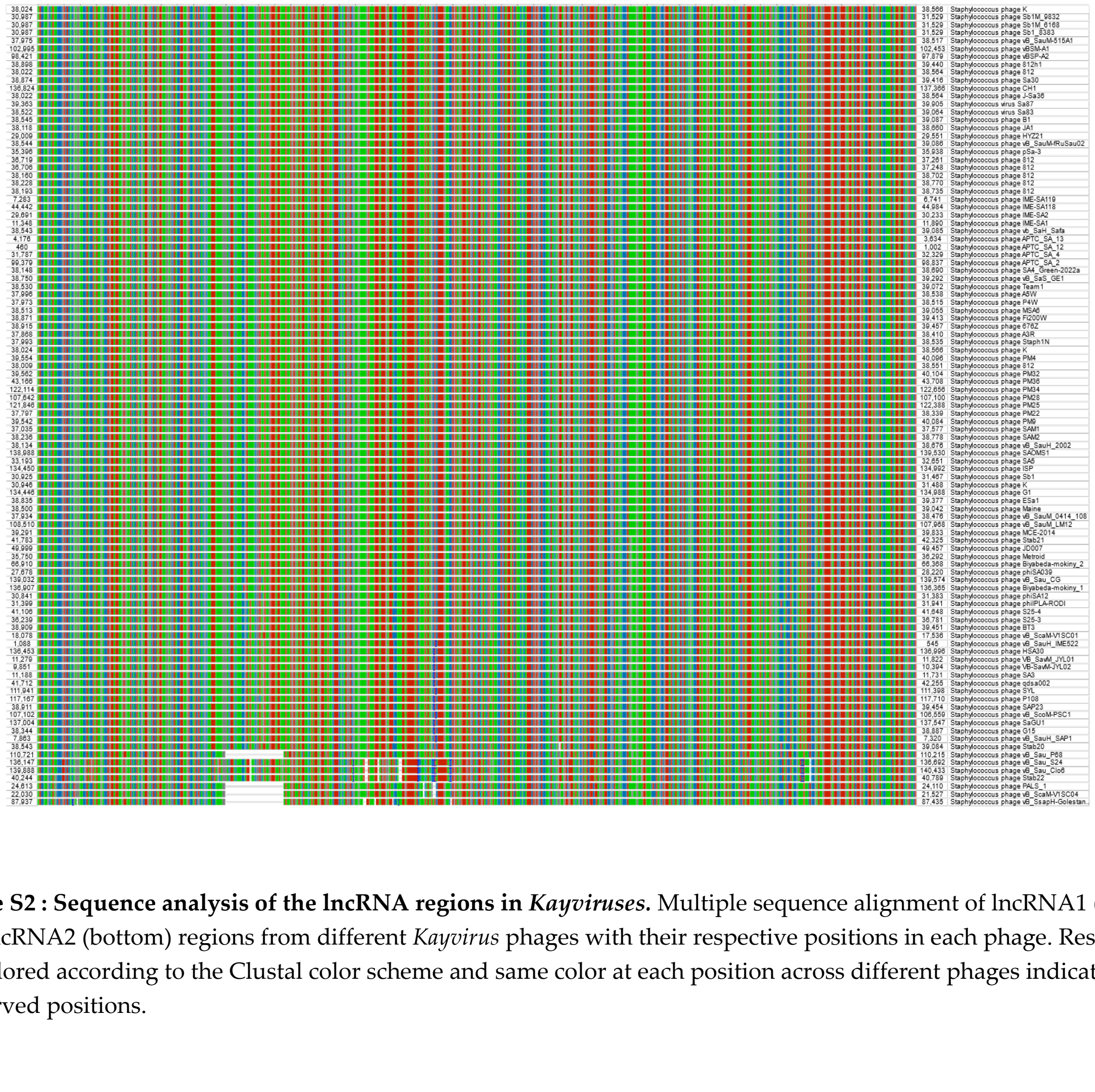

Figure S2 : Sequence analysis of the lncRNA regions in *Kayviruses*. Multiple sequence alignment of lncRNA1 (top) and lncRNA2 (bottom) regions from different *Kayvirus* phages with their respective positions in each phage. Residues are colored according to the Clustal color scheme and same color at each position across different phages indicates conserved positions.
