# Supplementary material for: The Transcriptional Program of Staphylococcus aureus Phage K Is Affected by a Host rpoC Mutation That Confers Phage K Resistance
Source: Viruses. 2024 Nov 13;16(11):1773. doi: 10.3390/v16111773 (PMC11598898; doi:10.3390/v16111773)
Supplement: Supplementary file 1 [file viruses-16-01773-s001.zip › Table S1.pdf]

								-35				-10			
Genes	TU#	genes in TU	Cluster	Classification	Promoter sequence		Coord.	total	cons.	spacing	ext. -10	total	cons.	Method of detection	Terminator*
gp001			2		TTGACAACTATGAAGCGGGTATGCTATAAT	↓	425-454	consensus		18	✓	consensus		👁💻	
gp002			2												
gp003			2												
gp004		8	2	Middle											
gp005															
gp006															
gp007			3												
gp008	1		3												3125-3154
gp009	2	1		Middle	TTGACTTCTGAATAACTATACTGTAATAT	↑	3586-3614	5/6	2/2	17		4/6	3/3	👁💻	3117-3146
gp010			1		TTGACTTTATTATCATATGGTAGTAATAT	↓	3709-3737	5/6	2/2	17		4/6	3/3	👁💻	
gp011		3	1	Early	TTGACACCTTACAAGATACATGTTATTAT	↓	4219-4247	consensus		17	✓	5/6	3/3	👁💻	
gp012	3														4875-4904
gp013	4	1		Early	TTGACATTAAGACCGAATTATTATATAAT	↑	5691-5719	consensus		17		consensus		👁💻	5142-5168
gp014			1		TTGACTTTAATATCATTATAGTTTAATAT	↓	5765-5793	5/6	2/2	17		4/6	3/3	👁💻	
gp015			1		TTGACAACCTAGAAACAACATGTTAATAT	↓	5988-6016	consensus		17	✓	4/6	3/3	👁💻	
gp016			1		TTGACAACTTAAACACTACATGTTATTAT	↓	6189-6217	consensus		17	✓	5/6	3/3	👁	
					TTGACAGTCACTTGAAACCATGATATTAT	↓	6285-6313	consensus		17	✓	5/6	3/3	👁💻	
gp017		8	1	Early	TTGACTTTCAAGCCCTACCATGTTATTAT	↓	6690-6718	5/6	2/2	17	✓	5/6	3/3	👁💻	
gp018			1		TTGACATCCTAACATATAGATGGTAATAT	↓	7021-7049	consensus		17	✓	4/6	3/3	👁💻	
gp019			1												
gp020			1												
gp021	5		1		TTTACAATCTTTTAGTTTGTATGATATAAT	↓	8082-8111	5/6	1/2	18	✓	consensus		👁💻	8370-8395
gp022															
gp023															
gp024															
gp025															
gp026		9		Middle	TTGATAAAGAATGGAAAAAATTATTAATAT	↑	10452-10481	5/6	2/2	18		4/6	3/3	💻	
gp027															
gp028					TGGATGATTTTGATATGGATAAATATGAT	↑	11574-11602	3/6	1/2	17		5/6	3/3	👁	
gp029															
gp030	6		2		TTGACTTTTTTTACTAAGTATGGTAAGAT	↑	12635-12663	5/6	2/2	17	✓	4/6	3/3	👁💻	
gp031			4		GAAACAGAACCAAGTGATAAGAGTAAATT	↑	13299-13327	3/6	1/2	17		4/6	3/3	👁	
		2		Late	TGTACAAAATATAGTAAAGGAGATAAAAT	↑	13597-13625	4/6	1/2	17		5/6	3/3	👁	
gp032	7		4		TAAACTAATAGGTGTTTTTTTGTTATATT	↑	13629-13527	3/6	1/2	17	✓	5/6	3/3	👁	12663-12692
tRNA and lncRNA	8	IA and 1 lncRNA		Late	TTGATAAATTAACTTGACATTAAGTATAAT	↑	14567-14596	5/6	1/2	18		consensus		💻	
gp233															
gp232		3		Middle	TAGATATGATGAATATTATTGTAGTAGAAT	↑	15416-15445	4/6	1/2	18		5/6	3/3	💻	
gp231	9		3		ATAACAAATAAAAGGAGGATGTTATAAT	↑	16274-16298	3/6	1/2	16	✓	consensus		👁	
gp230			2		TTGACATTATTATCAATATATGTTATTAT	↑	16303-16331	consensus		16	✓	5/6	3/3	👁	
gp229		3	2	Middle											
gp228	10				TTGACAAAATATAAAAAAATAGTGTATAGT	↑	17113-17141	consensus		17		5/6	3/3	👁💻	13637-13665
gp227		2	4	Late	ATGACTTAGAAAAAGACCTATGATATATT	↑	17388-17416	4/6	2/2	17		5/6	3/3	💻	
gp226	11		4		no promoter										17143-17169
gp225			3												
gp224			3		TTGACTTTTTCACTAACTTATGTTATACT	↑	19095-19123	5/6	2/2	17		5/6	3/3	💻	
gp223															
gp222		7		Middle											
gp221			2												
gp220			2												
gp219	12		2		TTGACAAATACAAATACTTGTAATATAAT	↑	21456-21484	consensus		17		consensus		👁💻	17909-17937
gp218		2		Middle	TTGATTCTATGATATTTAATATTGTAAAAT	↑	22008-22037	4/6	1/2	18		5/6	3/3	💻	
gp217	13				no promoter										21519-21557
gp216															
gp215		4			TTGACAATAGTATCATAATATGATATAAT	↑	22950-22978	consensus		17	✓	consensus		💻	
gp214															
gp213	14			Middle	TTGACAAATATTATTTACTATGGTATGAT	↑	25219-25247	consensus		17	✓	5/6	3/3	👁💻	22335-22368
gp212															
gp211		3													
gp210	15			Late	TAGACAAGGACATGGTAAACCATACAAT	↑	26432-26459	5/6	2/2	16		5/6	3/3	💻	
gp209															
gp208		3	3												
gp207	16			Middle	TTGACAAATCCCCTTAGTTATGGTATAAT	↑	28067-28095	consensus		17	✓	consensus		👁💻	25249-25277
gp206			3		TTGATATATAAAAAGGAGAAAATATTAT	↑	29510-29537	5/6	2/2	16		5/6	3/3	💻	
gp205			3												
gp204			3		TTGAGTTAGTTATTAATTTAAAATAAAAT	↑	30153-30181	4/6	1/2	17		5/6	3/3	💻	
gp203		7													
gp202															
gp201															
gp200	17			Middle	TTGACTTCATAAGTTAACTATGCTATAAT	↑	31280-31308	5/6	2/2	17	✓	consensus		👁💻	28037-28086
gp199	18	1		Middle	TTGCGTTATTTAAAGATATATGTTATGAT	↑	32109-32137	3/5	1/2	17	✓	5/6	3/3	💻	
					AATAATATTACACTAATTAGTGCTATATT	↑	32141-32170	1/6	0/2	n/a?	✓	5/6	3/3	👁	31320-31348
gp198	19	1		Middle	TTGACATAGGTGGTTTTTTATGCTATAGT	↑	32893-32921	consensus		17	✓	5/6	3/3	👁💻	32214-32244
gp197		2	3												
gp196	20			Middle	TTGACAAAATTAAATACATAGTGTATAGT	↑	34069-34097	consensus		17		5/6	3/3	👁💻	32901-32928
gp195		3*	4												
gp193	21		4	Late	GATAAACATGACCGACCTACTGTTATATT	↑	37056-37084	2/6	0/2	n/a?	✓	5/6	3/3	👁	
gp192															
gt002															
gt003		tRNA + 1 ln													
gt004															
lnc RNA	22			Late	TTGACAAGTAATAAAAATTATGTTATAAT	↑	38602-38630	consensus		17	✓	consensus		👁💻	34111-34137
gp191	23	1		Late	TTGACTTATTTATCAATATAGTATATAGT	↑	39090-39118	5/6	2/2	17		5/6	3/3	💻	38644-38675
gp190			4												
gp189		4	4												
gp188			4												
gp187	24		4	Late	TTGACTTTTATGACTGGCTTTTAATGTTATATT	↑	40780-40812	5/6	2/2	21	✓	5/6	3/3	💻	39496-39521
gp186					TTTAAATTTACTTATTTTGTGGTATAAT	↓	40448-40475	4/6	0/2	16	✓	consensus		💻	
					TTGACCTATTATTTCTAGAACTTTTAGATT	↓	40742-40771	5/6	2/2	18		4/6	3/3	👁	
gp185			4												
gp184															
gp183	25	8		Late	TTGAGTGCAGAAAATATTAGAGATATAAT	↓	41501-41529	4/6	1/2	17		consensus		💻	
gp182															
gp181					TTGACAGATAAATTATTTTATGGTACAAT	↓	43757-43785	consensus		17	✓	5/6	3/3	💻	
gp180															
gp179															46420-46443
gp178			4		AATTACTCCTCTTTTTTGTTTGCTATATT	↓	46427-46455	0/6	0/2	NA	✓	5/6	3/3	👁	
	26	3			TTGCTATATTAAATAAGAGCTAAATATAAA	↓	46446-46475	4/6	1/2	18		5/6	2/3		
gp177			4												
gp176			4	Late											48946-48973
gp175					TTGATAAATGTAATAACTATGATATACT	↓	48992-49019	5/6	1/2	16	✓	5/6	3/3	👁	
					TTGATATTAATACATAAAAAATATTAATAT	↓	49028-49057	5/6	1/2	18		4/6	3/3	👁💻	
					TTCACTTACAAGTTATTATTGTTATATT	↓	49058-49085	4/6	1/2	16	✓	5/6	3/3	👁	
					TTCACTTACAAGTTATTATTGTTATATTAT	↓	49058-49087	4/6	1/2	18		5/6	3/3	💻	
gp174		3	4	Late											
gp173					TTAATTAATGCACAAAGTTGTGTTATATT	↓	50891-50899	2/6	0/2	NA	✓	5/6	3/3	👁	
					GGTACATTTTACTGTACCCTACATAAAAT	↓	50928-50956	3/6	1/2	17		5/6	3/3	👁	
	27				AAAAGAACACAAATGAAAGGTGATAAATT	↓	50958-50986	2/6	0/2	NA	✓	4/6	3/3	👁	52413-52443
gp172			4		no promoters										
gp171			4												

gp170			4												
gp169		7	4												
gp168			4												
gp167			4												
gp166	28		4	Late											58075-58106
gp165	29	1	4	Late	no promoters*										58573-58599
gp164					TTGACACTTTAAAATTTATATGTTATTAT	↓	58601-58629	consensus		17	✓	5/6	3/3	👁💻	
gp163					TTGACAATTATAATTAACTAAGGTATATT	↓	58786-58814	consensus		17		5/6	3/3	👁	
gp162		4													
gp161					TTGACAATTCAATAAGGAGGTATTATAAT	↓	59289-59317	consensus		17		consensus		💻	
	30			Middle	TTGACAAATTAAAACTAATAAATTATAAT	↓	59530-59558	consensus		17		consensus		👁💻	
gp160		2	4		TTGATTTAAAAACAAAATATGATATACT	↓	59906-59933	4/6	1/2	17	✓	5/6	3/3	👁💻	
gp159	31		4	Late											61075-61106
gp158	32	1	4	Late	no promoter for gp158										
gp157					TTGACACAAGAGTAGTATCATAGTATACT	↓	65195-65223	consensus		17		5/6	3/3	👁💻	
gp156		3													
gp155	33			Middle	TTTACAGAGGGAAGATTCTTTACTATATT	↓	68330-68358	5/6	1/2	17		5/6	3/3	💻	71144-71168
gp154			4		TTGTAATTTAAACTAGTTCGTGATATATT	↓	71171-71199	4/6	1/2	17	✓	5/6	3/3	👁💻	
gp153			4												
gp152															
gp151		7		Middle											
gp150					TTGATAACCTAGATGAGAACATTATAGT	↓	74244-74271	5/6	1/2	16		5/6	3/3	💻	
gp149					TTGACAGAAAGTTAATAATATGGTATACT	↓	77412-77440	consensus		17	✓	5/6	3/3	💻	
					AATATTTAGTGGGTATACCATGTTATATT	↓	77469-77477	1/6	0/2	NA	✓	5/6	3/3	👁	
gp148	34														81519-84545
gp147					no promoters										
gp146		4		Middle											
gp145															
gp144	35														85424-85454
gp143					TTGACTTGAAAAGGATTCTGTGGTATACT	↓	85456-85484	5/6	2/2	17	✓	5/6	3/3	👁💻	
gp142		3		Middle	TTGATTTTAATGATATGACAAATAGATT	↓	87161-87188	4/6	1/2	16		4/6	3/3	💻	
gp141	36		3		TTGACAAAAGAGGGTATGTTGGATTATAAT	↓	88815-88844	consensus		18		consensus		💻	90339-90374
gp140			2		TTGACATTTTATATGTTAGGTGGTATAAT	↓	90343-90371	consensus		17	✓	consensus		👁💻	
gp139			2												
gp138															
gp137															
gp136			3												
gp135					TTGACCTTAGAGAAGTTTTATGTTATACT	↓	95417-95445	5/6	2/2	17	✓	5/6	3/3	👁	
gp134		13		Middle											
gp133															
gp132					TTGACCTCATTTACACCTTCTTATATTAT	↓	96726-96754	5/6	2/2	17		5/6	3/3		
gp131															
gp130															
gp129															
gp128	37														101129-101176
gp127		2		Late	TTGGAATTTCCTACTATCTGTGCTATACT	↓	101120-101148	4/6	1/2	17	✓	5/6	3/3		
gp126	38		4												102225-102251
gp125			3		TTGACAAGGTTTAAAATATATGGTATAGT	↓	102253-102281	consensus		17	✓	5/6	3/3	👁💻	
gp122		6*			TTGACAATATAGTTAACTTATGTTATACT	↓	107419-107447	consensus		17	✓	5/6	3/3	👁💻	
gp121			3												
gp120	39		3	Middle	TTGACAAATATAAAAAACTATGTTATAAT	↓	108242-108270	consensus		17	✓	consensus		👁💻	109585-109610
gp119					TAGAGCACTCTTTTATTTGAGATATAAT	↓	109593-109620	3/6	1/2	16		consensus			
gp118		3			TTGAGATATAATTACTAGGAGGATATTAA	↓	109609-109637	5/6	1/2	17		4/6	2/3		
gp117	40			Middle											111908-111968
gp116			4		TAGTTACAGTGCCTTCCTTGTGTTATATT	↓	111898-111926	3/6	1/2	17		5/6	3/3		
		3		Late	GTTACAGTGCCTTCCTTGTGTTATATTAT	↓	111900-111928	4/6	1/2	17		5/6	3/3	👁	
gp115			4												
gp114	41		4												113445-113471
gp113					TTGACAATTTATAATATCTATGATACACT	↓	113473-113501	consensus		17	✓	4/6	3/3	👁	
gp112			3												
gp111		5													
gp110															
gp109	42			Middle	TTTATTTACATGCTAGTTTATGTTATAAT	↓	116058-116086	3/6	0/2	NA	✓	consensus			116475-116501
gp108					TTGACTCTTTTTACTATATATGGTATATT	↓	116490-116518	5/6	2/2	17	✓	5/6	3/3	👁💻	
gp107															
gp106					TTTACAAGAGGTGTTATCTATGGTTATAAT	↓	117719-117748	5/6	1/2	18		consensus			
gp105															
					TTGTAAATAGTTAAAAAATATATTAAAAT	↓	119282-119310	4/6	1/2	17		5/6	3/3	👁	
gp104		8			TATACAAAGTAGTTAATAAAATTATATT	↓	119313-119340	4/6	1/2	16		5/6	3/3		
					ATGAAATAATAACAGAAATTGTGATATATT	↓	119354-119383	4/6	1/2	18	✓	5/6	3/3	👁	
gp103															
gp102					TTGACAGCTCCTATAGTTTATGATATAGT	↓	120704-120732	consensus		17	✓	5/6	3/3	👁	
gp101	43		3	Middle	TTGTCGTTGAGTAAGTAAAGAGGTTATAAT	↓	121196-121225	4/6	2/2	18		consensus			121936-121974
gp100	44	1	4	Late	no promoter										122468-122496
gp099					TTGACTCTCTTTTTGTTTTATGGTATATT	↓	122483-122511	5/6	2/2	17	✓	5/6	3/3	👁	
gp098															
gp097					TTGACAAGAACAAATAAGTGTAGTATAGT	↓	123325-123353	consensus		17		5/6	3/3	💻	
gp096															
gp095			2												
gp094															
gp093															
gp092			2		TTGACAGATGAAGCATTTTAATATATACT	↓	124796-124824	consensus		17		5/6	3/3	👁	
gp091															
gp090			2												
gp089		20	2	Middle											
gp088					TTGACACCTTTGTACTTTTGTATTATACT	↓	126075-126103	consensus		17		5/6	3/3	(👁)	
gp087			2												
gp086			2												
gp085															
gp084			2		TTGACAATTGAGTATACATAGGTTATACT	↓	127676-127704	consensus		17		5/6	3/3	👁	
gp083															
gp082			3												
gp081					TAGATATTTAATAAGGAGGAAATTAATAT	↓	128513-128541	4/6	1/2	17		4/6	3/3		
					TTTACATGTAATTAACCCTGAGTATTAT	↓	128767-128794	5/6	1/2	16		5/6	3/3	(👁)	
gp080	45														129218-129249
gp079					TTGACATTAGGTTTCTTTTATTATATACT	↓	129226-129254	consensus		17		5/6	3/3	👁💻	
gp078															
gp077			2												
gp076			2												
gp075															
gp074		10		Middle											
gp073					TTGACAGCTACAATATAGTGTGTTACAGT	↓	131293-131321	consensus		17	✓	4/6	3/3	👁	
gp072															
gp071			3												
gp070	46														132816-132849

gp069			2		TTGACAGCTACAATATAGTGTGTTACAGT	↓	132831-132859	consensus		17		5/6	3/3	👁	
gp068			3												
gp067		6		Middle											
gp066			2												
gp065			3												
gp064	47		3		TTGACAAACTTAAGGTAAGTAGTGTATCAT	↓	134667-134696	consensus		18		5/6	3/3		136378-136406
gp063					TTGACAAAGGGAGTTTTTTATTATATAGT	↓	136385-136413	consensus		17		5/6	3/3	👁💻	
gp062															
gp061															
gp060					TTGACTTAGGTAGATACTTATTATATAAT	↓	137365-137393	5/6	2/2	17		consensus		👁💻	
gp059		10	2	Middle											
gp058			2												
gp057															
gp056															
gp055			2												
gp054	48														term. Rep.
gp053			2		TTGACAACTATGAAGCGGGTATGCTATAAT	↓		consensus		18	✓	consensus		👁💻	
gp052			2												
gp051			2												
gp050		8		Middle											
gp049															
gp048															
gp047			3												
gp046	49		3												142956-142985
gp045	50	1		Middle	TTGACTTCTGAATAACTATACTGTAATAT	↑		5/6	2/2	17		4/6	3/3	👁💻	142948-142977
gp044			1		TTGACTTTATTATCATATGGTAGTAATAT	↓		5/6	2/2	17		4/6	3/3	👁💻	
gp043		3	1	Early	TTGACACCTTACAAGATACATGTTATTAT	↓		consensus		17	✓	5/6	3/3	👁💻	
gp042	51		1												144706..144735
gp041	52	1		Early	TTGACATTAAGACCGAATTATTATATAAT	↑		consensus		17		consensus		👁💻	144973..144999
gp040			1		TTGACTTTAATATCATTATAGTTTAATAT	↓		5/6	2/2	17		4/6	3/3	👁💻	
gp039			1		TTGACAACCTAGAAACAACATGTTAATAT	↓		consensus		17	✓	4/6	3/3	👁💻	
					TTGACAACTTAAACACTACATGTTATTAT	↓		consensus		17	✓	5/6	3/3	👁	
gp038			1		TTGACAGTCACTTGAAACCATGATATTAT	↓		consensus		17	✓	5/6	3/3	👁💻	
gp037		8	1	Early	TTGACTTTCAAGCCCTACCATGTTATTAT	↓		5/6	2/2	17	✓	5/6	3/3	👁💻	
gp036			1		TTGACATCCTAACATATAGATGGTAATAT	↓		consensus		17	✓	4/6	3/3	👁💻	
gp035			1												
gp034			1												
gp033	53		1		TTTACAATCTTTTAGTTTGTATGATATAAT	↓		5/6	1/2	18	✓	consensus		👁💻	148201..148218
