# Supplementary material for: The Transcriptional Program of Staphylococcus aureus Phage K Is Affected by a Host rpoC Mutation That Confers Phage K Resistance
Source: Viruses. 2024 Nov 13;16(11):1773. doi: 10.3390/v16111773 (PMC11598898; doi:10.3390/v16111773)
Supplement: Supplementary file 1 [file viruses-16-01773-s001.zip › Table S2.pdf]

Table S2A: These Log2FC values were calculated comparing the counts from 4 replicates of RNA samples collected from phage K infections of NRS384WT at 0 and 2 min. Only genes showing a statistically significant change in expression (adj.p.value <=0.05) are shown.

**geneID**	**log2FC**	**AveExpr**	**t**	**P.Value**	**adj.P.Val**	**B**	
CPT_phageK_gt002	-1.16453	7.303121	-2.77226	0.015987	0.033258	-3.62859	
CPT_phageK_gp233	0.465657	10.155	2.878639	0.013051	0.028342	-3.9501	
CPT_phageK_gp228	0.665985	10.29886	3.695886	0.002739	0.008702	-2.40564	
CPT_phageK_gp221	0.700632	12.71713	6.639496	1.71E-05	9.46E-05	2.546599	
CPT_phageK_gp220	0.881138	13.15221	6.603372	1.80E-05	9.76E-05	2.460417	
CPT_phageK_gp214	0.903859	7.147449	2.719795	0.017665	0.035482	-3.57403	
CPT_phageK_gp213	0.638699	13.36592	5.891374	5.56E-05	2.49E-04	1.246613	
CPT_phageK_gp207	-0.82948	7.064249	-2.51184	0.026176	0.049575	-4.03811	
CPT_phageK_gp202	0.70683	11.4811	3.380173	0.004997	0.013082	-3.20004	
CPT_phageK_gp197	0.333729	12.99945	3.398604	0.004824	0.012773	-3.33537	
CPT_phageK_gp195	-0.79036	14.23429	-7.13432	8.14E-06	5.02E-05	3.083661	
CPT_phageK_gp193	-1.44254	11.01404	-7.27378	6.64E-06	4.42E-05	3.636424	
CPT_phageK_gp192	-0.92822	7.855722	-3.12693	0.008115	0.019098	-3.06417	
CPT_phageK_gp190	-1.78174	5.525292	-4.54422	5.66E-04	0.00203	-0.09361	
CPT_phageK_gp189	-1.55625	8.233132	-6.3004	2.88E-05	1.43E-04	2.532092	
CPT_phageK_gp188	-1.51948	8.005969	-5.89999	5.48E-05	2.49E-04	1.916334	
CPT_phageK_gp186	-1.09421	9.276085	-4.74723	3.93E-04	0.001453	-0.32189	
CPT_phageK_gp185	-1.45415	8.830485	-8.29319	1.62E-06	1.52E-05	5.400076	
CPT_phageK_gp184	-1.24279	9.754515	-7.76171	3.33E-06	2.60E-05	4.537581	
CPT_phageK_gp183	-1.38997	13.06344	-13.3579	6.52E-09	1.64E-07	10.68007	
CPT_phageK_gp182	-1.06034	10.52774	-6.16114	3.60E-05	1.75E-04	1.948644	
CPT_phageK_gp180	-1.16156	12.00535	-7.90302	2.74E-06	2.46E-05	4.446088	
CPT_phageK_gp179	-1.49844	13.71136	-11.001	6.55E-08	1.02E-06	8.182436	
CPT_phageK_gp178	-2.23653	7.327853	-7.82399	3.06E-06	2.60E-05	4.912941	
CPT_phageK_gp177	-2.04657	8.94464	-12.0537	2.23E-08	4.00E-07	9.756012	
CPT_phageK_gp176	-1.68709	12.25356	-15.3115	1.24E-09	4.14E-08	12.48201	
CPT_phageK_gp175	-1.73277	13.00081	-12.4681	1.49E-08	3.16E-07	9.798124	
CPT_phageK_gp174	-1.67109	13.58045	-12.3348	1.69E-08	3.29E-07	9.614404	
CPT_phageK_gp173	-1.79907	16.01134	-10.4872	1.14E-07	1.57E-06	7.579683	
CPT_phageK_gp172	-1.03546	8.202645	-5.00823	2.48E-04	9.78E-04	0.369144	
CPT_phageK_gp171	-1.4923	11.92652	-10.1778	1.62E-07	2.09E-06	7.423356	
CPT_phageK_gp170	-1.31649	11.44822	-8.63862	1.04E-06	1.10E-05	5.532493	
CPT_phageK_gp169	-1.22496	10.47928	-7.40952	5.47E-06	3.77E-05	3.921797	
CPT_phageK_gp168	-1.28357	12.30362	-8.42316	1.37E-06	1.39E-05	5.14125	
CPT_phageK_gp167	-1.68546	9.251282	-7.20439	7.35E-06	4.76E-05	3.771721	
CPT_phageK_gp166	-1.59892	15.15532	-11.2099	5.26E-08	8.75E-07	8.395769	
CPT_phageK_gp165	-1.39388	11.97757	-7.7155	3.55E-06	2.60E-05	4.169745	
CPT_phageK_gp163	0.702529	10.82847	3.367816	0.005116	0.013246	-3.13286	
CPT_phageK_gp161	0.928657	11.04592	3.63494	0.003074	0.009303	-2.63898	
CPT_phageK_gp160	-1.03856	9.930942	-5.65731	8.17E-05	3.46E-04	1.191446	
CPT_phageK_gp159	-1.13953	10.73732	-9.12126	5.64E-07	6.92E-06	6.260513	
CPT_phageK_gp158	-1.11945	12.69063	-10.6872	9.18E-08	1.34E-06	7.953268	

CPT_phageK_gp156	0.518929	11.07229	3.63766	0.003059	0.009303	-2.66316	
CPT_phageK_gp151	0.303716	12.296	2.507646	0.026383	0.049575	-4.94564	
CPT_phageK_gp149	-0.47542	12.62564	-4.83577	3.36E-04	0.001282	-0.61418	
CPT_phageK_gp148	-0.52936	16.11945	-4.69293	4.33E-04	0.001577	-1.09421	
CPT_phageK_gp146	-0.84035	13.72737	-8.28961	1.63E-06	1.52E-05	4.814619	
CPT_phageK_gp145	-1.32331	9.214365	-7.40562	5.50E-06	3.77E-05	4.090721	
CPT_phageK_gp144	-0.97881	13.55874	-7.78406	3.23E-06	2.60E-05	4.101351	
CPT_phageK_gp143	-0.27295	13.59405	-2.5568	0.024051	0.045934	-5.0429	
CPT_phageK_gp141	0.240258	13.66983	2.581947	0.022937	0.044167	-4.96601	
CPT_phageK_gp138	0.296735	13.95287	3.317545	0.005633	0.014266	-3.60956	
CPT_phageK_gp137	0.398028	11.74763	3.209091	0.006933	0.016827	-3.57552	
CPT_phageK_gp130	-0.75778	13.11504	-5.55605	9.67E-05	4.03E-04	0.60014	
CPT_phageK_gp129	-0.50576	9.141057	-2.63383	0.020794	0.041058	-4.23897	
CPT_phageK_gp128	-0.72372	10.48352	-3.43379	0.00451	0.012079	-3.00921	
CPT_phageK_gp127	-0.69592	11.31915	-5.21463	1.73E-04	6.95E-04	0.204236	
CPT_phageK_gp125	0.310089	15.45502	3.691072	0.002764	0.008702	-3.0006	
CPT_phageK_gp124	-5.23442	4.971493	-3.30522	0.005767	0.014449	-2.27828	
CPT_phageK_gp121	0.47536	12.05621	4.025917	0.001471	0.004967	-2.03348	
CPT_phageK_gp116	-0.97519	11.01732	-5.45134	1.15E-04	4.72E-04	0.660014	
CPT_phageK_gp115	-1.2702	11.72641	-6.83223	1.27E-05	7.24E-05	2.863671	
CPT_phageK_gp114	-1.04875	6.831071	-2.67567	0.019208	0.038253	-3.71026	
CPT_phageK_gp108	0.428887	10.64922	3.039308	0.009598	0.021711	-3.74561	
CPT_phageK_gp102	0.623214	10.68618	3.946396	0.001707	0.005683	-2.00445	
CPT_phageK_gp101	0.253437	13.31929	2.586691	0.022732	0.044138	-4.91066	
CPT_phageK_gp093	0.974631	9.257323	3.496024	0.004005	0.011384	-2.50752	
CPT_phageK_gp091	0.852024	9.99478	3.534507	0.003722	0.010977	-2.63148	
CPT_phageK_gp090	0.426956	10.95452	2.913974	0.012199	0.026814	-4.03266	
CPT_phageK_gp088	1.196124	8.571235	3.020516	0.009949	0.02229	-3.23728	
CPT_phageK_gp087	0.833393	9.472191	2.864256	0.013414	0.028675	-3.77159	
CPT_phageK_gp086	0.39236	12.84062	2.875192	0.013137	0.028342	-4.31408	
CPT_phageK_gp085	0.985836	9.678162	3.449461	0.004377	0.011999	-2.70729	
CPT_phageK_gp084	0.424324	12.21211	2.850163	0.01378	0.028926	-4.29969	
CPT_phageK_gp080	0.757255	9.771761	3.437731	0.004477	0.012079	-2.7727	
CPT_phageK_gp079	0.754257	9.95799	2.856017	0.013627	0.028865	-3.92033	
CPT_phageK_gp077	0.928119	7.938965	2.761788	0.016309	0.033627	-3.62183	
CPT_phageK_gp076	0.688025	10.73524	4.868539	3.17E-04	0.00123	-0.28024	
CPT_phageK_gp070	0.707304	11.59873	3.60398	0.003261	0.009741	-2.78382	
CPT_phageK_gp065	0.48221	11.56264	4.036334	0.001443	0.004943	-1.96452	
CPT_phageK_gp063	1.95607	5.910272	3.276332	0.006095	0.015108	-2.33864	
CPT_phageK_gp062	1.184075	8.667958	3.328846	0.005512	0.014114	-2.6759	
CPT_phageK_gp058	0.789079	8.873387	3.099948	0.008545	0.01952	-3.18548	
CPT_phageK_gp057	1.06581	7.602078	2.618215	0.021417	0.041935	-3.82071	
CPT_phageK_gp056	1.819728	5.206784	3.451513	0.00436	0.011999	-1.996	
CPT_phageK_gp055	1.216198	10.71016	7.130713	8.18E-06	5.02E-05	3.558707	
CPT_phageK_gp054	1.019171	10.50403	4.785083	3.67E-04	0.00138	-0.37095	
CPT_phageK_gp053	0.524543	10.34106	3.181086	0.007315	0.017392	-3.41038	
CPT_phageK_gp050	0.541674	9.697903	3.104663	0.008468	0.01952	-3.40563	

CPT_phageK_gp049	1.111627	8.778916	4.338624	8.24E-04	0.002866	-0.80067	
CPT_phageK_gp048	1.187206	7.188452	3.495859	0.004006	0.011384	-2.12474	
CPT_phageK_gp045	-1.00321	8.411111	-2.74667	0.016785	0.034007	-3.90416	
CPT_phageK_gp044	-2.04912	11.65409	-15.4775	1.09E-09	4.14E-08	12.66602	
CPT_phageK_gp043	-1.67412	13.08195	-17.4649	2.46E-10	2.87E-08	14.09658	
CPT_phageK_gp042	-2.08629	8.006872	-8.85329	7.88E-07	8.74E-06	6.217093	
CPT_phageK_gp040	-1.01488	10.14895	-5.69826	7.63E-05	3.29E-04	1.228319	
CPT_phageK_gp039	-1.3558	11.79094	-6.86428	1.21E-05	7.07E-05	2.903616	
CPT_phageK_gp038	-1.81605	13.82145	-16.2375	6.05E-10	3.52E-08	13.10597	
CPT_phageK_gp037	-1.72738	12.38606	-13.2711	7.05E-09	1.64E-07	10.65334	
CPT_phageK_gp036	-1.44855	11.76653	-6.40508	2.45E-05	1.24E-04	2.168178	
CPT_phageK_gp035	-1.01046	8.729578	-3.80734	0.002218	0.007178	-1.96772	
CPT_phageK_gp034	-1.29468	11.6555	-7.71299	3.57E-06	2.60E-05	4.207823	
CPT_phageK_gp033	-1.396	9.60264	-6.09643	3.99E-05	1.86E-04	1.97293	
CPT_phageK_gp032	-0.97702	9.924916	-6.45212	2.28E-05	1.21E-04	2.522367	
CPT_phageK_gp030	0.524017	11.77243	3.672958	0.00286	0.008886	-2.68053	
CPT_phageK_gp029	1.715057	7.192233	2.989561	0.010557	0.023426	-3.03643	
CPT_phageK_gp027	0.599094	9.745967	3.231596	0.00664	0.016287	-3.17503	
CPT_phageK_gp022	-0.85825	7.567358	-3.46182	0.004275	0.011999	-2.36893	
CPT_phageK_gp021	-1.396	9.60264	-6.09643	3.99E-05	1.86E-04	1.97293	
CPT_phageK_gp020	-1.29468	11.6555	-7.71299	3.57E-06	2.60E-05	4.207823	
CPT_phageK_gp019	-1.01046	8.729578	-3.80734	0.002218	0.007178	-1.96772	
CPT_phageK_gp018	-1.44855	11.76653	-6.40508	2.45E-05	1.24E-04	2.168178	
CPT_phageK_gp017	-1.72738	12.38606	-13.2711	7.05E-09	1.64E-07	10.65334	
CPT_phageK_gp016	-1.81605	13.82145	-16.2375	6.05E-10	3.52E-08	13.10597	
CPT_phageK_gp015	-1.3558	11.79094	-6.86428	1.21E-05	7.07E-05	2.903616	
CPT_phageK_gp014	-1.01488	10.14895	-5.69826	7.63E-05	3.29E-04	1.228319	
CPT_phageK_gp012	-2.08629	8.006872	-8.85329	7.88E-07	8.74E-06	6.217093	
CPT_phageK_gp011	-1.67412	13.08195	-17.4649	2.46E-10	2.87E-08	14.09658	
CPT_phageK_gp010	-2.04912	11.65409	-15.4775	1.09E-09	4.14E-08	12.66602	
CPT_phageK_gp009	-1.00321	8.411111	-2.74667	0.016785	0.034007	-3.90416	
CPT_phageK_gp006	1.187206	7.188452	3.495859	0.004006	0.011384	-2.12474	
CPT_phageK_gp005	1.111627	8.778916	4.338624	8.24E-04	0.002866	-0.80067	
CPT_phageK_gp004	0.541674	9.697903	3.104663	0.008468	0.01952	-3.40563	
CPT_phageK_gp001	0.524543	10.34106	3.181086	0.007315	0.017392	-3.41038	

Table S2B: These Log2FC values were calculated comparing the counts from 4 replicates of RNA samples collected from phage K infections of NRS384WT at 2 and 5 min. Only genes showing a statistically significant change in expression (adj.p.value <=0.05) are shown.

**geneID**	**log2FC**	**AveExpr**	**t**	**P.Value**	**adj.P.Val**	**B**	
CPT_phageK_gt004	-1.18785	6.195944	-3.88847	0.002942	0.009979	-2.03715	
CPT_phageK_gt003	-1.21331	6.354928	-4.60171	9.44E-04	0.004603	-0.88108	
CPT_phageK_gt002	-1.45704	5.890821	-4.57348	9.86E-04	0.00471	-0.87923	
CPT_phageK_gp233	0.740849	10.84511	3.604053	0.004714	0.014141	-2.88821	

CPT_phageK_gp230	0.61437	13.17282	3.027711	0.012544	0.032615	-4.10534	
CPT_phageK_gp228	-1.09104	10.21671	-5.7568	1.74E-04	0.001236	0.611064	
CPT_phageK_gp211	0.546253	7.699281	2.947306	0.014405	0.037042	-3.75194	
CPT_phageK_gp210	0.643566	12.37682	4.636683	8.95E-04	0.004454	-1.32491	
CPT_phageK_gp208	0.529756	12.4062	4.053284	0.002249	0.008354	-2.29845	
CPT_phageK_gp204	0.414201	12.69575	3.461756	0.005987	0.016677	-3.33738	
CPT_phageK_gp197	0.321754	13.43438	2.903024	0.015547	0.039543	-4.33704	
CPT_phageK_gp193	-0.80432	10.0017	-3.52756	0.005359	0.015481	-2.94233	
CPT_phageK_gp192	-0.76625	7.150403	-3.97755	0.002544	0.0093	-1.97939	
CPT_phageK_gp190	-1.21117	4.178853	-4.15992	0.001894	0.007641	-1.37374	
CPT_phageK_gp189	-1.07594	7.079408	-5.5817	2.22E-04	0.001531	0.557131	
CPT_phageK_gp188	-1.33627	6.735612	-5.24251	3.61E-04	0.002285	0.079471	
CPT_phageK_gp187	-1.34848	6.235748	-6.38113	7.56E-05	6.10E-04	1.755504	
CPT_phageK_gp186	-1.06677	8.313	-5.15372	4.11E-04	0.002467	-0.15384	
CPT_phageK_gp185	-0.70955	7.902678	-4.27201	0.001583	0.006736	-1.53629	
CPT_phageK_gp184	-0.8065	8.829615	-3.48828	0.005725	0.016277	-2.91164	
CPT_phageK_gp183	-0.68602	12.12518	-4.12149	0.002015	0.007728	-2.20115	
CPT_phageK_gp181	-1.27651	2.631115	-3.43956	0.006215	0.016911	-2.44048	
CPT_phageK_gp176	-0.42526	11.29705	-3.22505	0.008946	0.024062	-3.63281	
CPT_phageK_gp175	-1.11329	11.67977	-7.86158	1.27E-05	1.94E-04	3.209522	
CPT_phageK_gp174	-0.91402	12.39124	-7.24651	2.58E-05	3.02E-04	2.392513	
CPT_phageK_gp173	-0.89554	14.77095	-3.6044	0.004711	0.014141	-3.12251	
CPT_phageK_gp172	-1.24157	7.219682	-6.85758	4.13E-05	4.05E-04	2.306374	
CPT_phageK_gp171	-0.77579	10.89764	-4.18746	0.001812	0.007439	-1.94036	
CPT_phageK_gp170	-0.87018	10.46726	-3.17691	0.009713	0.025538	-3.61303	
CPT_phageK_gp169	-0.75269	9.601292	-3.66197	0.004279	0.013351	-2.66823	
CPT_phageK_gp168	-1.02419	11.2573	-7.8197	1.33E-05	1.94E-04	3.207129	
CPT_phageK_gp165	-0.76991	11.00683	-3.78034	0.003516	0.011427	-2.64457	
CPT_phageK_gp161	-0.99723	11.1795	-4.45555	0.001185	0.005463	-1.54224	
CPT_phageK_gp160	-0.83305	9.089383	-3.93212	0.002739	0.009568	-2.1681	
CPT_phageK_gp155	0.690042	13.67731	4.441858	0.001211	0.005463	-1.69616	
CPT_phageK_gp154	-0.4343	11.41968	-3.44234	0.006186	0.016911	-3.27267	
CPT_phageK_gp150	0.55893	14.56321	3.965922	0.002592	0.009333	-2.4986	
CPT_phageK_gp149	0.367212	12.6695	2.837082	0.017419	0.043363	-4.42391	
CPT_phageK_gp146	1.021516	13.91972	6.418721	7.20E-05	6.02E-04	1.301182	
CPT_phageK_gp145	1.636818	9.482057	4.667461	8.53E-04	0.00434	-1.03145	
CPT_phageK_gp144	0.97502	13.65097	5.813481	1.61E-04	0.00119	0.444741	
CPT_phageK_gp143	0.63202	13.87366	4.758017	7.43E-04	0.003925	-1.1863	
CPT_phageK_gp141	0.447569	14.11828	4.391691	0.00131	0.005784	-1.78979	
CPT_phageK_gp132	0.593082	11.11012	4.290598	0.001537	0.006661	-1.75516	
CPT_phageK_gp131	1.0351	14.62458	6.763635	4.65E-05	4.18E-04	1.767326	
CPT_phageK_gp130	1.3311	13.49177	6.300119	8.40E-05	6.55E-04	1.152946	
CPT_phageK_gp129	1.274382	9.669203	6.698312	5.04E-05	4.37E-04	1.939967	
CPT_phageK_gp128	0.8099	10.67864	3.879469	0.002986	0.009983	-2.40132	
CPT_phageK_gp125	0.362909	15.90072	2.791787	0.018835	0.046394	-4.50005	
CPT_phageK_gp122	0.901387	10.80264	3.212173	0.009145	0.024318	-3.55948	
CPT_phageK_gp120	0.754766	15.84853	3.954613	0.002641	0.009362	-2.49312	

CPT_phageK_gp119	1.033559	14.02066	6.994941	3.49E-05	3.89E-04	2.072537	
CPT_phageK_gp118	0.871211	8.661856	5.411881	2.83E-04	0.001839	0.212697	
CPT_phageK_gp117	0.798567	11.41478	4.44017	0.001214	0.005463	-1.53462	
CPT_phageK_gp112	0.580094	13.03215	3.696067	0.004043	0.012785	-2.94168	
CPT_phageK_gp111	0.754428	14.4225	6.928169	3.79E-05	4.03E-04	1.977411	
CPT_phageK_gp109	0.646817	10.5139	3.483264	0.005773	0.016277	-3.07088	
CPT_phageK_gp101	0.328276	13.71522	2.886148	0.016006	0.040273	-4.3709	
CPT_phageK_gp099	-0.58394	9.884867	-3.60882	0.004676	0.014141	-2.79132	
CPT_phageK_gp097	-0.71056	7.943631	-4.25525	0.001626	0.006795	-1.56644	
CPT_phageK_gp082	0.920308	13.44265	5.209942	3.79E-04	0.002332	-0.4591	
CPT_phageK_gp079	-1.06169	9.972432	-5.50531	2.48E-04	0.001657	0.270689	
CPT_phageK_gp077	-1.1721	7.988582	-4.81071	6.86E-04	0.003732	-0.67742	
CPT_phageK_gp076	-0.5217	10.91972	-3.91566	0.002814	0.009684	-2.3955	
CPT_phageK_gp071	1.046951	12.39393	3.721401	0.003877	0.012426	-2.83565	
CPT_phageK_gp064	0.758997	15.23311	5.116324	4.34E-04	0.002542	-0.60747	
CPT_phageK_gp060	-1.05437	7.911406	-4.74749	7.55E-04	0.003925	-0.77222	
CPT_phageK_gp058	-0.60606	9.12317	-3.81422	0.003325	0.010957	-2.3755	
CPT_phageK_gp046	1.061823	10.97141	4.820736	6.75E-04	0.003732	-0.86748	
CPT_phageK_gp045	1.124836	8.621822	4.134788	0.001972	0.007691	-1.81045	
CPT_phageK_gp044	-1.84022	9.852495	-11.1534	5.15E-07	1.51E-05	6.775995	
CPT_phageK_gp043	-2.2761	11.26579	-13.0237	1.18E-07	6.88E-06	8.196475	
CPT_phageK_gp042	-1.56695	6.317673	-6.82098	4.33E-05	4.05E-04	2.327765	
CPT_phageK_gp040	-2.27097	8.67405	-12.1575	2.27E-07	8.87E-06	7.650609	
CPT_phageK_gp039	-2.50656	10.03804	-9.67427	1.94E-06	3.79E-05	5.377587	
CPT_phageK_gp038	-1.69024	12.216	-10.6754	7.78E-07	1.82E-05	6.135699	
CPT_phageK_gp037	-2.28351	10.53521	-15.9716	1.63E-08	1.90E-06	10.27783	
CPT_phageK_gp036	-1.44398	10.41043	-3.57235	0.004971	0.014539	-2.92154	
CPT_phageK_gp035	-1.93134	7.438949	-7.62235	1.66E-05	2.16E-04	3.240605	
CPT_phageK_gp034	-1.70607	10.30559	-9.34909	2.66E-06	4.45E-05	5.021356	
CPT_phageK_gp026	1.079893	10.55886	7.410503	2.12E-05	2.62E-04	2.817733	
CPT_phageK_gp024	1.695673	8.724877	4.062592	0.002216	0.008354	-1.9415	
CPT_phageK_gp022	1.053505	7.800977	5.806941	1.63E-04	0.00119	0.879496	
CPT_phageK_gp020	-1.70607	10.30559	-9.34909	2.66E-06	4.45E-05	5.021356	
CPT_phageK_gp019	-1.93134	7.438949	-7.62235	1.66E-05	2.16E-04	3.240605	
CPT_phageK_gp018	-1.44398	10.41043	-3.57235	0.004971	0.014539	-2.92154	
CPT_phageK_gp017	-2.28351	10.53521	-15.9716	1.63E-08	1.90E-06	10.27783	
CPT_phageK_gp016	-1.69024	12.216	-10.6754	7.78E-07	1.82E-05	6.135699	
CPT_phageK_gp015	-2.50656	10.03804	-9.67427	1.94E-06	3.79E-05	5.377587	
CPT_phageK_gp014	-2.27097	8.67405	-12.1575	2.27E-07	8.87E-06	7.650609	
CPT_phageK_gp012	-1.56695	6.317673	-6.82098	4.33E-05	4.05E-04	2.327765	
CPT_phageK_gp011	-2.2761	11.26579	-13.0237	1.18E-07	6.88E-06	8.196475	
CPT_phageK_gp010	-1.84022	9.852495	-11.1534	5.15E-07	1.51E-05	6.775995	
CPT_phageK_gp009	1.124836	8.621822	4.134788	0.001972	0.007691	-1.81045	
CPT_phageK_gp008	1.061823	10.97141	4.820736	6.75E-04	0.003732	-0.86748	

Table S2C: These Log2FC values were calculated comparing the counts from 4 replicates of RNA samples collected from phage K infections of NRS384WT at 5 and 10 min. Only genes showing a statistically significant change in expression (adj.p.value <=0.05) are shown.

geneID	log2FC	AveExpr	t	P.Value	adj.P.Val	B	
CPT_phageK_gt004	2.121576	6.302565	9.247233	4.07E-06	4.76E-05	4.655758	
CPT_phageK_gt003	2.22266	6.471626	12.17697	3.44E-07	8.95E-06	7.229065	
CPT_phageK_gt002	1.447277	5.48842	5.503642	2.92E-04	0.001062	0.203035	
CPT_phageK_gp230	-0.9976	12.68313	-4.7917	8.01E-04	0.002373	-1.40854	
CPT_phageK_gp229	-1.02825	7.801377	-4.3242	0.001618	0.004116	-1.84405	
CPT_phageK_gp228	-0.72231	9.027128	-4.24619	0.001825	0.004449	-2.00791	
CPT_phageK_gp227	0.990608	5.648696	3.067604	0.012322	0.021358	-3.72359	
CPT_phageK_gp220	-0.36874	13.06005	-2.68308	0.023577	0.037531	-4.93365	
CPT_phageK_gp218	0.984287	9.436893	3.554732	0.005493	0.010535	-3.19359	
CPT_phageK_gp217	0.840077	11.142	4.396753	0.001448	0.00388	-1.91784	
CPT_phageK_gp215	0.601666	8.767514	3.042135	0.01286	0.021966	-4.02277	
CPT_phageK_gp213	-0.47713	13.24452	-5.51653	2.87E-04	0.001062	-0.33187	
CPT_phageK_gp212	0.732087	8.767228	4.460507	0.001314	0.003618	-1.65508	
CPT_phageK_gp211	0.559765	7.997984	4.443034	0.001349	0.003672	-1.65775	
CPT_phageK_gp210	0.428422	12.61003	3.715327	0.00423	0.008608	-3.15261	
CPT_phageK_gp209	0.319867	11.71056	2.519066	0.031123	0.048598	-5.13104	
CPT_phageK_gp206	0.431167	13.57899	2.894007	0.016504	0.027007	-4.57647	
CPT_phageK_gp199	1.212193	12.04607	7.476618	2.53E-05	1.92E-04	2.343625	
CPT_phageK_gp197	0.306375	13.4508	3.095588	0.011757	0.020531	-4.23168	
CPT_phageK_gp195	0.529495	13.58649	3.866395	0.003318	0.007058	-2.92163	
CPT_phageK_gp193	1.449682	10.03063	8.190106	1.17E-05	1.09E-04	3.305306	
CPT_phageK_gp192	2.782312	7.894709	19.90933	3.50E-09	8.18E-07	11.8451	
CPT_phageK_gp190	0.728695	3.674887	3.011723	0.013535	0.022785	-3.57706	
CPT_phageK_gp189	0.459193	6.500058	2.850141	0.017773	0.028486	-4.20381	
CPT_phageK_gp188	0.691218	6.143984	3.259523	0.00894	0.016183	-3.46399	
CPT_phageK_gp187	2.406598	6.506176	13.93622	9.93E-08	5.56E-06	8.505811	
CPT_phageK_gp186	2.388715	8.688752	15.78462	3.11E-08	2.43E-06	9.632051	
CPT_phageK_gp185	1.900761	8.241583	12.52806	2.65E-07	8.74E-06	7.421165	
CPT_phageK_gp184	1.853099	9.066965	8.928998	5.53E-06	6.17E-05	4.150002	
CPT_phageK_gp183	2.184909	12.57138	13.14071	1.71E-07	6.67E-06	7.705961	
CPT_phageK_gp182	1.711562	10.16309	5.977929	1.55E-04	6.54E-04	0.528115	
CPT_phageK_gp181	2.534681	3.018955	8.370246	9.69E-06	9.86E-05	3.951049	
CPT_phageK_gp180	2.438094	11.86266	12.36596	2.99E-07	8.74E-06	7.146316	
CPT_phageK_gp179	2.162256	13.91968	16.10307	2.58E-08	2.43E-06	9.686181	
CPT_phageK_gp178	0.988279	6.282078	4.085708	0.002344	0.005224	-2.08984	
CPT_phageK_gp177	1.554094	8.264851	6.809568	5.47E-05	3.56E-04	1.752138	
CPT_phageK_gp176	1.800306	11.68541	13.67015	1.19E-07	5.56E-06	8.125975	
CPT_phageK_gp175	1.723819	11.68527	11.47911	5.89E-07	1.25E-05	6.416221	
CPT_phageK_gp174	1.730027	12.4996	11.98071	3.99E-07	9.34E-06	6.793024	
CPT_phageK_gp173	1.354202	14.70182	6.050371	1.41E-04	6.34E-04	0.441307	
CPT_phageK_gp172	0.739102	6.711439	4.707663	9.07E-04	0.002588	-1.14077	
CPT_phageK_gp171	1.00301	10.71209	5.957189	1.59E-04	6.54E-04	0.471446	

CPT_phageK_gp170	1.121727	10.29951	4.83388	7.53E-04	0.002288	-1.15409	
CPT_phageK_gp169	0.95546	9.411248	5.371457	3.50E-04	0.001222	-0.29384	
CPT_phageK_gp168	0.503999	10.69808	4.142635	0.002144	0.005017	-2.29268	
CPT_phageK_gp167	0.617757	7.966548	3.301509	0.008338	0.015242	-3.55134	
CPT_phageK_gp166	0.53062	13.94787	2.966202	0.014613	0.024251	-4.45389	
CPT_phageK_gp165	0.57151	10.61347	3.377771	0.007348	0.013646	-3.56646	
CPT_phageK_gp163	-1.49641	9.485682	-5.49526	2.95E-04	0.001062	-0.09865	
CPT_phageK_gp162	-1.10076	7.969682	-6.0021	1.50E-04	6.54E-04	0.671589	
CPT_phageK_gp160	1.175051	8.966472	6.179424	1.19E-04	6.07E-04	0.879916	
CPT_phageK_gp159	0.955185	9.899355	4.041598	0.002513	0.005495	-2.40189	
CPT_phageK_gp158	0.687595	11.91525	4.882246	7.02E-04	0.00216	-1.21482	
CPT_phageK_gp157	-0.42991	12.54379	-3.69914	0.004342	0.00876	-3.18192	
CPT_phageK_gp154	-0.4494	10.67956	-4.36026	0.001531	0.003937	-1.94383	
CPT_phageK_gp152	-0.59963	11.25178	-5.56308	2.69E-04	0.001032	-0.15045	
CPT_phageK_gp148	0.628918	16.6008	2.882559	0.016826	0.027343	-4.5547	
CPT_phageK_gp147	0.567897	6.591286	3.898191	0.003154	0.006771	-2.43818	
CPT_phageK_gp146	0.684505	14.47096	4.770995	8.26E-04	0.002416	-1.45231	
CPT_phageK_gp144	0.765564	14.2186	5.054854	5.47E-04	0.001753	-1.01622	
CPT_phageK_gp142	0.379377	13.23326	3.68286	0.004458	0.008917	-3.22702	
CPT_phageK_gp140	-0.5906	12.43942	-6.33846	9.76E-05	5.20E-04	0.851547	
CPT_phageK_gp139	-0.85058	10.71096	-5.90229	1.71E-04	6.89E-04	0.382936	
CPT_phageK_gp135	0.344969	11.57375	2.518505	0.031153	0.048598	-5.12118	
CPT_phageK_gp133	0.710617	12.12547	3.654582	0.004668	0.009102	-3.22281	
CPT_phageK_gp132	0.532045	11.38475	4.935365	6.49E-04	0.002026	-1.0952	
CPT_phageK_gp131	0.718214	15.20074	5.154447	4.74E-04	0.001563	-0.84915	
CPT_phageK_gp130	0.830205	14.26791	4.268407	0.001764	0.004344	-2.25665	
CPT_phageK_gp129	0.831333	10.45483	5.955479	1.59E-04	6.54E-04	0.488665	
CPT_phageK_gp128	1.063644	11.35114	6.413271	8.88E-05	5.20E-04	1.040682	
CPT_phageK_gp127	0.481752	10.93301	3.952813	0.002891	0.006264	-2.62911	
CPT_phageK_gp120	0.520407	16.19008	3.146251	0.0108	0.019002	-4.10857	
CPT_phageK_gp119	0.75076	14.61165	6.636554	6.75E-05	4.27E-04	1.228889	
CPT_phageK_gp118	0.942523	9.290032	8.010435	1.41E-05	1.18E-04	3.145674	
CPT_phageK_gp117	1.074457	12.05233	8.122758	1.25E-05	1.13E-04	3.096511	
CPT_phageK_gp116	0.736756	10.45251	6.318441	1.00E-04	5.20E-04	0.986063	
CPT_phageK_gp115	0.95275	11.29503	4.983199	6.06E-04	0.001917	-1.00769	
CPT_phageK_gp114	0.831183	6.124889	5.055617	5.46E-04	0.001753	-0.54422	
CPT_phageK_gp111	0.302873	14.65159	2.9174	0.015865	0.026144	-4.52944	
CPT_phageK_gp110	0.769405	11.08938	4.106177	0.00227	0.005108	-2.38714	
CPT_phageK_gp109	0.502496	10.82971	2.99781	0.013855	0.023158	-4.23711	
CPT_phageK_gp104	0.333023	10.939	2.643581	0.025208	0.039856	-4.85268	
CPT_phageK_gp103	0.794053	10.49458	3.170095	0.010378	0.018398	-3.90942	
CPT_phageK_gp098	-0.34465	11.88032	-3.53117	0.005709	0.01086	-3.42566	
CPT_phageK_gp093	-0.68777	8.800314	-3.79119	0.003743	0.007821	-2.75799	
CPT_phageK_gp092	-1.05174	6.724827	-4.75789	8.42E-04	0.002432	-1.07145	
CPT_phageK_gp091	-0.87386	9.507088	-4.55341	0.001142	0.003181	-1.52836	
CPT_phageK_gp090	-0.86439	10.68131	-5.17404	4.61E-04	0.001542	-0.674	
CPT_phageK_gp089	-0.79173	9.880768	-5.95518	1.59E-04	6.54E-04	0.537143	

CPT_phageK_gp088	-0.71918	8.378534	-3.6306	0.004853	0.009386	-3.01609	
CPT_phageK_gp087	-0.94495	8.881503	-3.84134	0.003454	0.007281	-2.6739	
CPT_phageK_gp086	-0.77785	12.48566	-8.01304	1.41E-05	1.18E-04	2.932919	
CPT_phageK_gp085	-1.00727	9.430646	-3.04277	0.012847	0.021966	-4.04017	
CPT_phageK_gp084	-0.93413	11.57316	-6.12167	1.28E-04	6.14E-04	0.613342	
CPT_phageK_gp082	-0.43538	13.42328	-2.50413	0.03192	0.049465	-5.24319	
CPT_phageK_gp079	-0.98094	8.692154	-6.32923	9.87E-05	5.20E-04	1.090315	
CPT_phageK_gp078	-1.22815	8.758292	-4.073	0.002391	0.005279	-2.28781	
CPT_phageK_gp077	-0.73074	6.776661	-3.36635	0.007488	0.013797	-3.35722	
CPT_phageK_gp076	-1.06565	9.825491	-8.46414	8.80E-06	9.36E-05	3.640463	
CPT_phageK_gp075	-1.35806	9.048344	-4.39173	0.001459	0.00388	-1.77345	
CPT_phageK_gp067	-0.65272	11.4762	-4.20462	0.001947	0.004696	-2.26983	
CPT_phageK_gp066	-0.87848	8.638557	-5.54736	2.75E-04	0.001037	-6.84E-04	
CPT_phageK_gp060	-1.12801	6.561265	-5.44709	3.15E-04	0.001117	-0.01557	
CPT_phageK_gp059	-1.76952	8.305596	-4.13171	0.002181	0.005053	-2.17507	
CPT_phageK_gp058	-1.22561	7.942021	-8.23688	1.11E-05	1.08E-04	3.450453	
CPT_phageK_gp057	-1.35038	6.809431	-3.25617	0.00899	0.016183	-3.54674	
CPT_phageK_gp056	-0.92978	5.489967	-3.75919	0.003941	0.00809	-2.55583	
CPT_phageK_gp055	-0.53434	10.45499	-3.01455	0.013471	0.022785	-4.17756	
CPT_phageK_gp054	-0.8257	10.74636	-3.76946	0.003876	0.008027	-2.92465	
CPT_phageK_gp053	-0.6868	9.994424	-6.13373	1.27E-04	6.14E-04	0.774862	
CPT_phageK_gp052	-0.64392	7.954636	-5.56748	2.67E-04	0.001032	0.055385	
CPT_phageK_gp051	-1.31283	8.917814	-6.05462	1.40E-04	6.34E-04	0.715874	
CPT_phageK_gp050	-0.87377	9.42027	-6.4125	8.89E-05	5.20E-04	1.190771	
CPT_phageK_gp049	-0.87431	8.50403	-7.4697	2.55E-05	1.92E-04	2.541934	
CPT_phageK_gp048	-0.80871	7.147005	-3.49561	0.006051	0.011328	-3.17562	
CPT_phageK_gp047	-0.87568	9.140843	-4.15742	0.002095	0.004952	-2.15361	
CPT_phageK_gp046	-0.61222	10.92409	-3.66461	0.004592	0.009031	-3.11845	
CPT_phageK_gp044	-1.42025	7.9624	-10.9152	9.30E-07	1.67E-05	6.090616	
CPT_phageK_gp043	-1.22907	9.25426	-6.94098	4.68E-05	3.13E-04	1.879484	
CPT_phageK_gp042	-1.48593	4.531141	-6.36621	9.42E-05	5.20E-04	1.478849	
CPT_phageK_gp040	-1.19651	6.691108	-7.07017	4.02E-05	2.85E-04	2.16068	
CPT_phageK_gp039	-0.95358	8.055296	-4.37207	0.001504	0.00391	-1.77581	
CPT_phageK_gp038	-1.50496	10.35474	-10.4583	1.37E-06	2.13E-05	5.592958	
CPT_phageK_gp037	-1.19848	8.542418	-9.81841	2.40E-06	3.10E-05	5.066246	
CPT_phageK_gp036	-1.47381	8.643337	-4.11838	0.002227	0.00506	-2.20769	
CPT_phageK_gp035	-1.0635	5.686608	-5.19216	4.50E-04	0.001525	-0.30439	
CPT_phageK_gp034	-1.29248	8.546849	-9.76488	2.52E-06	3.10E-05	5.015346	
CPT_phageK_gp033	-1.53843	7.039465	-4.29397	0.001695	0.00422	-1.83598	
CPT_phageK_gp032	0.617786	10.06495	3.225739	0.009458	0.016894	-3.77992	
CPT_phageK_gp031	0.931589	9.106521	4.819076	7.70E-04	0.002309	-1.10845	
CPT_phageK_gp030	-0.48027	11.52083	-4.67571	9.51E-04	0.002681	-1.51707	
CPT_phageK_gp025	0.581887	10.24104	2.87587	0.017017	0.027462	-4.39037	
CPT_phageK_gp021	-1.53843	7.039465	-4.29397	0.001695	0.00422	-1.83598	
CPT_phageK_gp020	-1.29248	8.546849	-9.76488	2.52E-06	3.10E-05	5.015346	
CPT_phageK_gp019	-1.0635	5.686608	-5.19216	4.50E-04	0.001525	-0.30439	
CPT_phageK_gp018	-1.47381	8.643337	-4.11838	0.002227	0.00506	-2.20769	

CPT_phageK_gp017	-1.19848	8.542418	-9.81841	2.40E-06	3.10E-05	5.066246	
CPT_phageK_gp016	-1.50496	10.35474	-10.4583	1.37E-06	2.13E-05	5.592958	
CPT_phageK_gp015	-0.95358	8.055296	-4.37207	0.001504	0.00391	-1.77581	
CPT_phageK_gp014	-1.19651	6.691108	-7.07017	4.02E-05	2.85E-04	2.16068	
CPT_phageK_gp012	-1.48593	4.531141	-6.36621	9.42E-05	5.20E-04	1.478849	
CPT_phageK_gp011	-1.22907	9.25426	-6.94098	4.68E-05	3.13E-04	1.879484	
CPT_phageK_gp010	-1.42025	7.9624	-10.9152	9.30E-07	1.67E-05	6.090616	
CPT_phageK_gp008	-0.61222	10.92409	-3.66461	0.004592	0.009031	-3.11845	
CPT_phageK_gp007	-0.87568	9.140843	-4.15742	0.002095	0.004952	-2.15361	
CPT_phageK_gp006	-0.80871	7.147005	-3.49561	0.006051	0.011328	-3.17562	
CPT_phageK_gp005	-0.87431	8.50403	-7.4697	2.55E-05	1.92E-04	2.541934	
CPT_phageK_gp004	-0.87377	9.42027	-6.4125	8.89E-05	5.20E-04	1.190771	
CPT_phageK_gp003	-1.31283	8.917814	-6.05462	1.40E-04	6.34E-04	0.715874	
CPT_phageK_gp002	-0.64392	7.954636	-5.56748	2.67E-04	0.001032	0.055385	
CPT_phageK_gp001	-0.6868	9.994424	-6.13373	1.27E-04	6.14E-04	0.774862	

Table S2D: These Log2FC values were calculated comparing the counts from 4 replicates of RNA samples collected from phage K infections of NRS384WT at 10 and 20 min. Only genes showing a statistically significant change in expression (adj.p.value <=0.05) are shown.

**geneID**	**log2FC**	**AveExpr**	**t**	**P.Value**	**adj.P.Val**	**B**	
CPT_phageK_gt002	-1.6026	4.762141	-6.58448	6.01E-05	1.72E-04	1.646342	
CPT_phageK_gt001	-0.32786	9.232318	-2.64255	0.024503	0.036754	-5.1917	
CPT_phageK_gp233	-0.94917	9.620391	-5.1695	4.11E-04	9.43E-04	-0.97728	
CPT_phageK_gp231	-0.98643	5.701337	-6.23227	9.46E-05	2.60E-04	1.060322	
CPT_phageK_gp230	-1.58013	10.84176	-7.87794	1.30E-05	4.21E-05	2.647594	
CPT_phageK_gp228	-0.75116	7.755829	-7.75718	1.48E-05	4.76E-05	2.825519	
CPT_phageK_gp227	2.032777	6.47676	15.20284	2.85E-08	2.38E-07	9.609777	
CPT_phageK_gp226	1.397138	8.149918	9.62717	2.14E-06	8.50E-06	4.859293	
CPT_phageK_gp224	-0.22458	11.07677	-3.02331	0.012734	0.02041	-4.67432	
CPT_phageK_gp223	-0.64096	6.817467	-5.83475	1.61E-04	4.02E-04	0.371432	
CPT_phageK_gp222	-0.83366	5.615227	-2.9823	0.013662	0.0216	-4.13684	
CPT_phageK_gp221	-1.37441	11.44526	-11.1145	5.66E-07	2.94E-06	6.024633	
CPT_phageK_gp220	-1.22018	11.72103	-16.8088	1.08E-08	1.20E-07	10.30178	
CPT_phageK_gp218	0.848739	9.724428	8.322286	7.98E-06	2.75E-05	3.220501	
CPT_phageK_gp217	0.280367	11.18161	3.142148	0.010393	0.017709	-4.47291	
CPT_phageK_gp216	0.608499	11.65934	3.284079	0.008163	0.014694	-4.23663	
CPT_phageK_gp215	0.709435	8.738177	5.808131	1.67E-04	4.11E-04	0.090099	
CPT_phageK_gp214	-0.41234	6.888653	-3.4413	0.006259	0.011533	-3.48713	
CPT_phageK_gp213	-0.98229	11.92626	-10.3581	1.09E-06	4.95E-06	5.30945	
CPT_phageK_gp212	0.713031	8.791915	5.151637	4.22E-04	9.58E-04	-0.90835	
CPT_phageK_gp211	0.53117	7.861259	3.204549	0.009344	0.016312	-4.00426	
CPT_phageK_gp209	-0.31888	11.15945	-3.17018	0.009908	0.017047	-4.42019	
CPT_phageK_gp208	-0.6914	11.38647	-10.5467	9.23E-07	4.41E-06	5.482295	
CPT_phageK_gp207	-1.11118	5.681548	-5.35228	3.16E-04	7.46E-04	-0.21639	
CPT_phageK_gp205	-0.32026	12.57733	-3.04905	0.012185	0.019862	-4.608	

CPT_phageK_gp204	-0.40975	11.84638	-4.19533	0.001817	0.003633	-2.66463	
CPT_phageK_gp203	-0.38858	6.585901	-2.78627	0.019133	0.028885	-4.59242	
CPT_phageK_gp199	1.631006	12.90703	16.21738	1.52E-08	1.55E-07	9.957335	
CPT_phageK_gp197	-0.55418	12.75941	-7.06306	3.33E-05	1.01E-04	1.642726	
CPT_phageK_gp195	1.853984	14.18093	12.65643	1.66E-07	1.05E-06	7.478544	
CPT_phageK_gp193	2.910213	11.68033	19.355	2.70E-09	3.96E-08	11.79751	
CPT_phageK_gp192	0.369775	8.789403	2.99129	0.013453	0.021414	-4.52427	
CPT_phageK_gp191	1.845543	3.490725	6.308984	8.56E-05	2.38E-04	1.39548	
CPT_phageK_gp190	4.003843	5.36556	22.48734	6.17E-10	1.31E-08	13.52263	
CPT_phageK_gp189	4.742764	8.484534	36.45204	5.05E-12	3.94E-10	18.25842	
CPT_phageK_gp188	4.79233	8.255859	37.74948	3.56E-12	3.94E-10	18.56636	
CPT_phageK_gp187	1.560786	7.796266	8.844593	4.63E-06	1.69E-05	4.072317	
CPT_phageK_gp186	1.253625	9.973017	11.31764	4.77E-07	2.54E-06	6.251245	
CPT_phageK_gp185	1.780477	9.388476	10.52888	9.38E-07	4.41E-06	5.59529	
CPT_phageK_gp184	1.619716	10.28168	14.86732	3.54E-08	2.86E-07	9.046009	
CPT_phageK_gp183	1.466705	13.80388	10.23395	1.22E-06	5.19E-06	5.293275	
CPT_phageK_gp182	1.854655	11.37914	10.34964	1.10E-06	4.95E-06	5.299134	
CPT_phageK_gp181	1.084736	4.160568	5.202117	3.92E-04	9.08E-04	-0.28197	
CPT_phageK_gp180	0.575873	12.84561	4.901855	6.10E-04	0.001346	-1.468	
CPT_phageK_gp179	0.593985	14.7165	4.456534	0.001204	0.002537	-2.0244	
CPT_phageK_gp178	2.24112	7.310769	14.58758	4.25E-08	3.21E-07	9.13265	
CPT_phageK_gp177	1.607518	9.284671	14.11619	5.84E-08	4.27E-07	8.594669	
CPT_phageK_gp176	1.802735	12.90098	16.55342	1.25E-08	1.33E-07	10.17305	
CPT_phageK_gp175	2.785729	13.37426	19.87455	2.08E-09	3.48E-08	12.12142	
CPT_phageK_gp174	3.143689	14.36699	21.36593	1.02E-09	1.99E-08	12.92603	
CPT_phageK_gp173	3.404201	16.50027	19.01054	3.23E-09	4.44E-08	11.78172	
CPT_phageK_gp172	4.320575	8.573138	15.45101	2.44E-08	2.13E-07	9.647842	
CPT_phageK_gp171	2.737563	12.00369	17.2327	8.44E-09	1.03E-07	10.5826	
CPT_phageK_gp170	2.698094	11.63102	17.17691	8.71E-09	1.03E-07	10.54106	
CPT_phageK_gp169	2.800246	10.73563	23.89924	3.38E-10	7.90E-09	14.00966	
CPT_phageK_gp168	3.997628	12.37391	34.75398	8.13E-12	4.76E-10	17.86389	
CPT_phageK_gp167	4.402497	9.791153	31.01146	2.54E-11	1.19E-09	16.72899	
CPT_phageK_gp166	3.46949	15.35997	21.11915	1.15E-09	2.06E-08	12.84174	
CPT_phageK_gp165	3.994222	12.37796	45.16067	5.92E-13	1.38E-10	20.41616	
CPT_phageK_gp163	-0.72271	7.868234	-7.07233	3.29E-05	1.01E-04	1.95582	
CPT_phageK_gp160	2.832066	10.45042	23.91176	3.36E-10	7.90E-09	14.02732	
CPT_phageK_gp159	3.14754	11.41799	28.31011	6.28E-11	2.45E-09	15.76661	
CPT_phageK_gp158	2.627408	12.98206	24.13821	3.06E-10	7.90E-09	14.12929	
CPT_phageK_gp157	0.851296	12.16134	10.19828	1.26E-06	5.27E-06	5.143679	
CPT_phageK_gp156	0.84355	10.70599	10.52415	9.42E-07	4.41E-06	5.475631	
CPT_phageK_gp155	0.2618	13.35933	2.603677	0.026198	0.038799	-5.32098	
CPT_phageK_gp154	1.184701	10.47865	15.88689	1.86E-08	1.74E-07	9.723385	
CPT_phageK_gp153	1.021813	9.448248	7.075381	3.28E-05	1.01E-04	1.730153	
CPT_phageK_gp152	0.814217	10.80113	6.149432	1.05E-04	0.000284	0.379079	
CPT_phageK_gp151	0.817544	11.80515	8.116577	9.97E-06	3.38E-05	2.897278	
CPT_phageK_gp149	0.425166	12.44795	4.467985	0.001182	0.002515	-2.19551	
CPT_phageK_gp147	0.634919	6.528213	5.412	2.90E-04	6.92E-04	-0.22923	

CPT_phageK_gp145	0.38968	9.976147	3.585227	0.004918	0.009207	-3.64887	
CPT_phageK_gp140	-1.13492	10.99983	-14.7388	3.85E-08	3.00E-07	8.926815	
CPT_phageK_gp139	-1.14646	9.167074	-10.8872	6.87E-07	3.49E-06	5.966378	
CPT_phageK_gp138	-0.98844	12.86469	-13.5579	8.60E-08	5.75E-07	8.093718	
CPT_phageK_gp137	-1.0278	10.99164	-12.5885	1.75E-07	1.08E-06	7.293179	
CPT_phageK_gp136	-0.97456	12.34288	-10.3239	1.13E-06	4.97E-06	5.286253	
CPT_phageK_gp133	-0.3979	11.73256	-4.09883	0.00212	0.004204	-2.82865	
CPT_phageK_gp131	-0.36043	14.78592	-3.02743	0.012645	0.020406	-4.45738	
CPT_phageK_gp130	-0.34663	13.92663	-2.46792	0.033082	0.048383	-5.49924	
CPT_phageK_gp129	0.456133	10.47967	4.024981	0.002388	0.004696	-2.92287	
CPT_phageK_gp128	0.715211	11.60565	5.831839	1.62E-04	4.02E-04	-0.10328	
CPT_phageK_gp127	0.602292	10.93071	4.320756	0.001489	0.003057	-2.44312	
CPT_phageK_gp126	1.935951	7.605747	9.394531	2.68E-06	1.01E-05	4.679981	
CPT_phageK_gp125	-1.13843	14.43869	-10.2868	1.16E-06	5.04E-06	5.399443	
CPT_phageK_gp122	-0.41442	9.801141	-2.79436	0.018868	0.02867	-4.99636	
CPT_phageK_gp121	-1.04348	11.67185	-11.9672	2.82E-07	1.65E-06	6.772465	
CPT_phageK_gp120	-0.75035	15.48931	-5.33448	3.24E-04	7.58E-04	-0.55847	
CPT_phageK_gp119	-0.44256	14.18057	-4.72666	7.94E-04	0.001737	-1.63283	
CPT_phageK_gp118	0.409598	9.378373	3.260722	0.008493	0.015172	-4.1485	
CPT_phageK_gp117	0.265675	12.16619	2.615674	0.025663	0.038249	-5.38295	
CPT_phageK_gp116	2.233182	11.38291	25.40812	1.84E-10	6.15E-09	14.63222	
CPT_phageK_gp115	1.886896	12.17787	12.2976	2.18E-07	1.31E-06	7.059458	
CPT_phageK_gp114	2.942967	7.311753	17.15251	8.83E-09	1.03E-07	10.78801	
CPT_phageK_gp112	-0.8346	12.30938	-6.05093	1.20E-04	3.06E-04	0.242065	
CPT_phageK_gp111	-0.41085	14.01065	-3.13929	0.010444	0.017709	-4.33434	
CPT_phageK_gp109	0.334728	10.60179	3.44437	0.006227	0.011533	-3.92359	
CPT_phageK_gp108	-0.43831	9.93298	-5.71347	1.90E-04	4.63E-04	-0.20043	
CPT_phageK_gp107	-0.60795	11.04024	-6.05196	1.20E-04	3.06E-04	0.233027	
CPT_phageK_gp106	-0.87621	10.88773	-6.91359	3.99E-05	1.20E-04	1.42211	
CPT_phageK_gp105	-0.54445	11.8632	-4.35638	0.001408	0.002915	-2.39397	
CPT_phageK_gp101	-0.69725	12.88814	-8.08932	1.03E-05	3.43E-05	2.922279	
CPT_phageK_gp100	1.620344	7.962381	6.675454	5.36E-05	1.55E-04	1.420392	
CPT_phageK_gp098	-0.62474	10.8582	-7.48008	2.04E-05	6.44E-05	2.146964	
CPT_phageK_gp092	-1.19838	4.922598	-6.90253	4.05E-05	1.20E-04	2.047215	
CPT_phageK_gp091	-0.55693	8.232121	-3.25423	0.008588	0.015223	-3.97254	
CPT_phageK_gp090	-0.78934	9.256596	-6.31381	8.51E-05	2.38E-04	0.748624	
CPT_phageK_gp089	-0.91564	8.340465	-9.14438	3.43E-06	1.27E-05	4.328088	
CPT_phageK_gp087	-0.67404	7.482698	-3.76327	0.003661	0.006908	-2.9953	
CPT_phageK_gp086	-1.494	10.80186	-19.4398	2.59E-09	3.96E-08	11.83494	
CPT_phageK_gp084	-1.17926	9.985867	-9.40831	2.64E-06	1.01E-05	4.420364	
CPT_phageK_gp082	-0.6459	12.20877	-3.80628	0.003411	0.006489	-3.31095	
CPT_phageK_gp078	-0.56779	7.298691	-2.87371	0.016462	0.025342	-4.51476	
CPT_phageK_gp077	-0.52255	5.454301	-3.85454	0.003151	0.006044	-2.61624	
CPT_phageK_gp076	-1.20801	8.160105	-12.6959	1.61E-07	1.05E-06	7.633597	
CPT_phageK_gp075	-0.84874	7.310338	-6.21381	9.69E-05	2.64E-04	0.862965	
CPT_phageK_gp072	-0.33272	9.628101	-3.23473	0.008877	0.015618	-4.21583	
CPT_phageK_gp071	-0.86209	11.08451	-6.05329	1.20E-04	3.06E-04	0.235373	

CPT_phageK_gp070	-0.31964	11.43935	-3.04725	0.012223	0.019862	-4.64209	
CPT_phageK_gp069	-0.41347	5.774375	-2.84616	0.01726	0.026398	-4.39793	
CPT_phageK_gp068	-0.89186	7.569117	-8.35235	7.73E-06	2.70E-05	3.546591	
CPT_phageK_gp067	-0.50469	10.2286	-5.62333	2.15E-04	5.19E-04	-0.35445	
CPT_phageK_gp066	-0.64802	7.215841	-3.2004	0.009411	0.016312	-3.93814	
CPT_phageK_gp065	-0.92092	10.73691	-10.6585	8.37E-07	4.17E-06	5.604823	
CPT_phageK_gp064	-1.06612	14.24704	-6.78251	4.69E-05	1.37E-04	1.397426	
CPT_phageK_gp059	-0.5697	6.479537	-2.58487	0.027059	0.039823	-4.92458	
CPT_phageK_gp055	-0.73959	9.230702	-7.99499	1.14E-05	3.75E-05	2.913698	
CPT_phageK_gp054	-0.95625	9.321937	-9.57069	2.26E-06	8.82E-06	4.653381	
CPT_phageK_gp053	-0.78579	8.593631	-6.12333	1.09E-04	2.87E-04	0.574028	
CPT_phageK_gp052	-0.43842	6.732838	-3.30609	0.007865	0.014266	-3.70603	
CPT_phageK_gp051	-0.784	7.188702	-5.01292	5.17E-04	0.001152	-0.90619	
CPT_phageK_gp050	-1.02433	7.846817	-9.79269	1.83E-06	7.39E-06	5.06052	
CPT_phageK_gp049	-0.53919	7.122354	-3.12456	0.01071	0.0179	-4.05963	
CPT_phageK_gp047	-0.60467	7.699983	-2.93977	0.014696	0.022925	-4.4462	
CPT_phageK_gp046	-1.15886	9.45055	-13.9075	6.73E-08	4.63E-07	8.435167	
CPT_phageK_gp045	-2.02608	7.151048	-11.4668	4.22E-07	2.35E-06	6.707922	
CPT_phageK_gp044	-0.556	6.318642	-3.98161	0.002562	0.004954	-2.50058	
CPT_phageK_gp042	-1.12769	2.556862	-4.20447	0.00179	0.003612	-1.70759	
CPT_phageK_gp040	-0.59106	5.139895	-3.11079	0.010964	0.018068	-3.86052	
CPT_phageK_gp038	-0.45123	8.744959	-4.47038	0.001178	0.002515	-1.98031	
CPT_phageK_gp032	1.361414	10.4159	16.014	1.72E-08	1.68E-07	9.810304	
CPT_phageK_gp031	1.998768	10.03225	15.44177	2.45E-08	2.13E-07	9.467571	
CPT_phageK_gp030	-0.93633	10.25086	-11.3863	4.51E-07	2.45E-06	6.302873	
CPT_phageK_gp028	-0.67791	8.676356	-2.8969	0.015819	0.024514	-4.65711	
CPT_phageK_gp027	-0.6869	9.431484	-8.65225	5.64E-06	2.03E-05	3.642196	
CPT_phageK_gp026	-0.92068	9.805215	-8.40124	7.34E-06	2.60E-05	3.325055	
CPT_phageK_gp025	-0.5689	9.732901	-4.35749	0.001405	0.002915	-2.3036	
CPT_phageK_gp016	-0.45123	8.744959	-4.47038	0.001178	0.002515	-1.98031	
CPT_phageK_gp014	-0.59106	5.139895	-3.11079	0.010964	0.018068	-3.86052	
CPT_phageK_gp012	-1.12769	2.556862	-4.20447	0.00179	0.003612	-1.70759	
CPT_phageK_gp010	-0.556	6.318642	-3.98161	0.002562	0.004954	-2.50058	
CPT_phageK_gp009	-2.02608	7.151048	-11.4668	4.22E-07	2.35E-06	6.707922	
CPT_phageK_gp008	-1.15886	9.45055	-13.9075	6.73E-08	4.63E-07	8.435167	
CPT_phageK_gp007	-0.60467	7.699983	-2.93977	0.014696	0.022925	-4.4462	
CPT_phageK_gp005	-0.53919	7.122354	-3.12456	0.01071	0.0179	-4.05963	
CPT_phageK_gp004	-1.02433	7.846817	-9.79269	1.83E-06	7.39E-06	5.06052	
CPT_phageK_gp003	-0.784	7.188702	-5.01292	5.17E-04	0.001152	-0.90619	
CPT_phageK_gp002	-0.43842	6.732838	-3.30609	0.007865	0.014266	-3.70603	
CPT_phageK_gp001	-0.78579	8.593631	-6.12333	1.09E-04	2.87E-04	0.574028	

Table S2E: These Log2FC values were calculated comparing the counts from 4 replicates of RNA samples collected from phage K infections of NRS384WT at 20 and 30 min. Only genes showing a statistically significant change in expression (adj.p.value <=0.05) are shown.

geneID	log2FC	AveExpr	t	P.Value	adj.P.Val	B	
CPT_phageK_gt004	-0.88356	5.506401	-3.97605	0.002097	0.007291	-2.04579	
CPT_phageK_gt003	-0.87513	5.395205	-3.09616	0.009968	0.024194	-3.62196	
CPT_phageK_gp232	-0.69526	5.516449	-3.5308	0.00458	0.013014	-2.84815	
CPT_phageK_gp231	-0.97593	3.625618	-4.26711	0.001273	0.004785	-1.34686	
CPT_phageK_gp228	-0.625	6.098707	-2.81417	0.016572	0.037489	-4.18369	
CPT_phageK_gp227	2.010188	7.421611	10.22797	5.05E-07	1.07E-05	6.559962	
CPT_phageK_gp226	1.723732	8.786695	10.22279	5.07E-07	1.07E-05	6.478723	
CPT_phageK_gp225	-0.70914	10.41661	-4.52929	8.20E-04	0.003411	-1.41463	
CPT_phageK_gp224	-0.61663	9.729144	-4.94052	4.19E-04	0.002268	-0.67836	
CPT_phageK_gp223	-0.65607	5.103485	-3.20783	0.008155	0.020442	-3.39809	
CPT_phageK_gp221	-0.72412	9.444798	-4.65498	6.66E-04	0.003103	-1.14675	
CPT_phageK_gp220	-0.73686	9.809116	-4.97498	3.96E-04	0.002251	-0.62446	
CPT_phageK_gp218	-0.9711	8.561008	-4.00631	0.00199	0.007134	-2.21264	
CPT_phageK_gp217	-0.90105	9.927158	-3.94231	0.002223	0.007297	-2.42511	
CPT_phageK_gp215	-1.22642	7.381751	-5.42473	1.96E-04	0.00147	0.284223	
CPT_phageK_gp214	-0.90279	5.160911	-4.95279	4.10E-04	0.002268	-0.32848	
CPT_phageK_gp213	-0.72968	10.10911	-5.33839	2.23E-04	0.001627	-0.03788	
CPT_phageK_gp212	0.748088	8.344524	4.169046	0.001505	0.005478	-1.92252	
CPT_phageK_gp211	0.824466	7.462913	4.829431	5.01E-04	0.002537	-0.70754	
CPT_phageK_gp210	1.972447	12.39438	10.33358	4.54E-07	1.07E-05	6.454763	
CPT_phageK_gp209	-0.69302	9.717806	-5.26574	2.50E-04	0.001713	-0.13733	
CPT_phageK_gp208	-0.44814	9.868088	-3.20753	0.008159	0.020442	-3.75332	
CPT_phageK_gp206	-0.36081	11.94789	-2.66671	0.021615	0.047512	-4.82157	
CPT_phageK_gp205	-0.45475	11.2541	-3.67263	0.003564	0.010784	-2.98507	
CPT_phageK_gp203	-0.767	4.921516	-3.31986	0.006671	0.017663	-3.17558	
CPT_phageK_gp202	-1.0304	9.540861	-4.37161	0.001067	0.004076	-1.64353	
CPT_phageK_gp198	-0.65098	7.638882	-4.56883	7.68E-04	0.003351	-1.16351	
CPT_phageK_gp197	-0.64794	11.21159	-5.74339	1.21E-04	0.001004	0.553551	
CPT_phageK_gp196	-0.7445	9.108697	-4.38744	0.001039	0.004035	-1.5858	
CPT_phageK_gp195	2.049092	15.16767	10.54324	3.70E-07	1.07E-05	6.719131	
CPT_phageK_gp193	1.928577	13.17103	10.99389	2.41E-07	8.01E-06	7.129912	
CPT_phageK_gp192	-1.43617	7.163609	-5.44184	1.91E-04	0.00147	0.328212	
CPT_phageK_gp191	1.537988	4.066715	6.406469	4.63E-05	4.31E-04	2.032526	
CPT_phageK_gp190	1.554985	7.035228	9.66685	8.95E-07	1.49E-05	5.980267	
CPT_phageK_gp189	1.74154	10.64995	11.65367	1.31E-07	6.12E-06	7.807672	
CPT_phageK_gp188	1.533287	10.32367	8.311905	4.01E-06	5.84E-05	4.194179	
CPT_phageK_gp181	-0.7445	3.249953	-3.43942	0.005388	0.014598	-2.78236	
CPT_phageK_gp180	-0.64694	11.88644	-3.74678	0.003128	0.009717	-2.86828	
CPT_phageK_gp178	1.892197	8.324083	6.00999	8.15E-05	7.03E-04	1.135676	
CPT_phageK_gp175	1.023903	14.33532	7.934351	6.30E-06	8.15E-05	3.682018	
CPT_phageK_gp174	0.923173	15.45426	8.339703	3.88E-06	5.84E-05	4.231089	
CPT_phageK_gp173	1.349917	17.92797	7.252287	1.48E-05	1.72E-04	2.887181	
CPT_phageK_gp172	1.479958	10.3282	7.061855	1.90E-05	1.99E-04	2.542651	
CPT_phageK_gp171	1.42986	13.12953	7.034256	1.97E-05	1.99E-04	2.440564	
CPT_phageK_gp170	1.103987	12.57474	6.893006	2.38E-05	2.31E-04	2.242767	
CPT_phageK_gp169	0.952796	11.67032	7.18049	1.63E-05	1.80E-04	2.660309	

CPT_phageK_gp168	1.385012	14.11507	11.34697	1.73E-07	6.73E-06	7.493254	
CPT_phageK_gp167	1.42377	11.57849	8.09497	5.19E-06	7.11E-05	3.876244	
CPT_phageK_gp166	2.033778	17.15566	9.95823	6.63E-07	1.28E-05	6.158646	
CPT_phageK_gp165	2.030117	14.45782	12.98056	4.24E-08	2.47E-06	8.99283	
CPT_phageK_gp161	-0.84927	7.860912	-2.95539	0.012846	0.030543	-4.06063	
CPT_phageK_gp159	0.446414	12.2867	3.971544	0.002114	0.007291	-2.47157	
CPT_phageK_gp158	0.527263	13.59551	3.802044	0.002839	0.009062	-2.77502	
CPT_phageK_gp154	0.83748	10.54348	7.375703	1.26E-05	1.55E-04	2.959753	
CPT_phageK_gp153	0.66912	9.369199	3.875428	0.002498	0.008083	-2.50747	
CPT_phageK_gp152	0.794529	10.66317	4.873969	4.66E-04	0.002447	-0.85965	
CPT_phageK_gp151	0.653356	11.58564	5.229681	2.65E-04	0.001713	-0.29142	
CPT_phageK_gp150	0.506424	13.41431	3.454064	0.005249	0.01439	-3.40895	
CPT_phageK_gp149	0.803036	12.137	6.116845	6.98E-05	6.26E-04	1.106854	
CPT_phageK_gp148	0.716688	16.05667	4.230401	0.001355	0.005012	-1.9268	
CPT_phageK_gp147	0.590092	6.074496	3.706446	0.003358	0.010294	-2.5792	
CPT_phageK_gp146	0.666599	13.69193	4.540447	8.05E-04	0.003409	-1.46497	
CPT_phageK_gp145	1.003603	9.689628	3.59221	0.004108	0.011963	-3.04045	
CPT_phageK_gp144	1.001542	13.58344	5.193526	2.80E-04	0.001764	-0.3621	
CPT_phageK_gp140	-0.46076	9.249383	-3.56436	0.004315	0.012413	-3.0656	
CPT_phageK_gp138	-0.55011	11.13608	-5.07939	3.35E-04	0.002004	-0.52106	
CPT_phageK_gp137	-0.82902	9.126841	-5.24252	2.59E-04	0.001713	-0.13616	
CPT_phageK_gp136	-0.73351	10.53666	-5.6816	1.32E-04	0.001064	0.493652	
CPT_phageK_gp135	-0.59143	10.09978	-2.92244	0.013633	0.032085	-4.28079	
CPT_phageK_gp133	-0.51762	10.34084	-3.95089	0.002191	0.007291	-2.4339	
CPT_phageK_gp132	-0.38341	9.805252	-3.14407	0.009145	0.022668	-3.86481	
CPT_phageK_gp125	-0.44173	12.68196	-3.51499	0.00471	0.013212	-3.29463	
CPT_phageK_gp124	0.501612	7.228121	2.765816	0.018082	0.04051	-4.35705	
CPT_phageK_gp121	-0.72411	9.864882	-4.69067	6.28E-04	0.002986	-1.10974	
CPT_phageK_gp119	-0.41944	12.79224	-3.12495	0.009465	0.023214	-4.00309	
CPT_phageK_gp118	-0.50802	8.293241	-3.3028	0.006878	0.018006	-3.46658	
CPT_phageK_gp117	-0.80009	10.95539	-4.70027	6.18E-04	0.002986	-1.14717	
CPT_phageK_gp116	3.016486	13.0697	28.30094	8.87E-12	2.07E-09	17.66385	
CPT_phageK_gp115	3.197638	13.79013	17.87758	1.37E-09	1.60E-07	12.57925	
CPT_phageK_gp114	2.86163	9.066627	13.59016	2.61E-08	2.03E-06	9.585994	
CPT_phageK_gp112	-0.43919	10.73043	-2.70384	0.020217	0.044863	-4.70907	
CPT_phageK_gp104	0.411033	9.830068	3.358606	0.006224	0.016669	-3.47677	
CPT_phageK_gp100	2.329012	8.974657	9.886383	7.13E-07	1.28E-05	6.110997	
CPT_phageK_gp095	-0.37641	8.489799	-2.64331	0.022544	0.048742	-4.66862	
CPT_phageK_gp087	-0.55494	5.834501	-2.88401	0.014611	0.033376	-4.04079	
CPT_phageK_gp077	-1.04627	3.509641	-4.62685	6.97E-04	0.003187	-0.71376	
CPT_phageK_gp076	-0.80383	6.231617	-5.05501	3.49E-04	0.002031	-0.23771	
CPT_phageK_gp074	-0.83348	5.824433	-3.95979	0.002157	0.007291	-2.09933	
CPT_phageK_gp073	-0.74027	5.001427	-4.50269	8.57E-04	0.003503	-1.08101	
CPT_phageK_gp070	-0.85512	9.908449	-3.95713	0.002167	0.007291	-2.39808	
CPT_phageK_gp069	-0.6468	4.178624	-2.95953	0.012751	0.030543	-3.74699	
CPT_phageK_gp065	-0.80623	8.921168	-5.24304	2.59E-04	0.001713	-0.11856	
CPT_phageK_gp055	-0.66932	7.491402	-3.50872	0.004763	0.013212	-3.0364	

CPT_phageK_gp053	-0.67062	6.722864	-3.63468	0.00381	0.011238	-2.75153	
CPT_phageK_gp052	-0.82786	5.016234	-4.56186	7.77E-04	0.003351	-0.97909	
CPT_phageK_gp049	-0.72721	5.336625	-3.27723	0.0072	0.018436	-3.2919	
CPT_phageK_gp048	-0.85752	3.987963	-2.90829	0.013985	0.032263	-3.81488	
CPT_phageK_gp044	0.799318	5.312882	4.404996	0.001009	0.003985	-1.28379	
CPT_phageK_gp032	0.804968	10.38229	5.175378	2.88E-04	0.001767	-0.33756	
CPT_phageK_gp030	-0.57937	8.55138	-4.70883	6.10E-04	0.002986	-0.98681	
CPT_phageK_gp027	-0.65984	7.832256	-4.8652	4.73E-04	0.002447	-0.66958	
CPT_phageK_gp026	-0.44284	8.19755	-2.6421	0.022593	0.048742	-4.64838	
CPT_phageK_gp025	-0.91403	8.070832	-3.75583	0.003079	0.009693	-2.62561	
CPT_phageK_gp010	0.799318	5.312882	4.404996	0.001009	0.003985	-1.28379	
CPT_phageK_gp006	-0.85752	3.987963	-2.90829	0.013985	0.032263	-3.81488	
CPT_phageK_gp005	-0.72721	5.336625	-3.27723	0.0072	0.018436	-3.2919	
CPT_phageK_gp002	-0.82786	5.016234	-4.56186	7.77E-04	0.003351	-0.97909	
CPT_phageK_gp001	-0.67062	6.722864	-3.63468	0.00381	0.011238	-2.75153	

Table S2F: These Log2FC values were calculated comparing the counts from 4 replicates of RNA samples collected from phage K infections of NRS384WT at 30 and 40 min. Only genes showing a statistically significant change in expression (adj.p.value <=0.05) are shown.

**geneID**	**log2FC**	**AveExpr**	**t**	**P.Value**	**adj.P.Val**	**B**	
CPT_phageK_gp232	-0.73158	4.161287	-3.32926	0.007804	0.049147	-2.53858	
CPT_phageK_gp227	1.329811	8.474041	6.247416	1.03E-04	0.003055	1.592438	
CPT_phageK_gp226	1.08168	9.55149	6.452833	7.91E-05	0.003055	1.775327	
CPT_phageK_gp216	-0.80096	9.489366	-6.04695	1.33E-04	0.003102	1.251778	
CPT_phageK_gp214	-0.87065	3.631531	-3.35888	0.007428	0.048074	-2.44264	
CPT_phageK_gp211	0.866315	7.682988	6.225389	1.06E-04	0.003055	1.60756	
CPT_phageK_gp210	0.771427	13.17197	3.592536	0.005043	0.038991	-2.80951	
CPT_phageK_gp205	-0.57009	10.13503	-5.18297	4.34E-04	0.005984	-0.04791	
CPT_phageK_gp204	-0.86471	9.373147	-6.13949	1.18E-04	0.003055	1.387903	
CPT_phageK_gp201	-0.86964	5.899626	-3.39988	0.006937	0.046182	-2.53814	
CPT_phageK_gp193	0.624516	13.82248	4.253592	0.001745	0.018672	-1.73653	
CPT_phageK_gp179	-0.58212	13.10717	-3.95285	0.002809	0.025173	-2.19751	
CPT_phageK_gp155	0.318331	12.33408	3.410622	0.006814	0.046182	-3.07876	
CPT_phageK_gp151	0.386732	11.50178	3.574199	0.005198	0.038991	-2.74572	
CPT_phageK_gp143	-0.52335	11.96055	-3.70325	0.004206	0.035002	-2.55525	
CPT_phageK_gp142	-0.42275	11.13158	-5.30817	3.64E-04	0.005649	0.052438	
CPT_phageK_gp141	-0.46026	11.18426	-4.58392	0.00105	0.01223	-1.06025	
CPT_phageK_gp139	-0.64343	6.337216	-3.54626	0.005443	0.038991	-2.31949	
CPT_phageK_gp138	-0.51955	10.01079	-5.8679	1.69E-04	0.003275	0.950565	
CPT_phageK_gp136	-0.59318	9.280683	-5.51495	2.72E-04	0.004533	0.527023	
CPT_phageK_gp127	-0.56329	9.100637	-4.21873	0.001843	0.018672	-1.42949	
CPT_phageK_gp125	-0.46418	11.64607	-6.26069	1.01E-04	0.003055	1.365409	
CPT_phageK_gp124	-0.84941	6.367818	-4.22988	0.001811	0.018672	-1.21341	
CPT_phageK_gp120	-0.73468	12.8531	-6.34742	9.04E-05	0.003055	1.42385	
CPT_phageK_gp119	-0.66058	11.66592	-6.88005	4.68E-05	0.003055	2.174306	

CPT_phageK_gp118	-0.52167	7.115311	-3.53746	0.005522	0.038991	-2.37907	
CPT_phageK_gp116	0.802885	14.36471	10.93494	8.06E-07	1.16E-04	6.334082	
CPT_phageK_gp115	1.077114	15.30598	10.68826	9.93E-07	1.16E-04	6.089288	
CPT_phageK_gp114	1.20144	10.45033	5.874088	1.67E-04	0.003275	0.915396	
CPT_phageK_gp112	-0.4846	9.650171	-4.01409	0.002547	0.023739	-1.82859	
CPT_phageK_gp111	-0.5154	11.7846	-4.82949	7.27E-04	0.009406	-0.71703	
CPT_phageK_gp108	0.524881	8.583621	4.178325	0.001964	0.019066	-1.44087	
CPT_phageK_gp100	1.26862	10.11383	5.1795	4.37E-04	0.005984	-0.05652	
CPT_phageK_gp098	0.444311	9.495218	3.852448	0.003301	0.028489	-2.08759	
CPT_phageK_gp067	-0.68663	7.725762	-3.65855	0.004525	0.036358	-2.21928	
CPT_phageK_gp064	-0.76536	11.53491	-5.61981	2.36E-04	0.004227	0.481588	
CPT_phageK_gp026	-0.72758	6.956651	-4.75942	8.06E-04	0.009888	-0.41921	
