# Supplementary material for: The Transcriptional Program of Staphylococcus aureus Phage K Is Affected by a Host rpoC Mutation That Confers Phage K Resistance
Source: Viruses. 2024 Nov 13;16(11):1773. doi: 10.3390/v16111773 (PMC11598898; doi:10.3390/v16111773)
Supplement: Supplementary file 1 [file viruses-16-01773-s001.zip › Table S3.pdf]

Table S3A: These Log2FC values were calculated comparing the counts from 4 replicates of RNA samples collected from phage K infections of NRS384WT and *rpoC* G17D at 2 min. Only genes showing a Log2FC <-1 or >1 are being shown here.

geneID	log2FC	AveExpr	t	P.Value	adj.P.Val	B	TU#	Classification
CPT_phageK_gp189	-5.80851	4.894316	-16.1376	5.30E-10	1.24E-08	12.77228	24	Late
CPT_phageK_gp188	-5.48365	4.836431	-9.53849	3.01E-07	2.14E-06	7.17678	24	Late
CPT_phageK_gp173	-4.5493	13.18212	-22.7531	6.93E-12	8.61E-10	17.7618	27	Late
CPT_phageK_gp190	-3.85594	2.996723	-7.9939	2.20E-06	1.06E-05	5.254748	24	Late
CPT_phageK_gp115	-3.63569	9.66051	-17.766	1.59E-10	4.67E-09	14.67051	41	Late
CPT_phageK_gp166	-3.59382	12.89461	-19.9697	3.64E-11	2.45E-09	16.05856	28	Late
CPT_phageK_gp193	-3.44808	8.958622	-16.8134	3.17E-10	8.29E-09	13.98541	21	Late
CPT_phageK_gp175	-3.2979	10.85191	-22.6532	7.33E-12	8.61E-10	17.73437	27	Late
CPT_phageK_gp116	-3.2706	9.265276	-18.1985	1.18E-10	3.94E-09	14.97221	41	Late
CPT_phageK_gp165	-3.18289	10.09301	-19.2409	5.82E-11	2.74E-09	15.66954	28	Late
CPT_phageK_gp174	-2.93705	11.62882	-19.754	4.17E-11	2.45E-09	15.9688	27	Late
CPT_phageK_gp168	-2.58687	10.73185	-18.2119	1.16E-10	3.94E-09	14.95349	28	Late
CPT_phageK_gp114	-2.23501	5.445855	-6.5315	1.87E-05	5.79E-05	3.126237	41	Late
CPT_phageK_gp178	-1.99599	5.525951	-5.00705	2.37E-04	5.07E-04	0.556326	26	Late
CPT_phageK_gp167	-1.95319	7.695283	-8.17367	1.72E-06	8.80E-06	5.302428	28	Late
CPT_phageK_gp227	-1.7251	5.300683	-4.57265	5.18E-04	9.80E-04	-0.20417	11	Late
CPT_phageK_gp171	-1.52508	10.77713	-10.9163	6.22E-08	6.35E-07	8.433959	28	Late
CPT_phageK_gp207	-1.46947	6.185545	-5.44815	1.10E-04	2.54E-04	1.225791	16	Middle
CPT_phageK_gp176	-1.45964	11.02554	-10.9986	5.69E-08	6.22E-07	8.499748	26	Late
CPT_phageK_gp031	-1.41928	9.031267	-6.62859	1.61E-05	5.05E-05	2.795711	7	Late
CPT_phageK_gp230	-1.33722	12.47149	-10.1921	1.39E-07	1.17E-06	7.392107	10	Middle
CPT_phageK_gp160	-1.28836	9.161246	-7.6378	3.62E-06	1.57E-05	4.338468	31	Late
CPT_phageK_gp170	-1.22603	10.53638	-8.44359	1.20E-06	7.42E-06	5.351378	28	Late
CPT_phageK_gp143	-1.22202	13.19101	-11.5862	3.06E-08	3.93E-07	8.933208	36	Middle
CPT_phageK_gp032	-1.16269	9.157012	-6.66294	1.53E-05	4.85E-05	2.835417	7	Late
CPT_phageK_gp183	-1.15839	12.12366	-10.2524	1.30E-07	1.13E-06	7.50268	25	Late
CPT_phageK_gp152	-1.14721	11.63042	-10.7772	7.24E-08	7.09E-07	8.172809	34	Middle
CPT_phageK_gp158	-1.06505	11.92793	-8.45175	1.19E-06	7.42E-06	5.192101	32	Late
CPT_phageK_gp044	1.045999	11.45165	6.44156	2.16E-05	6.14E-05	2.190904	51	Early
CPT_phageK_gp010	1.045999	11.45165	6.44156	2.16E-05	6.14E-05	2.190904	3	Early
CPT_phageK_gp058	1.092911	10.11899	7.439626	4.80E-06	1.91E-05	3.944913	48	Middle
CPT_phageK_gp200	1.099693	9.712135	7.331243	5.61E-06	2.11E-05	3.828099	17	Middle
CPT_phageK_gp059	1.115147	10.26331	5.858881	5.52E-05	1.43E-04	1.370445	48	Middle
CPT_phageK_gp034	1.167019	11.8573	7.609193	3.77E-06	1.58E-05	3.975261	53	Early
CPT_phageK_gp020	1.167019	11.8573	7.609193	3.77E-06	1.58E-05	3.975261	5	Early
CPT_phageK_gp102	1.175983	12.00318	10.43601	1.06E-07	9.55E-07	7.725675	43	Middle
CPT_phageK_gp096	1.17665	12.46121	11.54846	3.18E-08	3.93E-07	8.948344	45	Middle
CPT_phageK_gp056	1.236041	7.02446	5.769063	6.41E-05	1.62E-04	1.626161	48	Middle
CPT_phageK_gp100	1.257199	8.779511	6.937367	1.01E-05	3.58E-05	3.3004	44	Late
CPT_phageK_gp060	1.269419	9.20755	8.320591	1.41E-06	7.55E-06	5.310026	48	Middle
CPT_phageK_gp085	1.29193	11.12584	8.36725	1.33E-06	7.55E-06	5.160801	45	Middle
CPT_phageK_gp062	1.317338	10.33116	7.339312	5.55E-06	2.11E-05	3.766502	48	Middle
CPT_phageK_gp048	1.343276	8.718074	8.303869	1.45E-06	7.55E-06	5.330132	49	Middle

CPT_phageK_gp006	1.343276	8.718074	8.303869	1.45E-06	7.55E-06	5.330132	1	Middle
CPT_phageK_gp091	1.348767	11.4902	10.97659	5.82E-08	6.22E-07	8.412092	45	Middle
CPT_phageK_gp088	1.351397	10.13982	7.462635	4.64E-06	1.88E-05	3.978279	45	Middle
CPT_phageK_gp063	1.365528	7.831481	6.467812	2.07E-05	6.14E-05	2.669262	48	Middle
CPT_phageK_gp182	1.369832	11.04105	9.478272	3.24E-07	2.24E-06	6.656744	25	Late
CPT_phageK_gp219	1.376219	11.4201	5.323704	1.36E-04	3.08E-04	0.270386	12	Middle
CPT_phageK_gp222	1.40699	7.677831	6.453454	2.12E-05	6.14E-05	2.669163	12	Middle
CPT_phageK_gp087	1.432623	10.98991	11.40746	3.68E-08	4.33E-07	8.946609	45	Middle
CPT_phageK_gp134	1.455825	12.60588	7.973143	2.26E-06	1.06E-05	4.432094	37	Middle
CPT_phageK_gp024	1.466963	8.733131	7.824377	2.78E-06	1.26E-05	4.648052	6	Middle
CPT_phageK_gp113	1.509196	8.685453	7.328087	5.64E-06	2.11E-05	3.916674	42	Middle
CPT_phageK_gp045	1.562444	9.056079	5.738144	6.74E-05	1.67E-04	1.294897	50	Middle
CPT_phageK_gp009	1.562444	9.056079	5.738144	6.74E-05	1.67E-04	1.294897	2	Middle
CPT_phageK_gp037	1.570016	12.58149	12.4684	1.26E-08	1.75E-07	9.912634	53	Early
CPT_phageK_gp017	1.570016	12.58149	12.4684	1.26E-08	1.75E-07	9.912634	5	Early
CPT_phageK_gp194	1.613181	10.00471	3.781562	0.002272	0.003813	-2.42842	21	Late
CPT_phageK_gp164	1.631087	5.396182	3.348501	0.005211	0.008164	-2.5291	30	Middle
CPT_phageK_gp081	1.733978	6.239631	6.438504	2.17E-05	6.14E-05	2.870007	45	Middle
CPT_phageK_gp035	1.746676	9.444094	8.371301	1.32E-06	7.55E-06	5.35692	53	Early
CPT_phageK_gp019	1.746676	9.444094	8.371301	1.32E-06	7.55E-06	5.35692	5	Early
CPT_phageK_gp029	1.992782	9.443505	9.71973	2.42E-07	1.84E-06	7.128508	6	Middle
CPT_phageK_gp041	2.026258	8.816836	8.080538	1.95E-06	9.57E-06	5.018409	52	Early
CPT_phageK_gp013	2.026258	8.816836	8.080538	1.95E-06	9.57E-06	5.018409	4	Early
CPT_phageK_gp057	2.078886	9.452305	10.50407	9.79E-08	9.21E-07	8.071556	48	Middle
CPT_phageK_gp033	2.333984	10.33434	14.58938	1.85E-09	3.63E-08	12.13427	53	Early
CPT_phageK_gp021	2.333984	10.33434	14.58938	1.85E-09	3.63E-08	12.13427	5	Early
CPT_phageK_gp036	2.530307	12.71972	13.80452	3.66E-09	5.73E-08	11.22232	53	Early
CPT_phageK_gp018	2.530307	12.71972	13.80452	3.66E-09	5.73E-08	11.22232	5	Early
CPT_phageK_gp094	2.921107	3.811637	6.474917	2.05E-05	6.14E-05	3.11426	45	Middle
CPT_phageK_gp061	2.944219	6.306846	9.783355	2.25E-07	1.76E-06	7.495859	48	Middle
CPT_phageK_gp191	3.113527	7.113708	7.890324	2.54E-06	1.17E-05	4.997843	22	Late
CPT_phageK_gp083	3.804422	9.014878	14.18736	2.61E-09	4.72E-08	11.86767	45	Middle

Table S3B: These Log2FC values were calculated comparing the counts from 4 replicates of RNA samples collected from phage K infections of NRS384WT and *rpoC* G17D at 5 min. Only genes showing a Log2FC <-1 or >1 are being shown here.

geneID	log2FC	AveExpr	t	P.Value	adj.P.Val	B	TU#	Classification
CPT_phageK_gp189	-5.39637	3.905065	-19.912	2.91E-09	6.82E-07	11.69508	24	Late
CPT_phageK_gp188	-5.12859	3.553513	-13.2394	1.40E-07	4.38E-06	8.161739	24	Late
CPT_phageK_gp190	-4.20038	1.467859	-9.12224	4.19E-06	3.02E-05	4.8134	24	Late
CPT_phageK_gp173	-3.89399	12.50346	-15.3608	3.46E-08	1.36E-06	9.321465	27	Late
CPT_phageK_gp166	-3.37961	12.41737	-16.0573	2.27E-08	1.33E-06	9.762405	28	Late
CPT_phageK_gp193	-2.76271	8.400121	-12.7693	1.96E-07	5.09E-06	7.689472	21	Late
CPT_phageK_gp165	-2.50093	9.559298	-11.567	4.89E-07	8.80E-06	6.657699	29	Late
CPT_phageK_gp115	-2.49744	10.05338	-9.77017	2.27E-06	2.15E-05	5.001064	41	Late
CPT_phageK_gp175	-2.47616	10.0296	-18.0303	7.54E-09	8.82E-07	11.02907	27	Late

CPT_phageK_gp174	-2.28999	10.9266	-16.9238	1.38E-08	1.08E-06	10.37227	27	Late
CPT_phageK_gp230	-2.1957	12.53367	-10.7395	9.65E-07	1.25E-05	5.712406	10	Middle
CPT_phageK_gp168	-2.0706	9.852072	-15.3494	3.48E-08	1.36E-06	9.44558	28	Late
CPT_phageK_gp207	-1.9175	6.673547	-5.91066	1.60E-04	4.81E-04	0.667475	16	Middle
CPT_phageK_gp171	-1.83233	9.729163	-10.419	1.27E-06	1.56E-05	5.64358	28	Late
CPT_phageK_gp116	-1.70214	9.693773	-9.504	2.91E-06	2.43E-05	4.762096	41	Late
CPT_phageK_gp167	-1.5193	7.149115	-7.15712	3.40E-05	1.39E-04	2.268262	28	Late
CPT_phageK_gp143	-1.49763	13.55938	-13.1412	1.50E-07	4.38E-06	7.615806	36	Middle
CPT_phageK_gp178	-1.38486	5.443337	-4.42948	0.001335	0.002498	-1.42732	26	Late
CPT_phageK_gp042	-1.38388	4.869934	-4.30902	0.001607	0.002926	-1.55655	51	Early
CPT_phageK_gp012	-1.38388	4.869934	-4.30902	0.001607	0.002926	-1.55655	3	Early
CPT_phageK_gp071	-1.35525	12.27743	-5.10728	4.87E-04	0.001163	-0.96673	46	Middle
CPT_phageK_gp144	-1.29926	13.6088	-8.93389	5.03E-06	3.47E-05	3.806195	35	Middle
CPT_phageK_gp152	-1.28184	11.36261	-11.2679	6.22E-07	9.70E-06	6.293179	34	Middle
CPT_phageK_gp064	-1.26998	15.10592	-12.1444	3.12E-07	7.30E-06	6.754055	47	Middle
CPT_phageK_gp210	-1.2028	12.26144	-10.8738	8.61E-07	1.25E-05	5.850738	15	Late
CPT_phageK_gp176	-1.19958	10.60893	-9.40777	3.18E-06	2.57E-05	4.607624	26	Late
CPT_phageK_gp183	-1.18553	11.30819	-7.44358	2.44E-05	1.10E-04	2.353014	25	Late
CPT_phageK_gp106	-1.16508	11.52956	-4.25303	0.001754	0.003086	-2.23314	43	Middle
CPT_phageK_gp170	-1.1644	9.589716	-4.64178	9.66E-04	0.0019	-1.42487	28	Late
CPT_phageK_gp112	-1.13419	12.89709	-7.32278	2.80E-05	1.19E-04	2.012546	42	Middle
CPT_phageK_gp177	-1.12927	7.341536	-4.65083	9.53E-04	0.00189	-1.30518	26	Late
CPT_phageK_gp032	-1.04291	9.529734	-5.20688	4.22E-04	0.001029	-0.54128	7	Late
CPT_phageK_gp111	-1.03037	14.41299	-9.75767	2.30E-06	2.15E-05	4.61129	42	Middle
CPT_phageK_gp026	-1.0223	10.77728	-7.91614	1.44E-05	7.35E-05	2.967376	6	Middle
CPT_phageK_gp150	-1.00309	14.45776	-8.51798	7.65E-06	5.12E-05	3.30588	34	Middle
CPT_phageK_gp134	1.016193	12.93865	2.544629	0.029526	0.040882	-5.31161	37	Middle
CPT_phageK_gp196	1.040313	11.69877	7.162342	3.38E-05	1.39E-04	1.960812	20	Middle
CPT_phageK_gp229	1.064053	9.078809	4.440467	0.001312	0.002476	-1.72431	10	Middle
CPT_phageK_gp074	1.086568	9.259713	6.274881	9.99E-05	3.41E-04	1.000518	46	Middle
CPT_phageK_gp052	1.113172	9.117177	8.024086	1.29E-05	6.93E-05	3.200908	49	Middle
CPT_phageK_gp002	1.113172	9.117177	8.024086	1.29E-05	6.93E-05	3.200908	1	Middle
CPT_phageK_gp099	1.119199	10.12447	9.31824	3.47E-06	2.70E-05	4.560418	45	Middle
CPT_phageK_gp222	1.138366	8.408911	2.889392	0.016422	0.024391	-4.31735	12	Middle
CPT_phageK_gp181	1.147382	2.620127	3.472269	0.006167	0.009817	-2.69505	25	Late
CPT_phageK_gp069	1.195241	7.809869	6.26066	1.02E-04	3.41E-04	1.041768	47	Middle
CPT_phageK_gp126	1.229813	7.777387	6.071413	1.30E-04	4.05E-04	0.78532	38	Late
CPT_phageK_gp211	1.313128	8.670661	9.795559	2.22E-06	2.15E-05	5.095113	15	Late
CPT_phageK_gp077	1.31516	8.030017	5.837498	1.77E-04	5.17E-04	0.440814	46	Middle
CPT_phageK_gp093	1.336708	10.07247	7.647923	1.94E-05	9.46E-05	2.714458	45	Middle
CPT_phageK_gp102	1.40386	12.02091	10.3399	1.36E-06	1.59E-05	5.385477	43	Middle
CPT_phageK_gp219	1.479733	11.14964	5.444205	3.02E-04	7.86E-04	-0.32876	12	Middle
CPT_phageK_gp097	1.500057	8.374531	9.19823	3.89E-06	2.94E-05	4.505927	45	Middle
CPT_phageK_gp073	1.553982	8.631793	10.27657	1.44E-06	1.60E-05	5.556709	46	Middle
CPT_phageK_gp215	1.557103	9.522653	7.677303	1.88E-05	9.35E-05	2.767167	14	Middle
CPT_phageK_gp079	1.589591	10.25614	10.75627	9.51E-07	1.25E-05	5.932921	46	Middle
CPT_phageK_gp041	1.59948	8.813477	4.775505	7.91E-04	0.001624	-1.18574	52	Early

CPT_phageK_gp013	1.59948	8.813477	4.775505	7.91E-04	0.001624	-1.18574	4	Early
CPT_phageK_gp058	1.615777	9.631723	11.30175	6.05E-07	9.70E-06	6.436216	48	Middle
CPT_phageK_gp200	1.653675	9.605055	7.406658	2.55E-05	1.11E-04	2.435538	17	Middle
CPT_phageK_gp085	1.663408	11.05477	5.626116	2.35E-04	6.72E-04	-0.05053	45	Middle
CPT_phageK_gp091	1.677622	11.20035	9.517491	2.87E-06	2.43E-05	4.664654	45	Middle
CPT_phageK_gp182	1.69063	10.5986	5.544357	2.63E-04	7.15E-04	-0.1148	25	Late
CPT_phageK_gp062	1.734175	10.13635	5.537207	2.66E-04	7.15E-04	-0.09517	48	Middle
CPT_phageK_gp100	1.737537	9.03334	4.919736	6.39E-04	0.001412	-0.97697	44	Late
CPT_phageK_gp087	1.739747	10.60942	7.580771	2.09E-05	9.79E-05	2.595688	45	Middle
CPT_phageK_gp035	1.74767	7.393656	8.245585	1.02E-05	6.10E-05	3.553501	53	Early
CPT_phageK_gp019	1.74767	7.393656	8.245585	1.02E-05	6.10E-05	3.553501	5	Early
CPT_phageK_gp037	1.751314	10.25306	11.56795	4.89E-07	8.80E-06	6.641802	53	Early
CPT_phageK_gp017	1.751314	10.25306	11.56795	4.89E-07	8.80E-06	6.641802	5	Early
CPT_phageK_gp033	1.756759	8.934987	4.928328	6.31E-04	0.001407	-0.95824	53	Early
CPT_phageK_gp021	1.756759	8.934987	4.928328	6.31E-04	0.001407	-0.95824	5	Early
CPT_phageK_gp081	1.757164	6.695113	4.290535	0.001654	0.002978	-1.79474	45	Middle
CPT_phageK_gp023	1.759367	4.395459	5.978037	1.47E-04	4.52E-04	1.009563	6	Middle
CPT_phageK_gp048	1.770864	8.666539	7.990663	1.33E-05	6.93E-05	3.176775	49	Middle
CPT_phageK_gp006	1.770864	8.666539	7.990663	1.33E-05	6.93E-05	3.176775	1	Middle
CPT_phageK_gp056	1.792269	7.126827	7.024133	3.97E-05	1.52E-04	2.121043	48	Middle
CPT_phageK_gp187	1.829872	6.445761	8.34901	9.12E-06	5.77E-05	3.739913	24	Late
CPT_phageK_gp088	1.850565	9.940094	9.903816	2.01E-06	2.14E-05	5.141467	45	Middle
CPT_phageK_gp060	1.943397	8.360128	9.601668	2.65E-06	2.39E-05	4.922757	48	Middle
CPT_phageK_gp113	1.986226	9.080845	6.080423	1.28E-04	4.05E-04	0.7238	42	Middle
CPT_phageK_gp036	2.026064	10.89825	6.138019	1.19E-04	3.87E-04	0.700763	53	Early
CPT_phageK_gp018	2.026064	10.89825	6.138019	1.19E-04	3.87E-04	0.700763	5	Early
CPT_phageK_gp029	2.170935	9.571575	5.789729	1.89E-04	5.45E-04	0.286556	6	Middle
CPT_phageK_gp164	2.234009	4.244211	4.543346	0.001122	0.002134	-1.09761	30	Middle
CPT_phageK_gp057	2.290576	8.877284	6.414311	8.37E-05	2.97E-04	1.190927	48	Middle
CPT_phageK_gp063	2.364673	7.637053	8.475874	7.99E-06	5.20E-05	3.812441	48	Middle
CPT_phageK_gp094	3.043087	4.303047	9.102898	4.27E-06	3.02E-05	4.724177	45	Middle
CPT_phageK_gp191	3.249538	6.33537	6.557138	7.00E-05	2.60E-04	1.624563	23	Late
CPT_phageK_gp061	3.576094	6.560325	6.490324	7.61E-05	2.74E-04	1.529752	48	Middle
CPT_phageK_gp083	3.61922	9.584673	5.423462	3.11E-04	7.90E-04	-0.26518	45	Middle

Table S3C: These Log2FC values were calculated comparing the counts from 4 replicates of RNA samples collected from phage K infections of NRS384WT and *rpoC* G17D at 10 min. Only genes showing a Log2FC <-1 or >1 are being shown here.

**geneID**	**log2FC**	**AveExpr**	**t**	**P.Value**	**adj.P.Val**	**B**	**TU#**	**Classification**
CPT_phageK_gp189	-6.95409	3.140442	-21.5372	7.31E-11	1.70E-08	14.47934	24	Late
CPT_phageK_gp188	-6.50797	3.115669	-19.6769	2.08E-10	2.42E-08	13.66753	24	Late
CPT_phageK_gp173	-3.61947	13.53728	-11.1968	1.18E-07	3.23E-06	7.932388	27	Late
CPT_phageK_gp175	-3.11743	10.93754	-15.9054	2.37E-09	1.84E-07	12.01648	27	Late
CPT_phageK_gp174	-2.83678	11.90861	-14.4073	7.24E-09	4.22E-07	10.83952	27	Late
CPT_phageK_gp166	-2.77711	12.79261	-8.89461	1.38E-06	2.29E-05	5.316776	28	Late
CPT_phageK_gp185	-2.65638	7.710995	-9.72215	5.41E-07	1.05E-05	6.604669	25	Late

CPT_phageK_gp167	-2.57545	6.84177	-8.31585	2.76E-06	3.57E-05	4.992533	28	Late
CPT_phageK_gp183	-2.50669	12.37799	-12.3426	4.06E-08	1.35E-06	9.023372	25	Late
CPT_phageK_gp168	-2.48812	9.66041	-13.0074	2.27E-08	1.06E-06	9.744496	28	Late
CPT_phageK_gp186	-2.30648	8.654316	-11.1427	1.25E-07	3.23E-06	8.05445	25	Late
CPT_phageK_gp171	-2.27487	10.03795	-8.71472	1.70E-06	2.51E-05	5.200205	28	Late
CPT_phageK_gp165	-2.23328	9.71027	-12.6446	3.11E-08	1.21E-06	9.412483	29	Late
CPT_phageK_gp184	-2.19753	8.82621	-10.471	2.45E-07	5.18E-06	7.346916	25	Late
CPT_phageK_gp160	-2.13631	8.414475	-10.5132	2.34E-07	5.18E-06	7.418082	31	Late
CPT_phageK_gp170	-1.98398	9.828605	-8.70397	1.73E-06	2.51E-05	5.20253	28	Late
CPT_phageK_gp177	-1.96098	7.979213	-7.74797	5.65E-06	5.99E-05	4.149312	26	Late
CPT_phageK_gp176	-1.81306	11.65119	-8.61719	1.91E-06	2.62E-05	4.961394	26	Late
CPT_phageK_gp187	-1.75756	6.67371	-3.50801	0.004401	0.020106	-2.56647	24	Late
CPT_phageK_gp227	-1.67302	5.191748	-6.00459	6.53E-05	4.75E-04	1.85283	11	Late
CPT_phageK_gp169	-1.56451	9.054223	-7.3123	1.00E-05	9.75E-05	3.428692	28	Late
CPT_phageK_gt004	-1.53169	6.659188	-4.92962	3.62E-04	0.00222	-0.02039	22	Late
CPT_phageK_gp172	-1.48965	6.197073	-5.06287	2.90E-04	0.001827	0.245841	28	Late
CPT_phageK_gp158	-1.48487	11.4844	-8.92282	1.33E-06	2.29E-05	5.346507	32	Late
CPT_phageK_gp193	-1.3903	9.99827	-6.67203	2.44E-05	1.96E-04	2.392955	21	Late
CPT_phageK_gp159	-1.28392	9.671206	-6.47428	3.25E-05	2.44E-04	2.123829	31	Late
CPT_phageK_gp178	-1.25092	6.05909	-4.2628	0.001135	0.00661	-1.13769	26	Late
CPT_phageK_gp115	-1.23076	11.09286	-4.18686	0.001297	0.007196	-1.84996	41	Late
CPT_phageK_gp116	-1.06484	10.22652	-6.77156	2.12E-05	0.000183	2.521285	41	Late
CPT_phageK_gt003	-1.03383	7.10446	-3.76891	0.002738	0.012759	-2.14034	22	Late
CPT_phageK_gp222	1.050888	7.108743	2.915421	0.01311	0.043016	-3.7085	12	Middle
CPT_phageK_gp096	1.108813	11.73266	7.625641	6.63E-06	6.71E-05	3.641156	45	Middle
CPT_phageK_gp035	1.139621	5.637212	3.441116	0.004974	0.020551	-2.5935	53	Early
CPT_phageK_gp019	1.139621	5.637212	3.441116	0.004974	0.020551	-2.5935	5	Early
CPT_phageK_gp033	1.145276	6.701526	3.440898	0.004976	0.020551	-2.69338	53	Early
CPT_phageK_gp021	1.145276	6.701526	3.440898	0.004976	0.020551	-2.69338	5	Early
CPT_phageK_gp028	1.148935	10.05182	6.6903	2.38E-05	1.96E-04	2.417871	6	Middle
CPT_phageK_gp024	1.190483	9.64208	4.109269	0.001488	0.007879	-1.8517	6	Middle
CPT_phageK_gp085	1.345717	9.494284	4.022619	0.001736	0.008793	-1.98698	45	Middle
CPT_phageK_gp100	1.441085	8.339489	5.146875	0.000253	0.001636	0.158385	44	Late
CPT_phageK_gp081	1.47437	5.859769	4.033078	0.001704	0.008793	-1.53402	45	Middle
CPT_phageK_gp219	1.476257	10.01219	5.530437	1.36E-04	9.33E-04	0.594755	12	Middle
CPT_phageK_gp041	1.591415	8.600109	7.095511	1.35E-05	1.21E-04	3.188991	52	Early
CPT_phageK_gp013	1.591415	8.600109	7.095511	1.35E-05	1.21E-04	3.188991	4	Early
CPT_phageK_gp062	1.593018	8.884325	5.539492	1.34E-04	9.33E-04	0.757707	48	Middle
CPT_phageK_gp023	1.605499	4.17986	2.929268	0.012779	0.042535	-3.39969	6	Middle
CPT_phageK_gp134	1.627074	12.69829	7.890624	4.70E-06	5.22E-05	4.000354	37	Middle
CPT_phageK_gp036	1.631099	8.656474	8.148913	3.40E-06	3.96E-05	4.625428	53	Early
CPT_phageK_gp018	1.631099	8.656474	8.148913	3.40E-06	3.96E-05	4.625428	5	Early
CPT_phageK_gp094	1.66847	3.011175	4.242683	0.001176	0.006681	-0.94259	45	Middle
CPT_phageK_gp057	1.692698	6.826897	4.150757	0.001383	0.007491	-1.40602	48	Middle
CPT_phageK_gp029	1.968868	8.491573	6.628108	2.60E-05	2.02E-04	2.521076	6	Middle
CPT_phageK_gp061	2.43953	4.671918	3.949196	0.001979	0.009608	-1.57647	48	Middle
CPT_phageK_gp083	2.451839	8.191723	5.28792	2.01E-04	0.001337	0.427577	45	Middle

**Table S3D**: These Log2FC values were calculated comparing the counts from 4 replicates of RNA samples collected from phage K infections of NRS384WT and *rpoC* G17D at 20 min. Only genes showing a Log2FC <-1 or >1 are being shown here.								
**geneID**	**log2FC**	**AveExpr**	**t**	**P.Value**	**adj.P.Val**	**B**	**TU#**	**Classification**
CPT_phageK_gp188	-10.6798	5.187162	-25.9374	2.98E-11	1.74E-09	15.59814	24	Late
CPT_phageK_gp189	-10.3312	5.578656	-33.6224	1.75E-12	4.10E-10	17.62402	24	Late
CPT_phageK_gp167	-4.71917	9.516326	-27.3854	1.65E-11	1.29E-09	17.02086	28	Late
CPT_phageK_gp168	-3.85193	12.40834	-14.4039	1.65E-08	1.96E-07	9.953249	28	Late
CPT_phageK_gp185	-3.83197	8.210801	-19.1483	7.96E-10	1.55E-08	13.20736	25	Late
CPT_phageK_gp173	-3.78696	16.26752	-20.5656	3.69E-10	1.02E-08	13.95662	27	Late
CPT_phageK_gp165	-3.53104	12.54284	-28.307	1.15E-11	1.29E-09	17.43029	29	Late
CPT_phageK_gp193	-3.40173	11.39037	-18.5976	1.09E-09	1.96E-08	12.80517	21	Late
CPT_phageK_gp166	-3.3777	15.35922	-13.8486	2.50E-08	2.54E-07	9.57174	28	Late
CPT_phageK_gp158	-3.36599	12.57496	-21.1725	2.69E-10	9.01E-09	14.246	32	Late
CPT_phageK_gp172	-3.23828	8.992401	-9.54838	1.13E-06	8.52E-06	5.618594	28	Late
CPT_phageK_gp175	-3.22595	13.11441	-16.7916	3.25E-09	5.07E-08	11.66205	27	Late
CPT_phageK_gp171	-3.214	11.71667	-20.2665	4.32E-10	1.02E-08	13.75759	28	Late
CPT_phageK_gp184	-3.19101	9.407485	-22.6019	1.33E-10	5.18E-09	14.99315	25	Late
CPT_phageK_gp160	-3.09589	10.27763	-16.1273	5.00E-09	6.50E-08	11.24817	31	Late
CPT_phageK_gp174	-3.04442	14.3719	-16.3475	4.32E-09	5.95E-08	11.38621	27	Late
CPT_phageK_gp187	-2.82216	7.026094	-13.4796	3.32E-08	3.23E-07	9.437854	24	Late
CPT_phageK_gp183	-2.74119	13.12584	-20.2444	4.37E-10	1.02E-08	13.74635	25	Late
CPT_phageK_gp116	-2.73501	11.08517	-24.6011	5.30E-11	2.48E-09	15.90384	41	Late
CPT_phageK_gp186	-2.73218	9.156548	-16.4493	4.05E-09	5.92E-08	11.51612	25	Late
CPT_phageK_gp114	-2.64032	7.339526	-11.8004	1.32E-07	1.14E-06	7.987563	41	Late
CPT_phageK_gp154	-2.63189	9.69871	-19.7797	5.62E-10	1.19E-08	13.52891	34	Middle
CPT_phageK_gp159	-2.62905	11.63321	-12.7728	5.82E-08	5.24E-07	8.598701	31	Late
CPT_phageK_gp170	-2.48726	11.69136	-14.0405	2.16E-08	2.35E-07	9.64575	28	Late
CPT_phageK_gp169	-2.25195	10.9623	-17.1621	2.57E-09	4.30E-08	11.89633	28	Late
CPT_phageK_gp191	-2.25145	3.158117	-7.03477	2.12E-05	1.24E-04	2.992403	23	Late
CPT_phageK_gp195	-2.18705	13.96953	-14.0121	2.21E-08	2.35E-07	9.656109	21	Late
CPT_phageK_gp227	-2.18249	6.361641	-7.96314	6.63E-06	4.31E-05	3.945321	11	Late
CPT_phageK_gp115	-2.05876	12.04267	-11.3392	1.99E-07	1.66E-06	7.281777	41	Late
CPT_phageK_gp153	-1.95736	8.937647	-10.3557	5.01E-07	3.90E-06	6.459208	34	Middle
CPT_phageK_gp126	-1.84338	7.51679	-8.61695	3.10E-06	2.20E-05	4.660395	38	Late
CPT_phageK_gp177	-1.793	9.077611	-11.1597	2.34E-07	1.89E-06	7.249122	26	Late
CPT_phageK_gp152	-1.74646	10.31336	-7.69842	9.14E-06	5.78E-05	3.242163	34	Middle
CPT_phageK_gp151	-1.70746	11.31717	-12.8633	5.41E-08	5.06E-07	8.665729	34	Middle
CPT_phageK_gp176	-1.6544	12.93424	-14.3863	1.67E-08	1.96E-07	9.918868	26	Late
CPT_phageK_gp157	-1.51132	11.79279	-8.29177	4.50E-06	3.01E-05	3.944283	33	Middle
CPT_phageK_gp178	-1.12394	7.764451	-5.08301	3.48E-04	0.001455	-0.34219	26	Late
CPT_phageK_gp156	-1.10398	10.54234	-5.97987	9.01E-05	4.69E-04	0.783191	33	Middle
CPT_phageK_gp022	1.059069	7.685487	5.680595	1.40E-04	6.41E-04	0.623172	6	Middle
CPT_phageK_gp233	1.076102	9.638191	4.945617	4.33E-04	0.001746	-0.79396	9	Middle
CPT_phageK_gp028	1.151539	8.851457	4.387565	0.001074	0.004052	-1.64817	6	Middle

CPT_phageK_gp029	1.180321	7.807267	3.598429	0.004151	0.013313	-2.9211	6	Middle
CPT_phageK_gp083	1.266166	7.252752	3.37187	0.006193	0.018114	-3.27101	45	Middle
CPT_phageK_gp025	1.276432	10.03638	8.352461	4.19E-06	2.89E-05	4.088736	6	Middle
CPT_phageK_gt003	1.361044	7.334544	5.536878	1.73E-04	7.65E-04	0.431182	22	Late
CPT_phageK_gp225	1.36298	12.32925	9.20926	1.62E-06	1.18E-05	5.042034	12	Middle
CPT_phageK_gp036	1.371678	7.74925	5.75328	1.25E-04	5.99E-04	0.729437	53	Early
CPT_phageK_gp018	1.371678	7.74925	5.75328	1.25E-04	5.99E-04	0.729437	5	Early
CPT_phageK_gp081	1.386548	4.851899	4.148863	0.001604	0.005864	-1.66744	45	Middle
CPT_phageK_gp023	1.55649	3.867506	5.995374	8.81E-05	4.69E-04	1.446488	6	Middle
CPT_phageK_gp094	1.750261	1.751066	4.411544	0.001032	0.003957	-0.88367	45	Middle
CPT_phageK_gt001	1.923386	9.940346	5.50702	1.81E-04	7.85E-04	0.119872	8	Late
CPT_phageK_gt002	2.153497	4.937936	6.519658	4.22E-05	2.41E-04	2.127533	22	Late

Table S3E: These Log2FC values were calculated comparing the counts from 4 replicates of RNA samples collected from phage K infections of NRS384WT and *rpoC* G17D at 30 min. Only genes showing a Log2FC <-1 or >1 are being shown here.

**geneID**	**log2FC**	**AveExpr**	**t**	**P.Value**	**adj.P.Val**	**B**	**TU#**	**Classification**
CPT_phageK_gp189	-11.0447	6.473854	-27.4532	1.75E-10	1.42E-09	14.67477	24	Late
CPT_phageK_gp188	-10.6226	6.268955	-27.8642	1.52E-10	1.37E-09	14.79689	24	Late
CPT_phageK_gp190	-9.89746	3.441781	-27.6757	1.62E-10	1.37E-09	14.62494	24	Late
CPT_phageK_gp167	-6.21348	9.76223	-38.2369	7.35E-12	1.04E-10	17.99222	28	Late
CPT_phageK_gp168	-6.00525	12.30684	-47.8628	8.51E-13	2.86E-11	20.11291	28	Late
CPT_phageK_gp193	-5.83047	11.6626	-40.7478	3.99E-12	8.53E-11	18.60696	21	Late
CPT_phageK_gp166	-5.55432	15.92801	-53.3201	3.01E-13	1.77E-11	21.1922	28	Late
CPT_phageK_gp165	-5.44151	13.18427	-27.9314	1.48E-10	1.37E-09	14.87434	29	Late
CPT_phageK_gp116	-5.43803	12.33092	-68.9294	2.54E-14	2.99E-12	23.28017	41	Late
CPT_phageK_gp172	-5.2766	8.959121	-47.8662	8.50E-13	2.86E-11	19.98223	28	Late
CPT_phageK_gp173	-5.1054	16.57761	-48.939	6.87E-13	2.86E-11	20.36899	27	Late
CPT_phageK_gp114	-5.06498	8.539313	-29.4229	9.03E-11	8.84E-10	15.49673	41	Late
CPT_phageK_gp175	-4.72256	12.98084	-46.3435	1.16E-12	3.41E-11	19.84206	27	Late
CPT_phageK_gp227	-4.72166	6.683004	-22.8946	9.84E-10	7.46E-09	13.10168	11	Late
CPT_phageK_gp171	-4.69509	12.00171	-27.6583	1.63E-10	1.37E-09	14.82123	28	Late
CPT_phageK_gp115	-4.64248	13.51122	-59.2186	1.10E-13	8.60E-12	22.12767	41	Late
CPT_phageK_gp195	-4.58585	14.43617	-40.3128	4.43E-12	8.67E-11	18.4823	21	Late
CPT_phageK_gp185	-4.45112	7.817437	-29.6769	8.32E-11	8.50E-10	15.58145	25	Late
CPT_phageK_gp158	-4.32147	12.2396	-38.5682	6.76E-12	1.04E-10	18.08481	32	Late
CPT_phageK_gp174	-4.30169	14.27288	-72.7065	1.52E-14	2.99E-12	23.98883	27	Late
CPT_phageK_gp187	-4.01814	6.756069	-10.7742	1.08E-06	4.70E-06	5.691677	24	Late
CPT_phageK_gp170	-3.99209	11.63317	-41.8285	3.11E-12	8.11E-11	18.858	28	Late
CPT_phageK_gp169	-3.61188	10.81204	-33.3997	2.69E-11	3.26E-10	16.70404	28	Late
CPT_phageK_gp154	-3.61005	9.654087	-38.5531	6.79E-12	1.04E-10	18.06323	34	Middle
CPT_phageK_gp191	-3.55196	3.631294	-14.4063	7.62E-08	4.37E-07	8.744126	23	Late
CPT_phageK_gp160	-3.52619	9.672238	-24.4446	5.28E-10	4.14E-09	13.66149	31	Late
CPT_phageK_gp178	-3.50865	7.982801	-11.1117	8.19E-07	3.82E-06	5.89903	26	Late
CPT_phageK_gp152	-3.36837	9.858312	-35.5234	1.49E-11	1.94E-10	17.29694	34	Middle
CPT_phageK_gp184	-3.31879	8.810171	-30.0448	7.39E-11	7.90E-10	15.69757	25	Late

CPT_phageK_gp159	-3.27555	11.32358	-38.1584	7.49E-12	1.04E-10	17.98415	31	Late
CPT_phageK_gp153	-3.1768	8.557378	-22.2854	1.27E-09	9.34E-09	12.78234	34	Middle
CPT_phageK_gp100	-3.15269	9.001319	-15.8825	3.08E-08	1.86E-07	9.389004	44	Late
CPT_phageK_gp183	-3.1061	12.47242	-41.1763	3.61E-12	8.48E-11	18.71233	25	Late
CPT_phageK_gp186	-3.04517	8.456402	-11.0093	8.90E-07	4.02E-06	5.793087	25	Late
CPT_phageK_gp151	-2.82944	11.01037	-31.0731	5.36E-11	6.00E-10	15.99544	34	Middle
CPT_phageK_gp157	-2.51415	11.21619	-33.2908	2.77E-11	3.26E-10	16.66417	33	Middle
CPT_phageK_gp226	-2.46436	8.85847	-21.8832	1.51E-09	1.04E-08	12.59301	11	Late
CPT_phageK_gp210	-2.44539	12.61718	-17.2577	1.42E-08	9.00E-08	9.966227	15	Late
CPT_phageK_gp177	-2.31194	8.789126	-14.9899	5.28E-08	3.10E-07	8.820888	26	Late
CPT_phageK_gp124	-2.14105	6.842295	-6.49234	8.20E-05	2.47E-04	0.970322	39	Middle
CPT_phageK_gp176	-2.12734	12.53095	-22.0133	1.43E-09	1.02E-08	12.44199	26	Late
CPT_phageK_gp150	-2.11364	13.151	-38.2094	7.40E-12	1.04E-10	17.96863	34	Middle
CPT_phageK_gp211	-1.94626	7.52567	-20.4838	2.82E-09	1.90E-08	11.96509	15	Late
CPT_phageK_gp156	-1.83672	9.794918	-12.7722	2.31E-07	1.26E-06	7.195846	33	Middle
CPT_phageK_gp155	-1.62231	12.47574	-19.6137	4.25E-09	2.78E-08	11.26652	33	Middle
CPT_phageK_gp099	-1.35342	7.544303	-11.6126	5.50E-07	2.75E-06	6.342481	45	Middle
CPT_phageK_gp212	-1.33066	8.600817	-9.05351	5.04E-06	1.97E-05	3.898292	15	Late
CPT_phageK_gp126	-1.31553	6.866955	-10.9086	9.67E-07	4.29E-06	5.757341	38	Late
CPT_phageK_gp147	-1.23964	6.385356	-8.10446	1.31E-05	4.53E-05	2.966524	35	Middle
CPT_phageK_gp082	-1.09992	10.72128	-5.11759	5.06E-04	0.001265	-1.19648	45	Middle
CPT_phageK_gp071	-1.09897	9.709002	-3.108	0.011568	0.021074	-4.38476	46	Middle
CPT_phageK_gp022	1.051427	7.263957	7.471398	2.60E-05	8.15E-05	2.194025	6	Middle
CPT_phageK_gp030	1.071552	9.282155	11.38708	6.57E-07	3.21E-06	6.073563	6	Middle
CPT_phageK_gp192	1.086866	7.58644	3.814025	0.003635	0.007492	-3.10062	22	Late
CPT_phageK_gp120	1.12238	14.88253	11.92214	4.33E-07	2.26E-06	6.13707	39	Middle
CPT_phageK_gp217	1.131521	10.46716	4.270173	0.001775	0.003912	-2.51631	13	Middle
CPT_phageK_gp233	1.137516	8.769715	7.401435	2.81E-05	8.70E-05	2.042964	9	Middle
CPT_phageK_gp119	1.153229	13.68077	14.22391	8.58E-08	4.80E-07	7.956722	40	Middle
CPT_phageK_gp094	1.169234	1.070682	3.05772	0.012584	0.022575	-3.65765	45	Middle
CPT_phageK_gp061	1.241189	2.322418	6.227746	1.14E-04	3.31E-04	1.176953	48	Middle
CPT_phageK_gp224	1.3379	10.53726	12.40985	3.01E-07	1.61E-06	6.840036	12	Middle
CPT_phageK_gp117	1.338929	11.70028	7.557482	2.36E-05	7.52E-05	2.019413	40	Middle
CPT_phageK_gp036	1.442038	7.510397	11.09769	8.28E-07	3.82E-06	5.908312	53	Early
CPT_phageK_gp018	1.442038	7.510397	11.09769	8.28E-07	3.82E-06	5.908312	5	Early
CPT_phageK_gp041	1.447128	7.499179	10.65261	1.20E-06	5.02E-06	5.512391	52	Early
CPT_phageK_gp013	1.447128	7.499179	10.65261	1.20E-06	5.02E-06	5.512391	4	Early
CPT_phageK_gp225	1.484582	11.25423	9.252502	4.17E-06	1.66E-05	3.940902	12	Middle
CPT_phageK_gp028	1.637949	8.112746	16.71253	1.91E-08	1.18E-07	9.930867	6	Middle
CPT_phageK_gp029	1.662636	7.08669	8.241074	1.14E-05	4.04E-05	3.123824	6	Middle
CPT_phageK_gt001	1.792491	9.258607	10.27299	1.65E-06	6.82E-06	5.06992	22	Late
CPT_phageK_gp023	1.820531	3.574468	5.70321	2.26E-04	6.25E-04	0.315032	6	Middle
CPT_phageK_gp025	1.896235	8.992476	7.997793	1.47E-05	4.99E-05	2.727426	6	Middle
CPT_phageK_gt003	1.999873	6.512747	6.44053	8.74E-05	2.60E-04	0.991106	22	Late
CPT_phageK_gt002	2.613126	4.487327	9.991018	2.12E-06	8.58E-06	5.21119	22	Late

Table S3F: These Log2FC values were calculated comparing the counts from 4 replicates of RNA samples collected from phage K infections of NRS384WT and *rpoC* G17D at 40 min. Only genes showing a Log2FC <-1 or >1 are being shown here.

geneID	log2FC	AveExpr	t	P.Value	adj.P.Val	B	TU#	Classification
CPT_phageK_gp188	-9.66617	6.971825	-44.6465	1.27E-13	2.71E-12	20.74063	24	Late
CPT_phageK_gp189	-9.41613	7.52441	-60.8535	4.50E-15	2.65E-13	23.19018	24	Late
CPT_phageK_gp190	-9.14787	3.997929	-24.1668	9.15E-11	6.15E-10	15.03091	24	Late
CPT_phageK_gp167	-6.27469	9.469475	-19.4612	9.08E-10	5.08E-09	12.96352	28	Late
CPT_phageK_gp227	-6.18345	7.215211	-23.6108	1.17E-10	7.50E-10	15.1365	11	Late
CPT_phageK_gp165	-6.06049	13.27455	-37.8592	7.49E-13	1.04E-11	20.14955	29	Late
CPT_phageK_gp193	-5.89808	12.11368	-50.8657	3.12E-14	1.02E-12	23.32404	21	Late
CPT_phageK_gp114	-5.70708	9.368117	-26.0237	4.16E-11	3.15E-10	16.14862	41	Late
CPT_phageK_gp168	-5.70037	12.27919	-71.0243	8.49E-16	9.97E-14	26.66781	28	Late
CPT_phageK_gp116	-5.66454	12.87642	-76.7343	3.68E-16	8.65E-14	27.44618	41	Late
CPT_phageK_gp166	-5.2779	16.09489	-54.1307	1.59E-14	6.24E-13	24.00991	28	Late
CPT_phageK_gp172	-5.09189	8.828031	-29.6312	1.04E-11	9.04E-11	17.55418	28	Late
CPT_phageK_gp115	-4.66948	14.43999	-50.3482	3.48E-14	1.02E-12	23.24187	41	Late
CPT_phageK_gp175	-4.5874	13.02931	-40.9078	3.26E-13	5.47E-12	20.9982	27	Late
CPT_phageK_gp191	-4.55655	3.566962	-17.9286	2.15E-09	1.12E-08	12.19586	23	Late
CPT_phageK_gp100	-4.55239	9.444115	-21.3381	3.43E-10	1.97E-09	13.93133	44	Late
CPT_phageK_gp195	-4.5273	14.7267	-48.6326	5.06E-14	1.32E-12	22.8711	21	Late
CPT_phageK_gp173	-4.50097	16.62428	-41.1042	3.09E-13	5.47E-12	21.04401	27	Late
CPT_phageK_gp158	-4.3885	12.3953	-62.3066	3.49E-15	2.65E-13	25.42533	32	Late
CPT_phageK_gp174	-4.32566	14.29469	-58.1317	7.38E-15	3.47E-13	24.75183	27	Late
CPT_phageK_gp185	-4.25536	7.701858	-12.6685	7.86E-08	3.13E-07	8.319364	25	Late
CPT_phageK_gp171	-4.21807	11.79813	-40.1053	4.03E-13	6.32E-12	20.79152	28	Late
CPT_phageK_gp154	-3.96975	9.647397	-45.7754	9.71E-14	2.28E-12	22.14462	34	Middle
CPT_phageK_gp226	-3.91814	9.057802	-25.4214	5.33E-11	3.92E-10	15.8766	11	Late
CPT_phageK_gp170	-3.76131	11.60013	-43.1792	1.82E-13	3.57E-12	21.59141	28	Late
CPT_phageK_gp184	-3.61452	8.663721	-31.7639	4.93E-12	5.27E-11	18.29603	25	Late
CPT_phageK_gp178	-3.60112	7.642425	-22.1255	2.34E-10	1.37E-09	14.41525	26	Late
CPT_phageK_gp153	-3.54611	8.71016	-30.2167	8.42E-12	7.91E-11	17.76252	34	Middle
CPT_phageK_gp169	-3.45188	10.69084	-39.5533	4.68E-13	6.87E-12	20.64719	28	Late
CPT_phageK_gp210	-3.43697	12.78207	-18.6587	1.41E-09	7.55E-09	12.18745	15	Late
CPT_phageK_gp187	-3.40084	6.428046	-15.9741	7.20E-09	3.38E-08	10.94826	24	Late
CPT_phageK_gp211	-3.36696	7.61033	-24.4298	8.15E-11	5.80E-10	15.48887	15	Late
CPT_phageK_gp152	-3.26858	9.869957	-23.1364	1.45E-10	8.76E-10	14.75359	34	Middle
CPT_phageK_gp186	-3.25363	8.796901	-24.1618	9.17E-11	6.15E-10	15.31916	25	Late
CPT_phageK_gp159	-3.2154	11.36921	-29.3463	1.15E-11	9.66E-11	17.33666	31	Late
CPT_phageK_gp151	-3.19517	11.10373	-36.9002	9.87E-13	1.18E-11	19.8841	34	Middle
CPT_phageK_gp160	-3.16953	9.466043	-36.8915	9.90E-13	1.18E-11	19.89965	31	Late
CPT_phageK_gp183	-3.0366	12.4793	-36.8551	1.00E-12	1.18E-11	19.85291	25	Late
CPT_phageK_gp157	-2.69503	11.29007	-30.3352	8.07E-12	7.91E-11	17.70404	33	Middle
CPT_phageK_gp177	-2.42453	8.512645	-23.2134	1.40E-10	8.68E-10	14.88283	26	Late
CPT_phageK_gp150	-2.35804	13.11604	-31.9311	4.66E-12	5.22E-11	18.24858	34	Middle
CPT_phageK_gp156	-2.24864	9.875294	-30.9434	6.53E-12	6.67E-11	17.9703	33	Middle
CPT_phageK_gp176	-2.18948	12.40529	-28.8332	1.39E-11	1.09E-10	17.11086	26	Late

CPT_phageK_gp155	-2.0979	12.48696	-29.0305	1.29E-11	1.05E-10	17.18621	33	Middle
CPT_phageK_gp099	-2.06746	7.533339	-11.5816	1.95E-07	7.17E-07	7.34767	45	Middle
CPT_phageK_gp212	-2.02399	8.616275	-5.73417	1.40E-04	3.04E-04	0.153821	15	Late
CPT_phageK_gp124	-2.01778	5.860316	-2.88677	0.015017	0.024506	-4.27598	39	Middle
CPT_phageK_gp164	-1.87601	1.062542	-6.22316	6.98E-05	1.62E-04	1.806894	30	Middle
CPT_phageK_gp147	-1.78074	6.167894	-11.2101	2.71E-07	9.62E-07	7.15847	35	Middle
CPT_phageK_gp031	-1.6571	10.99093	-16.6309	4.73E-09	2.36E-08	10.93313	7	Late
CPT_phageK_gp098	-1.314	9.99768	-12.8464	6.82E-08	2.81E-07	8.127017	45	Middle
CPT_phageK_gp093	-1.30494	6.435205	-7.35907	1.57E-05	4.14E-05	2.820005	45	Middle
CPT_phageK_gp145	-1.24369	10.03468	-11.1964	2.74E-07	9.62E-07	6.624428	35	Middle
CPT_phageK_gp132	-1.22405	9.700971	-16.4088	5.44E-09	2.61E-08	10.87137	37	Middle
CPT_phageK_gp149	-1.14994	12.50185	-11.5212	2.06E-07	7.44E-07	6.826872	34	Middle
CPT_phageK_gp103	-1.13193	9.978784	-11.9134	1.47E-07	5.47E-07	7.303026	43	Middle
CPT_phageK_gp097	-1.11856	5.664106	-6.22753	6.93E-05	1.62E-04	1.325023	45	Middle
CPT_phageK_gp032	-1.07932	10.98893	-9.24868	1.81E-06	5.83E-06	4.523989	7	Late
CPT_phageK_gp182	-1.02465	10.98107	-12.0479	1.31E-07	4.96E-07	7.354494	25	Late
CPT_phageK_gp111	1.102737	13.09412	13.65901	3.64E-08	1.55E-07	8.699824	42	Middle
CPT_phageK_gp042	1.104779	2.05403	3.726711	0.003435	0.006115	-2.30323	51	Early
CPT_phageK_gp012	1.104779	2.05403	3.726711	0.003435	0.006115	-2.30323	3	Early
CPT_phageK_gp213	1.132728	10.42941	13.89014	3.06E-08	1.33E-07	8.977023	14	Middle
CPT_phageK_gp029	1.1678	6.874239	5.935384	1.05E-04	2.36E-04	0.759393	6	Middle
CPT_phageK_gt004	1.239015	5.897375	4.036679	0.002024	0.003716	-2.21755	22	Late
CPT_phageK_gp036	1.266567	7.454213	4.644112	7.43E-04	0.001455	-1.38351	53	Early
CPT_phageK_gp018	1.266567	7.454213	4.644112	7.43E-04	0.001455	-1.38351	5	Early
CPT_phageK_gp028	1.270996	7.968449	6.240781	6.81E-05	1.62E-04	1.060827	6	Middle
CPT_phageK_gp030	1.313495	8.987899	12.82322	6.95E-08	2.81E-07	8.27219	6	Middle
CPT_phageK_gp118	1.400259	8.602659	12.18067	1.17E-07	4.52E-07	7.774706	40	Middle
CPT_phageK_gp224	1.420688	10.01698	15.52909	9.66E-09	4.45E-08	10.26041	12	Middle
CPT_phageK_gp192	1.481396	7.340487	7.819942	8.94E-06	2.50E-05	3.318935	22	Late
CPT_phageK_gp225	1.487673	10.86631	13.00796	6.00E-08	2.52E-07	8.229027	12	Middle
CPT_phageK_gp026	1.491497	8.281186	13.99068	2.84E-08	1.26E-07	9.337477	6	Middle
CPT_phageK_gp022	1.546029	6.809279	9.897733	9.33E-07	3.09E-06	5.789371	6	Middle
CPT_phageK_gp233	1.617367	8.56045	10.02953	8.19E-07	2.79E-06	5.705255	9	Middle
CPT_phageK_gp025	1.85327	8.829636	16.56836	4.92E-09	2.41E-08	11.14598	6	Middle
CPT_phageK_gp117	1.932946	11.503	23.59192	1.18E-10	7.50E-10	14.88243	40	Middle
CPT_phageK_gt003	2.132208	6.449057	7.431899	1.43E-05	3.87E-05	2.94005	22	Late
CPT_phageK_gt001	2.135997	9.244799	17.82031	2.29E-09	1.17E-08	11.91799	8	Late
CPT_phageK_gp119	2.230759	13.46651	29.9149	9.37E-12	8.47E-11	17.52761	40	Middle
CPT_phageK_gp120	2.271522	14.63913	18.96688	1.19E-09	6.51E-09	12.38216	39	Middle
CPT_phageK_gp023	2.337656	3.411271	9.215863	1.87E-06	5.95E-06	5.3928	6	Middle
CPT_phageK_gt002	2.709817	4.92721	8.783458	2.98E-06	9.09E-06	4.792981	22	Late
